# Total synthesis and mechanism of action of the antibiotic armeniaspirol A†

**DOI:** 10.1039/d1sc04290d

**Published:** 2021-11-24

**Authors:** Nanaji Arisetti, Hazel L. S. Fuchs, Janetta Coetzee, Manuel Orozco, Dominik Ruppelt, Armin Bauer, Dominik Heimann, Eric Kuhnert, Satya P. Bhamidimarri, Jayesh A. Bafna, Bettina Hinkelmann, Konstantin Eckel, Stephan A. Sieber, Peter P. Müller, Jennifer Herrmann, Rolf Müller, Mathias Winterhalter, Claudia Steinem, Mark Brönstrup

**Affiliations:** Department of Chemical Biology, Helmholtz Centre for Infection Research Inhoffenstrasse 7 38124 Braunschweig Germany mark.broenstrup@helmholtz-hzi.de; German Centre for Infection Research Partner Site Hannover-Braunschweig Germany; Helmholtz Institute for Pharmaceutical Research Saarland (HIPS), Helmholtz Centre for Infection Research Saarland University Campus E8.1 66123 Saarbrücken Germany; Georg-August-Universität Göttingen, Institute of Organic and Biomolecular Chemistry Tammannstraße 2 37077 Göttingen Germany; Sanofi R&D Industriepark Höchst 65926 Frankfurt Germany; Jacobs University Bremen Campus Ring 1 28759 Bremen Germany; Department of Chemistry, Chair of Organic Chemistry II, Center for Functional Protein Assemblies (CPA), Technische Universität München Ernst-Otto-Fischer-Straße 8 85748 Garching Germany; Max-Planck-Institute for Dynamics and Self Organization Am Faßberg 17 37077 Göttingen Germany; Center for Biomolecular Drug Research (BMWZ), Leibniz Universität 30159 Hannover Germany

## Abstract

Emerging antimicrobial resistance urges the discovery of antibiotics with unexplored, resistance-breaking mechanisms. Armeniaspirols represent a novel class of antibiotics with a unique spiro[4.4]non-8-ene scaffold and potent activities against Gram-positive pathogens. We report a concise total synthesis of (±) armeniaspirol A in six steps with a yield of 20.3% that includes the formation of the spirocycle through a copper-catalyzed radical cross-coupling reaction. In mechanistic biological experiments, armeniaspirol A exerted potent membrane depolarization, accounting for the pH-dependent antibiotic activity. Armeniaspirol A also disrupted the membrane potential and decreased oxygen consumption in mitochondria. In planar lipid bilayers and in unilamellar vesicles, armeniaspirol A transported protons across membranes in a protein-independent manner, demonstrating that armeniaspirol A acted as a protonophore. We provide evidence that this mechanism might account for the antibiotic activity of multiple chloropyrrole-containing natural products isolated from various origins that share a 4-acylphenol moiety coupled to chloropyrrole as a joint pharmacophore. We additionally describe an efflux-mediated mechanism of resistance against armeniaspirols.

## Abstract 2

The antibiotic armeniaspirol A depolarized bacterial and mammalian cell membranes through a protonophore activity, that accounts for its potent antibiotic effects. A total synthesis of (±) armeniaspirol A was achieved in six steps.

## Graphical abstract

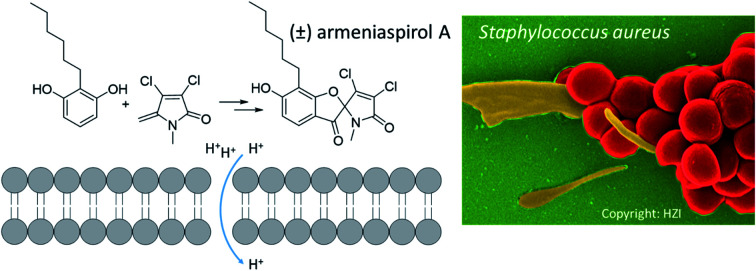

## Introduction

**Fig. 1:**
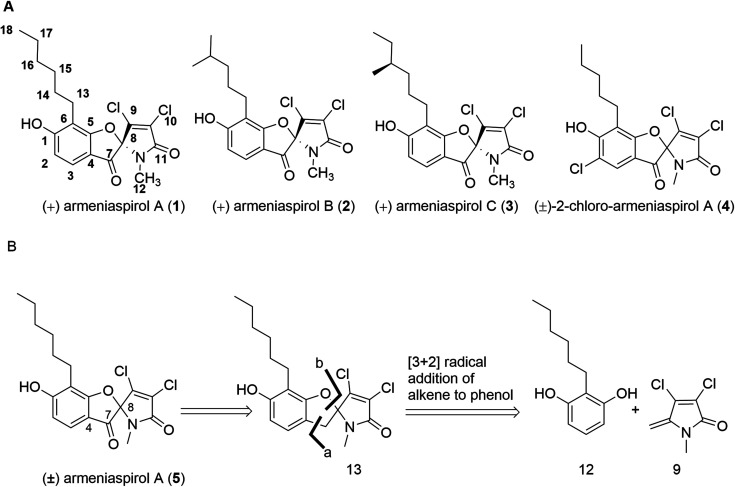
(A) Structures of natural armeniaspirols A–C (1–3) and of (±)-2-chloro-armeniaspirol A (4); (B) retrosynthetic disconnection of (±)-armeniaspirol A.

The rise of bacterial pathogens that are resistant to commonly used antibiotics constitutes a serious and global threat for human health.^1^ Therefore, efforts to overcome antibiotic resistance have been intensified and crowned with the success of increased approval rates,^2^ but they are mostly focused on the optimization of or the re-sensitization to established core structures.^3^ The focus on existing structures is also due to a shortage of novel, bioactive chemical matter, and consequently, there is a need to discover novel chemical scaffolds with antibiotic properties.^4–6^ In this context, we have reported the isolation of the natural products armeniaspirols A–C (1–3) produced by *Streptomyces armeniacus*, that have a unique chlorinated spiro[4.4]non-8-ene scaffold (Fig. 1A).^7^ Armeniaspirols displayed potent activities against Gram-positive pathogens including methicillin-sensitive and methicillin-resistant *Staphylococcus aureus* (MSSA and MRSA) and vancomycin-resistant *Enterococcus faecium* (VRE). Interestingly, mutants resistant to 1 could not be generated by serial passaging in *S. aureus*, and 2 improved the survival rate of mice infected with MRSA. A recent study reported *in vivo* efficacy of 1 in a *Helicobacter pylori* mouse infection model, and the disruption of the *H. pylori* cell membrane was observed by electron microscopy after treatment with 1.^8^ However, the understanding of armeniaspirol's activity has been hampered by the fact that their mechanism of action has been still unknown. Intriguingly, while we drafted this manuscript, Labana *et al.* published the interaction of the armeniaspirol derivative 4 with the proteases ClpXP and ClpYQ – a mechanism that is different to the ones we report below (see discussion).^9^

In a first synthesis of armeniaspirols,^10^ we have constructed the spiro-[4.4]non-8-ene scaffold through an attack of the phenolic group at C-8 of the benzoylated pyrrole ring, mimicking the presumed and later confirmed biosynthesis of the natural product^11,12^ (ESI Scheme S1†). The synthesis was short (7 steps, 13.8% overall yield), but the oxidative chlorination conditions led to chloro-armeniaspirol 4 rather than the natural product itself, as the chlorination at the pyrrole ring was inevitably associated with an undesired chlorination at the electron-rich C2 position. Recent studies of Sivamuthuraman *et al.* as well as Huang *et al.* showed elegant routes to armeniaspirol-like or -inspired scaffolds, that were however functionalized differently;^13,14^ antibiotic activity of those compounds was not reported.

In this study, we report a short total synthesis of racemic (±) armeniaspirol A 5 that allows an improved access to this class of antibiotics. In addition, we can pinpoint the (or at least one) antibacterial mechanism of action for the compounds as well as two possible mechanisms of resistance in *Escherichia coli* Δ*tolC*.

## Results and discussion

In order to obtain armeniaspirols on a larger scale and to access diverse structural analogs, we aimed at establishing a total synthesis route to 1. Based on the difficulties with an excess chlorination experienced in the previous synthetic route,^10^ we avoided a late stage chlorination step and rather aimed to couple an already chlorinated pyrrole moiety. To this end, the correctly functionalized, commercially available 3,4-dichloro-1-methyl-1*H*-pyrrole-2,5-dione 6 appeared as a well-suited building block (ESI Scheme S1†). In a first synthetic strategy, we envisaged a C7–C8 coupling between the protected benzaldehyde 7 and the carbonyl function of 6 in the key step. An Umpolung of the aldehyde according to Corey and Seebach led to the dithiane, which however could not be lithiated by *n*-BuLi according to a quenching experiment with D_2_O. Alternative attempts, including a McMurry coupling of 6 and 7 using Zn and TiCl_4_ or a samarium iodide-mediated radical coupling both led to the decomposition of starting materials. Next, a Shapiro reaction to achieve the C7–C8 coupling was applied. For this purpose, the aldehyde 7 was treated with methyllithium to form the corresponding secondary alcohol in 96% yield, followed by a Dess–Martin oxidation and the conversion of the methylketone with tosyl hydrazine (80% yield over two steps) to the tosylhydrazone 8 (see the ESI†); however, the desired product was not formed after deprotonation with *n*-BuLi. Informed by these setbacks, we revised our retrosynthetic approach, and planned a coupling of the two aromatic rings between C4 and C7 instead. In this context, an iron-catalyzed oxidative radical coupling of a phenolic oxygen and the *ortho*-position of the phenol ring to a styrene reported by Huang *et al.*^15^ caught our attention, as the spiro-topology of armeniaspirols could be built in a single step with this formal [3 + 2] cycloaddition (Fig. 1 and ESI Scheme S2†). To our knowledge, an application of the method to an exocyclic olefin embedded in a heterocycle has not yet been reported.

**Scheme 1:**
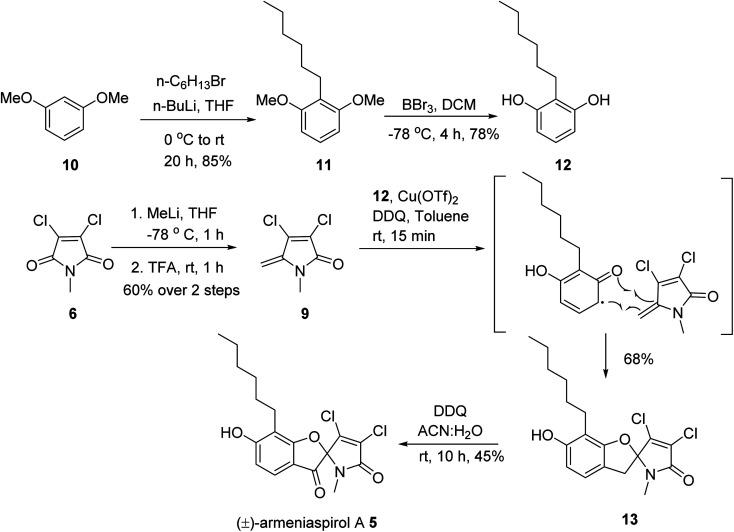
Total synthesis of (±)-armeniaspirol A.

In the forward direction, the olefin substrate 9 was prepared from 6*via* a 1,2 addition of methyl lithium to the carbonyl group, followed by a TFA-catalyzed dehydration in a yield of 60% over two steps (Scheme 1). In a parallel procedure, alkylation of dimethyl resorcinol 10 with *n*-hexylbromide in the presence of *n*-BuLi furnished intermediate 11 in 85% yield. Next, demethylation of 11 with BBr_3_ in DCM at −78 °C provided 6-hexyl resorcinol 12 in 78% yield. In the key step of the synthesis, we attempted an oxidative radical cross coupling/cyclization between 12 and 9 (Table 1). Accordingly, the phenol C-radical generated in the presence of DDQ was subsequently added to 12 to give the desired 5-desoxo-armeniaspirol 13. A reaction for 4 h at room temperature, reflecting conditions of Huang *et al.*,^15^ led to decomposition of the starting material. However, reducing reaction times to 15 min at room temperature led to the formation of 13 in a yield of 45%. Notably, we could improve the yield by replacing the FeCl_3_ catalyst described in the original method by Cu(OTf)_2_. Using 10 mol% of Cu(OTf)_2_ in toluene at room temperature, 13 was obtained in 68% yield (75% brsm). The regioisomeric product, which would arise from the addition of the C-radical to the higher substituted C4-position of 9 was not observed, probably due to a much lower stability of the resulting primary radical intermediate. In the final step, the methylene group at C7 needed to be oxidized to the ketone. While attempts with oxone, sodium chlorite/*t*-BuOOH, pyridinium chlorochromate, potassium persulfate or *m*-CPBA either led to decomposition or to unreacted starting material, the use of DDQ (1.2 eq.) at room temperature in an acetonitrile/water mixture gave (±) armeniaspirol A in a yield of 45% (Table 2). The structural identity was proven by ^13^C- and ^1^H-NMR data and by mixing experiments with natural 1. In summary, we report a short synthesis of racemic armeniaspirol A, that is six steps overall from commercial starting materials and an overall yield of 20.3% in the longest linear sequence. The synthesis relied on an oxidative radical cross coupling/cyclization to access the spirocycle core for (±)-armeniaspirol A, and an additional interesting feature of the synthesis is the late stage, selective oxidation of a benzylic methylene unit.

**Table 1:** Reaction conditions for spirocyclization of 12 and 9 to 13

Entry	Conditions	Result
1	FeCl_3_ (10 mol%), DDQ (1.2 equiv.), toluene, rt, 4 h	Decomposed^a^
2	FeCl_3_ (10 mol%), DDQ (1.2 equiv.), toluene, rt, 15 min	45%^b^
3	Cu(OTf)_2_ (10 mol%), DDQ (1.2 equiv.), toluene, rt, 15 min	68%^b^
4	l-Proline (10 mol%), DDQ (1.2 equiv.), toluene, rt, 15 min	No reaction^c^
5	LiClO_4_ (1.0 equiv.) AcOH : MeCN, Pt/Pt, rt, 0.76 V, 1 h	Decomposed^a^
6	LiClO_4_ (2.0 equiv.) AcOH (1.0 equiv.), MeNO_2_, C/Pt, rt, 0.76 V, 24 h	No reaction^c^
7	Bu_4_NPF_6_ (1.0 equiv.) HFIP : DCM (6 : 4), C/Pt, rt, 10 mA, 24 h	Decomposed^a^
8	Pd(OAc)_2_ (0.1 equiv.) 1,10-phenonthroline (0.2 equiv.), Cu(OAc)_2_(1.0 equiv.), AcONa (3 equiv.), 4 Å ms, 110 °C, 36 h	5%^d^

a Observed many spots on TLC.

b Isolated yields after column chromatography.

c Starting materials were recovered.

d LCMS yield.

**Table 2:** Reaction conditions for the oxidation of 13 to 5

Entry	Conditions	Result
1	KBr (0.5 equiv.), oxone (3 equiv.), MeNO_2_, 50 °C, 24 h	No reaction^a^
2	NaClO_2_ (1.2 equiv.), TBHP (5 equiv.), ACN : H_2_O, 50 °C, 18 h	Decomposed^b^
3	K_2_S_2_O_8_ (3 equiv.),CuSO_4_·5H_2_O (1 equiv.), ACN : H_2_O, 100 °C, 20 min	Decomposed^b^
4	PCC (1.2 equiv.), Celite toluene, rt, 24 h	No reaction^a^
5	*m*-CPBA (2.5 equiv.), NaHCO_3_ (2.0 equiv.), DCM, air, rt, 24 h	No reaction^a^
6	DDQ (1.2 equiv.), DCM : dioxane : H_2_O, rt, 24 h	10%
7	DDQ (1.2 equiv.), 80% aq. MeCN, rt, 10 h	45%
8	TBHP (5.5 M in nonane, 12 equiv.), 130 °C, 10 h	Decomposed^b^

a Starting material was isolated.

b Observed many spots on TLC.

We then set out to shed light on the antibacterial mechanism of action of armeniaspirols. Previous studies consistently reported high activity against Gram-positive pathogens, but a lack of activity against two *E. coli* and two *P. aeruginosa* strains.^7,10^ To investigate the difference in activity, we determined the inhibitory effect of 1 and 2 against the *E. coli* BW25113 wild type strain and the corresponding *E. coli* Δ*tolC* mutant strain, that is devoid of the outer membrane protein channel of the tripartite AcrAB-TolC efflux system. While the minimal inhibitory concentration (MIC) against the wild type strain was >64 μg ml^−1^, it decreased to 2 μg ml^−1^ (1) and 4 μg ml^−1^ (2) against the efflux-deficient mutant. Furthermore, we assessed the MIC against the *E. coli* Δ*acrB* mutant strain JW0451-2 harbouring a deletion of *acrB*, the product of which is located in the inner membrane. Here, no change in susceptibility was noted compared to the wild-type strain. TolC is one of the largest protein structures known, and the loss of TolC induces membrane stress and metabolic imbalance.^16,17^ Therefore, the activity of armeniaspirols against the *E. coli* Δ*tolC* mutant and their inactivity on the *E. coli* ΔacrB mutant is most probably due to outer membrane effects of the TolC deletion that render *E. coli* more permeable. Several studies report that TolC is more important than AcrB in resistance development, suggesting that other transporters interact with TolC.^18,19^ Similar results were obtained when testing another *E. coli* wild type strain (DSM-1116, MICs > 64 μg ml^−1^) and a TolC-deficient mutant strain derived from *E. coli* K12 MG1655 (MICs = 2 μg ml^−1^) (Table 3).

**Table 3:** MICs of 1 and 2 obtained from wild type and mutant *E. coli* strains

Strain	Strain characteristics	MIC (μg mL^−1^)		Source
		Armeniaspirol A (1)	Armeniaspirol B (2)	
*E. coli* BW25113	Wild type strain	>64	>64	Keio collection
*E. coli* DSM-1116	Wild type strain	>64	>64	Deutsche Sammlung von Mikroorganismen und Zellkulturen (DSMZ)
*E. coli* JW5503 (Δ*tolC*)	Δ*tolC*732::kan (*E. coli* BW25113 background)	2	4	Baba *et al.*, 2006 (ref. 44)
*E. coli* JW0451-2 (Δ*acrB*)	Δ*acrB*747(del)::kan (*E. coli* BW25113 background)	>64	>64	Baba *et al.*, 2006
*E. coli* EP664	Δ*tolC* (markerless)	4	8	Valderrama *et al.*, 2019 (ref. 39)
*E. coli* Δ*tolC* pyrr RC1 mutant	Δ*tolC*::kan, selected pyrrolomycin resistance	>64	>64	Valderrama *et al.*, 2019 (ref. 39)
*E. coli* EP676	Δ*tolC* Δ*alsRBACEK-yjcS-ytcA-mdtN*	>64	>64	Valderrama *et al.*, 2019 (ref. 39)
*E. coli* EP680	Δ*tolC* Δ*alsRBACEK-yjcS-ytcA-mdtNOP*::kan	4	8	Valderrama *et al.*, 2019 (ref. 39)
*E. coli* K12 Δ*tolC*	Δ*tolC*	2	2	Donner *et al.*, 2017 (ref. 45)
*E.coli* K12 Δ*tolC* 1^R^ mutant	Δ*tolC; mdtO* (S2K, A3T, L4I, N5G, S6T, L7E, L9C); *prfB* (T173S)	>64	>64	This study

**Fig. 2:**
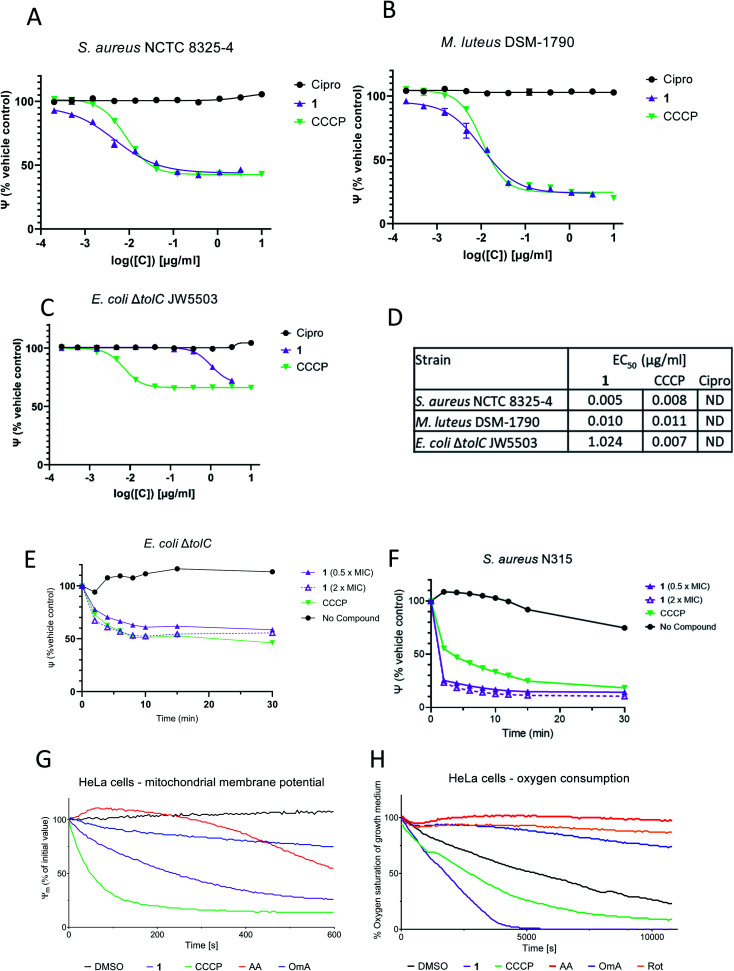
1 causes membrane depolarization. (A–C) Depolarization of the bacterial cytoplasmic membrane by 1 (purple triangles) using the membrane potential sensitive fluorescent dye 3,3′-diethyloxycarbocyanine iodide (DiOC_2_ (3)). The Baclight™ assay was performed in triplicates, in 96 well MTP format. Ciprofloxacin (black circles) and CCCP (green inverted triangles) were used as negative and positive controls, respectively. (A) *S. aureus* NCTC 8325-4; (B) *M. luteus* DSM-1790, (C) *E. coli* JW5503 Δ*tolC*. (D) Summarized EC_50_ values. (E and F) 1 causes depolarization of the bacterial cytoplasmic membrane over time. 1 was applied at 0.5 × MIC (purple triangles; 2 μg ml^−1^) and 2 × MIC (empty purple triangles; 8 μg ml^−1^), and depolarization was assessed by the Baclight™ assay in 96 well format by monitoring the fluorescence shift of DiOC_2_ (3). CCCP (green inverted triangles) and no compound addition (black circles) were used as positive and negative controls, respectively. (E) *E. coli* Δ*tolC*, (F) *S. aureus* N315. (G) Measurement of the inner mitochondrial membrane potential of HeLa cells using the MITO-ID® dye and indicated compounds at 1 μM concentration. (H) Oxygen consumption of HeLa cells in the presence of 1 (10 μM), CCCP (10 μM) and other reference compounds (all 1 μM). Cipro: ciprofloxacin; CCCP: carbonyl cyanide-*m*-chlorphenyl hydrazone; AA: antimycin A; Rot: rotenone; OmA: oligomycin A. MIC: minimum inhibitory concentration.

All armeniaspirols contain a phenolic functional group. In order to investigate its relevance for bioactivity, 1-methoxy-(±)-armeniaspirol A 15 was prepared by *O*-methylation of 5. The compound showed no biological activity on *S. aureus* DSM346 and reduced, but did not inhibit the growth of *M. luteus* DSM1790 (ESI Table S1†). This led to the hypothesis that the protonation state of 1 might influence its antibacterial activity. To test this, the effect of pH on the minimal inhibitory concentration (MIC) of 1 against *Micrococcus luteus* DSM1790 was studied. Interestingly, the MIC of 1 increased drastically from 1.3 μg ml^−1^ at pH 6.5 to 16 μg ml^−1^ at pH 10 (ESI Fig. S1†). The protonation state of 1 changed across this pH range according to computational calculations that predicted an average p*K*_a_ of 7.5 for the molecule (ESI Table S5†). As a protonation/deprotonation equilibrium is a hallmark of antibiotics that function as protonophores, the effect of 1 on the polarization of bacterial membranes was tested. We used a spectrometric assay with the fluorescent dye 3,3′-diethyloxacarbocyanine iodide (DiOC_2_ (3)) with carbonyl cyanide-*m*-chlorophenyl hydrazone (CCCP) as a positive control and ciprofloxacin as a negative control (Fig. 2A–C). Remarkably, 1 was able to induce membrane depolarization in *M. luteus* DSM-1790 and *S. aureus* NCTC 8325-4 (MSSA) at EC_50_ values of 10 ng ml^−1^ and 5 ng ml^−1^, respectively. The positive control CCCP had an equally strong depolarizing effect on both strains at concentrations higher than 41 ng ml^−1^. The MIC values of 1 for *M. luteus* DSM-1790 and MSSA were 1.3 μg ml^−1^ and 0.5 μg ml^−1^, respectively. The apparent difference between EC_50_ and MIC values are ascribed to two factors: first, they reflect differing readouts of the graphs, as the EC_50_ value is the concentration at the inflection point of the curve, whereas the MIC corresponds to the point where the curve reaches the baseline. We noticed that the dose–response curves for armeniaspirols have a relatively shallow slope compared to those of CCCP (see Fig. 2A and B, ESI Fig. S2†) or other antibiotics, which augments the difference between the inflection point and the baseline. Second, the differences reflect that the Baclight™ assay, which detects impairments of the bacterial membrane, is more sensitive than the microbroth dilution method, which measures the compound concentration at which no visible culture growth is present after 24 h. Obviously, to completely inhibit bacterial growth and proliferation, a partial depolarization of the cell (at EC_50_) is insufficient, but a high degree of target engagement is required to exert the cellular phenotype. As the maximum effect of 1 on the depolarization of *M. luteus* and *S. aureus* is approx. 1 μg ml^−1^ and thus in the same range as the MICs, it is plausible that the antibacterial effect is indeed mediated through membrane depolarization. Interestingly, the activity of armeniaspirol A against *M. luteus* DSM1790 was not dependent on the configuration of the stereocenter at C8, because racemic 5 and enantiopure, natural 1 had an identical MIC of 1.25 μg ml^−1^ (ESI Fig. S2†).

For the Gram-negative strain *E. coli* Δ*tolC*, however, the effect of 1 was markedly reduced compared to CCCP (see Fig. 2C). For both compounds, the membrane was only partly depolarized (65% compared to controls), indicating that the inner membrane of *E. coli* Δ*tolC* was not as strongly influenced as those of Gram-positive strains, reflecting different compositions. Further, much higher concentrations of 1 were required to depolarize the membrane of *E. coli* JW5503 Δ*tolC* (EC_50_ of 1.0 μg ml^−1^), whereas the EC_50_ of the positive control CCCP was comparable between all three strains (Fig. 2D). In a time-dependent experiment conducted with *S. aureus* N315 (MRSA; MIC = 4 μg ml^−1^), a strong membrane depolarizing effect of 1 occurred already within the first 10 minutes after treatment with 0.5 × MIC (2 μg ml^−1^) and 2 × MIC (8 μg ml^−1^) (Fig. 2F). The depolarization effect of 1 was as fast as the one induced by the positive control CCCP. *E. coli* JW5503 Δ*tolC* also showed a membrane depolarizing effect of 1 after treatment with 0.5 × MIC (1 μg ml^−1^) and 2× MIC (4 μg ml^−1^) (Fig. 2E). However, the effect was more gradual and weaker compared to the Gram-positive strains. The differences in depolarization efficiency were larger than differences in MICs, which were only 3- and 8-fold higher for *E. coli* JW5503 Δ*tolC*. This demonstrates that the link between (rapid) depolarization and (long term) growth inhibition is not identical in Gram-positive and Gram-negative strains, but it is species-dependent, reflected in non-constant EC_50_/MIC ratios.

In order to investigate whether a depolarization only occurs across bacterial membranes, or might also be operative across host cell membranes, the integrity of the inner mitochondrial membrane of human HeLa cells (ATCC CCL-2) was probed in the presence of 1 by following the ratio of orange to green fluorescence of the MITO-ID® dye. This dye exhibits green fluorescence in the cytosol and forms orange fluorescent aggregates in the mitochondria of living cells, but the orange fluorescence is lost when the mitochondrial membranes are compromised.^20^ In this assay, the positive control CCCP lowered the membrane potential to 50% of the original value within 50 s at a concentration of 1 μM, while the DMSO control had no effect (Fig. 2G). We observed that 1 induced a *t*_1/2_ of 240 s, which clearly demonstrates the ability of the compound to impair the membrane potential of mitochondria. Loss of inner mitochondrial membrane potential can be caused by uncouplers of oxidative phosphorylation (*e.g.* protonophores such as CCCP), but it can also result from inhibition of respiratory chain proteins, as found for the complex I inhibitor rotenone,^21^ the complex III inhibitor antimycin A^22,23^ or the ATP synthase inhibitor oligomycin A.^24^ In order to probe whether 1 functioned as a respiratory inhibitor, its effect on the oxygen consumption of HeLa cells was studied. Treatment with 1 at a concentration of 10 μM led to an increase in oxygen consumption, resulting in less oxygen in the growth medium, at a rate that was equal or higher than that of the uncoupler CCCP (Fig. 2H). In contrast, the respiratory chain inhibitors antimycin A, rotenone and oligomycin A induced a decrease in oxygen consumption compared to the DMSO control. When treated with protonophores, the link between electron transport and ATP production is destroyed, and cells respond by increasing the flow of electrons through the electron transport chain, which results in an increase in oxygen consumption.^25^ Thus, the effects of 1 are consistent with the activity of an uncoupler of oxidative phosphorylation.

**Fig. 3:**
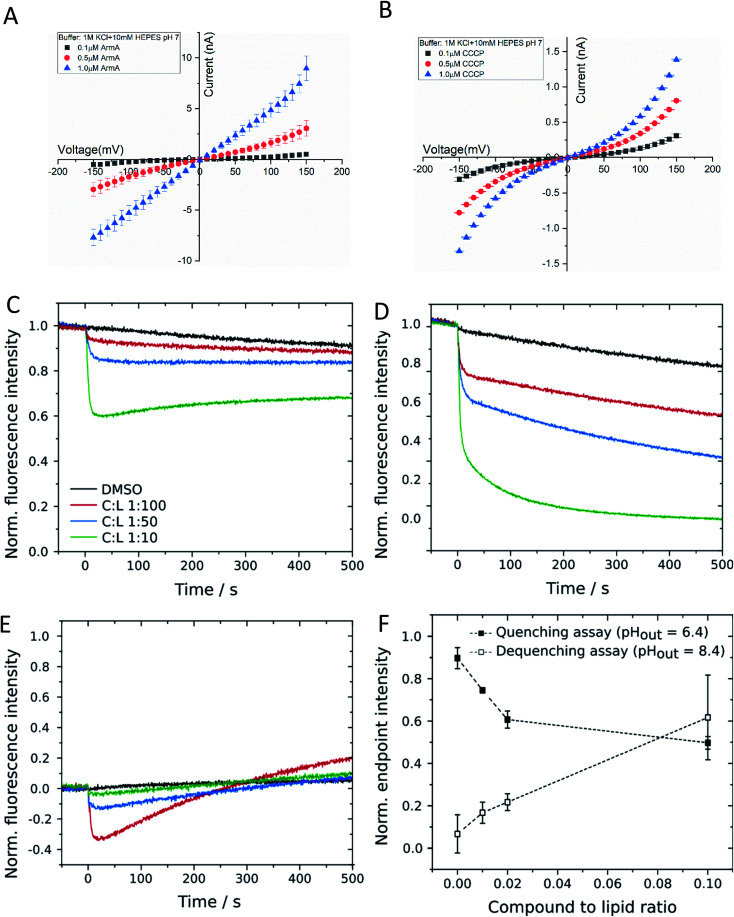
1 acts as a protonophore in a concentration dependent manner. (A and B) Ion conductance across a planar lipid bilayer. (A). Addition of 1 (=ArmA) on both sides of the membrane increased the conductivity in a concentration dependent manner. In absence of 1 the membrane conductance is negligible (data not shown). (B). For comparison we added in a separate experiment CCCP, a well characterized ionophore. In both cases the buffer contained 1 M KCl 10 mM HEPES pH 7. The measurements were averaged at least over 3 independent experiments. (C–F) Interaction of 1 with large unilamellar vesicles. (C–E) Changes in normalized pyranine fluorescence intensity upon addition of 0.5 μM to 5 μM armeniaspirol A (C : L 1 : 100 to C : L 1 : 10) without a pH gradient (pH_out_ = pH_out_ = 7.4 (C)) and with a pH gradient (pH_out_ = 6.4 (D) or 8.4 (E)). (F) Corrected normalized endpoint intensities after 500 s as a function of compound concentration for both pH assays. Vesicles were composed of POPC and filled with 100 mM KCl, 10 mM HEPES, 0.5 mM pyranine, pH 7.4 and diluted in buffer with pH 7.4 or 6.4, respectively. C : L = compound to lipid ratio.

In order to probe whether 1 is capable of translocating ions in a protein-independent manner, the membrane conductance of a planar lipid bilayer was measured. The bilayer consisted of 1,2-diphytanoyl-*sn-glycero*-3-phosphocholine (DPhPC) that was positioned across a 100 μm diameter orifice in a Teflon chamber consisting of two compartments filled with a 1 M KCl solution at pH 7.0 (ESI Fig. S3†). After applying varying voltages in the range between −100 and +100 mV, the flow of ions across the membrane was detected as an electric current. We observed that 1 induced the ion current across an artificial lipid bilayer in a concentration and voltage dependent manner (Fig. 3A). For comparison, we repeated the conductance measurement with the well-studied ionophore CCCP, and the results (Fig. 3B) agreed well with published values.^26,27^ In contrast a reduction of the bulk ion concentration from 1 to 0.1 M KCl did not change the conductance, whereas lowering the pH to 5 increased it by a factor of two, showing a proton concentration dependence as expected for protonophores (see ESI Fig. S3† for details). This finding demonstrated that 1 acted as an ion carrier across a lipid bilayer in a protein-free environment.

To further corroborate this finding, we applied a second, orthogonal method for the detection of proton transport across protein-free lipid membranes that was based on large unilamellar vesicles (LUVs) composed of 1-palmitoyl-2-oleoyl-*sn-glycero*-3-phosphocholine (POPC). First, we investigated the influence of 1 on the membrane integrity by carboxyfluorescein encapsulated in lipid vesicles in a self-quenching concentration of 100 mM. Leakage of the dye would result in an increase in fluorescence intensity due to dilution in the extravesicular medium. Even for high concentrations of 1 of 5 μM, corresponding to a compound-to-lipid ratio (C : L) of 1 : 10, no significant leakage was observed, which suggests that 1 did not destabilize vesicular membranes by the formation of large pores or lesions (ESI Fig. S4†). To assess the potential proton transport induced by 1 we used LUVs filled with the pH-sensitive dye pyranine.^28^ Before applying a pH gradient across the lipid bilayer, we checked for an interaction between 1 and fluorophore molecules by monitoring pyranine fluorescence in the absence of any pH gradient (pH_in_ = pH_out_ = 7.4). The addition of 1 to the vesicle suspension led to a concentration-dependent decrease in fluorescence intensity, which then remained nearly constant during the experiment (Fig. 3C). We checked for a specific interaction between 1 and pyranine and used other pH-sensitive dyes like carboxyfluorescein or OregonGreen 488, but independent of the chosen pH sensor, 1 induced fluorescence quenching in all cases. Since the quenching was not visible in a pure pyranine solution without lipid vesicles, it is likely that it is caused by an interaction between 1 and fluorophore molecules located at the membrane–lumen interface. Even though this finding complicated quantitative analyses, we recorded changes in pyranine fluorescence upon addition of 1 to vesicles exposed to a pH gradient. In the quenching assay (pH_out_ = 6.4), 1 caused a rapid decrease in fluorescence intensity, which was stronger compared to that found in the experiment without a pH gradient (Fig. 3D). This is indicative of a proton influx induced by 1, which evoked protonation of pyranine molecules inside the vesicles and subsequently quenching of fluorescence. Correspondingly, changes in pyranine fluorescence in case of the dequenching assay (pH_out_ = 8.4) were a convolution of the fluorophore–compound interaction, leading to the initial decrease in fluorescence intensity, and the subsequent proton efflux mediated by 1 (Fig. 3E). Proton efflux led to the deprotonation of pyranine molecules and consecutively to fluorescence dequenching. To confirm the concentration dependency of proton translocation, we determined the endpoint fluorescence intensity after 500 s for all three experiments and subtracted the initial quenching from the values obtained in the presence of a pH gradient (Fig. 3F). With this correction, a proton translocation activity of 1 is evident. We also employed the derivatives 13 and 15 in the same pH assays. Both substances demonstrated a reduced proton transport capability, but the effect was stronger for 15 than for 13 (ESI Fig. S4†). The findings confirmed that 1 acted as a protonophore in a protein-independent manner, and that both the 5-keto and the free hydroxy group are important for this activity, in line with the antibacterial activities of the compounds (ESI Table S1†).

In a previous report, 1–3 exerted no cytotoxicity in a cellular assay with the human hepatocyte cell line HepG2 at concentrations up to 30 μM.^7^ Given the effects on bacterial and mammalian cells observed in this study, we re-investigated mammalian cytotoxicity with additional cell lines and readouts (see ESI Table S4†). The viability of human T-lymphoblast Jurkat cells was quantified by flow cytometry through counting live cells labeled with the membrane-permeable SYTO 9 dye *versus* dead cells labeled with ethidium propionate. Jurkat cells, which grow in suspension, were applied for these analyses, as the use of adherent cells could lead to damage of the membranes during cell resuspension for the measurements. The treatment with 1 led to a drop in viability by 13% at 10 μM and by 62% at 100 μM. The effects were comparable to the positive controls CCCP (−64%) and hydrogen peroxide (−53%) at 100 μM (ESI Fig. S6†). Complementary assays were conducted with five adherent cell lines. For the murine fibroblasts L929 and the human alveolar-basal epithelial cells A549, metabolic activity was measured by monitoring by the reduction of resazurin by NAD(P)H-dependent oxidoreductases, which in turn is a measure for viability. Here, treatment with 1 led to IC_50_ values of 9.2 μM (for L929) and 11.5 μM (for A549; ESI Table S4†). To determine the metabolic activity of HeLa, HUVEC and KB3.1 cells, the 3-(4,5-dimethylthiazol-2-yl)-2,5-diphenyltetrazolium bromide (MTT) assay was used, which showed an even stronger effect of 1 with IC_50_ values of 3.1 μM, 2.1 μM and 3.1 μM, respectively. The IC_50_ of L929 cells was determined to be 6 μM, which was slightly lower, but still comparable to the value for the resazurin reduction. To probe whether 5 exerts immediate toxic effects, the ATP content was quantified after 1 h and 3 h in L929 cells. An acute, concentration-dependent toxicity became visible already after 1 h with an IC_50_ at *ca.* 200–300 μM (ESI Fig. S5†). The rapid effects are in line with the proposed mechanism of action. Thus, we conclude that the depolarization of mitochondrial membranes induced by 1 might translate to impaired cellular viability; this effect appears stronger in models with adherent cell lines. In fact, the first report on the promising antibiotic efficacy of 1 in a mouse model noted that ‘at higher doses, adverse cardiac side-effects were observed’.^7^ The current mechanistic findings provide a mechanistic rationale for this dose-limiting toxicity.

Our proposal of a membrane-mediated, protein-independent antibacterial mechanism of action is supported by the fact that the separated enantiomers of the related chloro-armeniaspirol 4 had an equipotent antibacterial activity against three Gram-positive strains;^10^ in addition, enantiopure 1 was as active as racemic 5 against *M. luteus*. Such effects would not be expected for compounds acting on a chiral protein environment, but are in line with proton transporters across lipid membranes. The observation that the antibiotic activity of 1 is completely lost upon the alkylation of the phenol, the only acidic proton in the molecule, is also compliant with the mechanism. Moreover, the inability to raise resistant mutants of *S. aureus* against armeniaspirols through serial passaging is in line with the mechanism, as a bacterial defense against protonophores through single point mutations is hardly conceivable.

In a recently published study, Labana *et al.* (2021) observed that the treatment of *Bacillus subtilis* with 4 does not induce a proteomic signature that matches common antibiotics mechanisms like inhibition of protein synthesis, fatty acid biosynthesis, cell wall biosynthesis, or DNA damaging.^9^ Furthermore, the authors applied a chemoproteomic approach to discover several binding partners of a covalent capture probe derived from 4, and they report that 4 inhibited the protease and ATPase activities of ClpYQ and ClpXP complexes with IC_50_ values between 15 and 42 μM, and ATP hydrolysis was impaired at IC_50_ values between 42 and 108 μM. These enzymes are components of the bacterial divisome, and Labana *et al.* also show microscopic images of an abnormal distribution of these proteins within the cells upon incubation with 4, combined with dysfunctional cell division and deduce that the bacterial divisome is impaired. In our hands, the natural armeniaspirol 2 exhibited moderate inhibition of ClpP and weak inhibition of ClpXP, reducing the peptide and protease activities of ClpP and ClpXP by 73% and 33%, respectively, at a concentration of 100 μM (ESI Fig. S7†). In line with the Labana study, a covalent modification of ClpP by 2 was not observed (ESI Fig. S8†). Because the inhibitory concentrations are much higher than the MICs, we argue that membrane depolarization is a strong and probably the primary contributor to the antibiotic effect at lower armeniaspirol concentrations. We also note that the low frequency of resistance observed for armeniaspirols contrasts the high frequencies of resistance found for some ClpP binders. However, the existence of a secondary mechanism of action, and in particular the combined targeting of enzymes and the proton motive force, is not unusual for antibiotics,^29^ and it constitutes a clear advantage in terms of impeding bacterial resistance development. The protonophoric activity of armeniaspirols might also contribute to the inhibition of the divisome reported by Labana *et al.*: for example, indole was observed to prevent cell division by altering the membrane potential in *E. coli*, and CCCP addition led to a dyslocalization of key proteins involved in cell division in *Bacillus subtilis*.^30–32^

**Fig. 4:**
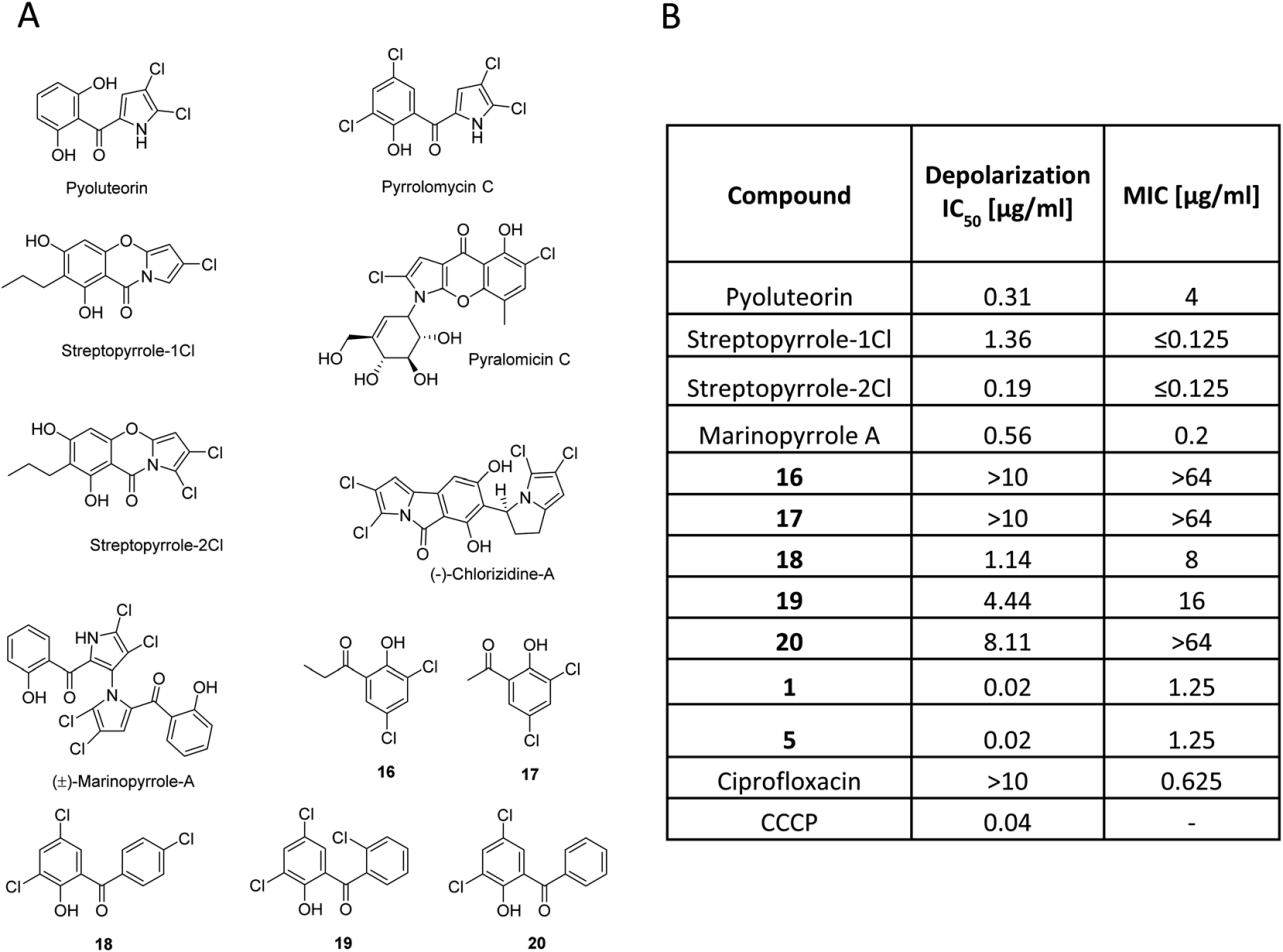
Studies on chloropyrrole-containing natural products and synthetic analogs. (A) Structures. (B) Bioactivities on *Micrococcus luteus* DSM1790. EC_50_ values were obtained in a membrane depolarization assay using DiOC_2_ (3) (Baclight™ assay), and minimal inhibitory concentration (MIC) values were obtained by the microbroth dilution method.

Armeniaspirols are part of a larger family of natural products that all carry chlorinated pyrrole moieties. The compounds have been isolated from diverse origins, as exemplified by pyoluteorin, a widely studied signaling molecule from Gram-negative *Pseudomonas* sp.,^33^ pyralomicin from *Nonomuraea spiralis*,^34^ pyrrolomycin and streptopyrrole from terrestrial *Streptomyces* sp.,^35,36^ or chlorizidine^37^ and marinopyrrole A^38^ from marine *Streptomyces* sp. Their individual scaffolds and the underlying biosynthesis are different (Fig. 4), however, they do not only share a chloro-pyrrole as a common feature, but also an acylated phenol moiety. Because many of them exhibit profound antibiotic activities, we hypothesized that this might be traced back to a common mechanism as a protonophore. In fact, their calculated p*K*_a_ values were in a range of 5.95 to 8.8 (ESI Table S5†), which is in line with a protonophore activity. The p*K*_a_ value determined for 1 was 8.28, which is comparable to streptopyrrole-1Cl and for the reported protonophores CCCP and pyrrolomycin C they are 6.04 and 5.95, respectively. We next tested the membrane depolarization capabilities of marinopyrrole, the streptopyrroles 1Cl and 2Cl and pyoluteorin, the subset of natural products that was accessible to us, and observed that they indeed induced polarization at EC_50_ values of 0.56, 1.36, 0.19 and 0.31 μg ml^−1^, respectively (Fig. 4). However, all natural products did not reach the high potency (EC_50_ = 0.02 μg ml^−1^) of 1. The findings are fully in line with a recent study of Valderrama *et al.*, who reported that pyrrolomycins are potent protonophores.^39^ In parallel, a set of synthetic compounds that also had an acyl phenol moiety, but lacked the chloropyrrole ring was investigated as a control in depolarization and in antibacterial assays. The simple synthetic acylphenols 16 and 17 were inactive, but synthetic pyrrolomycin analogs 18–20 carrying a phenyl-linked acylphenol were active. Albeit the test series contained non-perfectly matched molecular pairs, trends indicate that an increased number of chlorine atoms (compare streptopyrrole-2Cl *vs.* streptopyrrole-1Cl; 18 and 19*vs.*20) and that a pyrrole ring rather than a phenyl ring are beneficial for the bioactivity. The antibacterial activity of the compounds against *M. luteus* correlated directly with the strength of membrane depolarization (Fig. 4B). This suggests that depolarization might be a main contributor to the antibacterial effect. We derive the following, yet preliminary conclusions from this dataset: (i) the antibiotic properties exerted by many natural products containing chloropyrrole and an acyl-phenol moiety might jointly stem from a membrane depolarization activity. (ii) Future studies need to clarify in how far the chloropyrrole moiety employed by nature is particularly advantageous for migrating through lipid membranes.

It was previously reported that resistance development in *S. aureus* was not observed.^7,40^ Therefore, we selected the Gram-negative *E. coli* K12 Δ*tolC* strain to raise resistant mutants against 1 and 2. Indeed, we were able to isolate armeniaspirol-resistant mutants that developed at a frequency of 2.5 × 10^−8^ for 1 and 1.25 × 10^−8^ for 2, and displayed a shift in MIC of >32-fold compared to the parent strain (ESI Table S2†). Whole-genome sequencing revealed that 46% of the mutants resistant against 1 had mutations within *mdtO* (encoding a component of an RND efflux system) and *prfB* (encoding peptide chain release factor 2), while 54% of mutants showed a single point mutation in the intergenic region downstream of the *csrA* gene and upstream from the tRNA-ser gene. Further, we observed single point mutations in armeniaspirol B (2) resistant clones in the intergenic region upstream of *cvpA* (encoding colicin V production accessory protein) and *fdoG* (encoding formate dehydrogenase-O major subunit), and another clone showed mutations in *plsB* (encoding membrane-bound glycerol-3-phosphate acyltransferase) (ESI Table S3†).

As stated, armeniaspirols belong to a large family of natural products that all contain chlorinated pyrrole moieties, which share a proposed common mechanism of action. We reasoned that also their mechanism of resistance might be similar, and we therefore assessed the MICs of 1 and 2 against a pyrrolomycin-resistant *E. coli* Δ*tolC* mutant (Δ*tolC* pyrr RC1) described by Valderrama *et al.* (Table 3).^39^ The pyrrolomycin-resistant mutants showed a 8855-bp deletion of nine transcribed genes, namely *alsR*, *alsB*, *alsA*, *alsC*, *alsE*, *alsK*, *yjcS*, *ytcA*, and *mdtN* (accession no. CP009273.1). It was proposed that the deletion of these genes allowed *mdtOP* to be in close proximity to the *als* operon promoter (P_*als*_), which allowed for the expression of *mdtOP via* P_*als*_ derepression as a result of the disruption of the transcriptional repressor AlsR.^39^ The *mdtO* and *mdtP* genes are homologous of *acrB* and *tolC*, respectively, and thus, their products could possibly compensate the loss of the TolC.^41^

We found that the pyrrolomycin-resistant mutant (*E. coli* Δ*tolC* pyrr RC1) as well as the engineered validation strain EP676, both carrying the 8855-bp deletion of nine genes, were fully cross-resistant with armeniaspirols (MICs > 64 μg ml^−1^), which confirms the role of the MdtNOP efflux system in armeniaspirol resistance of TolC-deficient *E. coli*, as described for pyrrolomycin (Table 3).^39^ Furthermore, the EP680 strain with the additional deletion of the *mdtO* and *mdtP* genes alongside the nine other genes (*alsR*, *alsB*, *alsA*, *alsC*, *alsE*, *alsK*, *yjcS*, *ytcA*, and *mdtN*) showed sensitivity to armeniaspirols, indicating the importance of the *mdtO* and *mdtP* genes in resistance to armeniaspirols. The collected resistant mutant data, MIC data and the proposed common mechanism of action of natural products containing chlorinated pyrrole moieties provide strong evidence for an efflux-mediated mechanism of resistance in Gram-negative bacteria.

Further investigation is still ongoing to describe the resistance mechanism linked to the single nucleotide mutation in the intergenic region downstream of the *csrA* gene and upstream of the gene for tRNA-Ser, which was present in 54% of the mutants. However, the observed single point mutations for 2 in the intergenic region upstream of *cvpA* and *fdoG* as well as *plsB* can all be linked to the stringent response, a stress response pathway that promotes membrane potential homeostasis (see the ESI†).^42^

## Conclusion

In this study, we report the first total synthesis of armeniaspirol A (1) that forms the spirocycle through a metal-catalyzed radical cross-coupling reaction in the key step. With five steps from commercial starting material, the synthesis enables a systematic exploration of structure–activity relationships of armeniaspirols. Moreover, we found that 1 leads to membrane depolarization at concentrations that are in the same range as the antibacterial activities, and we provide evidence that 1 acts as a protonophore across membranes in a protein-independent manner. This represents an original, so far rarely explored (but not unprecedented^39,43^) mechanism for natural antibiotics, and it could be operative in the larger family of natural products carrying chloropyrrols coupled to an acyl-phenol motif. On the other hand, the mechanism is also operative in mammalian cells, and assuring selectivity of antibacterial activity over mammalian cell toxicity will be an important and challenging goal of future optimization programs. Further, we analyzed the mechanism of resistance leading us to identify an efflux mediated and possibly an additional efflux-independent mechanism for Gram-negative *E. coli* Δ*tolC*.

## Funding

This work was co-funded by the German Centre for infection research (Grant no. TTU09.710), the Helmholtz International Lab for Anti-Infectives, and the President's Initiative and Network Fund of the Helmholtz Association of German Research Centres (HGF) under contract number VH-GS-202.

## Data availability

All experimental data is available in the ESI.†

## Author contributions

NA planned and performed all synthetic work. HLSF planned and performed experiments, analysed the data and contributed to writing the manuscript. MO, BH, JC and JH performed mechanistic experiments and analyzed the data. CS designed part of the experiments. DR designed and performed part of the experiments. KE performed ClpP inhibition assays. AB provided compounds and analyzed the data. DH calculated properties and analyzed the data. EK re-isolated armeniaspirols and performed mechanistic experiments. SPB, JAB and MW designed and SPB and JAB performed the planar lipid bilayer experiments. RM and SAS designed studies and analyzed the data. PPM designed and coordinated biological studies that revealed the protonophore mechanism of action. MB conceived the study, analyzed the data, and wrote the manuscript. All authors read and approved the final version of the manuscript.

## Conflicts of interest

The authors declare no conflict of interest.

## Supplementary Material

SC-012-D1SC04290D-s001
