# Supplementary material for: Total synthesis and mechanism of action of the antibiotic armeniaspirol A
Source: Chem Sci. 2021 Nov 24;12(48):16023–34. doi: 10.1039/d1sc04290d (PMC8672772; doi:10.1039/d1sc04290d)
Supplement: SC-012-D1SC04290D-s001 [file SC-012-D1SC04290D-s001.pdf]

Electronic Supplementary Material (ESI) for Chemical Science.
This journal is © The Royal Society of Chemistry 2021

Supporting Information: Total Synthesis and Mechanism of Action of the Antibiotic Armeniaspirol A

Nanaji Arisetti, Hazel Leanne Sarah Fuchs, Janetta Coetzee, Manuel Orozco, Dominik Ruppelt, Armin Bauer, Dominik Heimann, Eric Kuhnert, Satya Prathyusha Bhamidimarri, Jayesh Arun Bafna, Bettina Hinkelmann, Konstantin Eckel, Stephan A. Sieber, Peter Paul Müller, Jennifer Herrmann, Rolf Müller, Mathias Winterhalter, Claudia Steinem, Mark Brönstrup

Table of Content

Experimental Procedures: Armeniaspirol synthesis 3

General Procedures 3

Natural armeniaspirols A (1) and B (2) 3

Unsuccessful attempts to synthesize armeniaspirol A 4

Supplementary Scheme S1. 4

Synthesis of (±) armeniaspirol A (5) and derivatives 13 and 15 8

Supplementary Scheme S2. Mechanism of radical-induced addition of phenols to alkenes. 12

NMR spectra 13

Experimental Procedures: Antibacterial efficacy, cytotoxicity, mode of action and mode of resistance 36

Antibacterial susceptibility testing in 384 well MTP format 36

Supplementary Table S1: Growth inhibitory activities of armeniaspirols on *M. luteus* DSM1790 and *S. aureus* DSM346. 37

Antibacterial susceptibility testing of *E. coli* strains in 96 well MTP format 37

Effect of pH on antibacterial activity of *Micrococcus luteus* in 384 well MTP format 38

Supplementary Figure S1: pH dependency of antibacterial activity of 1 on *M. luteus* DSM1790. 39

Membrane depolarization assays 40

Supplementary Figure S2: Comparison of membrane depolarising effect of 1 and 5. 41

Effects on electron transport chain 42

Planar lipid bilayer assays 43

Supplementary Figure S3: Planar lipid bilayer assays. 44

Vesicular lipid bilayers 45

Supplementary Figure S4: Experiments with vesicular lipid bilayers. 47

In vitro resistance development, frequency of resistance, and resistance level of mutants 48

Supplementary Table S2: MICs of armeniaspirol A (1) and armeniaspirol B (2) obtained from wild-type and resistant mutants of the *E. coli* K12 Δ*tolC* strain. 48

Whole-Genome Sequencing 49

Supplementary Table S3: List of mutations obtained from spontaneous resistant development of *E. coli* K12 Δ*tolC* strain. Mapped to CP018801.[7] 49

Relevance of observed mutations 50

Cytotoxicity testing 51

Supplementary Table S4: Cytotoxic activity of 1 and CCCP on selected murine and human cell lines. 52

Supplementary Figure S5: Acute cytotoxicity of 5, as assessed by cellular ATP content. L929 cells (53

Supplementary Figure S6: Cytotoxicity of 1 in Jurkat cells. 54

Experiments with *E. coli* ClpP and ClpXP 55

Supplementary Figure S7: *E. coli* ClpP peptidase (A) and ClpXP protease (B) assays. 55

Supplementary Figure S8: Intact protein mass spectra of *E. coli* ClpP 55

Calculation of pK_a values and other physicochemical properties of chloropyrrole-containing natural products 58

Supplementary Table S5: Structures, calculated pK_a values and other physicochemical properties of chloropyrrole-containing natural products. The colors indicate the deprotonation sites. 59

References 61

Experimental Procedures: Armeniaspirol synthesis

General Procedures

All non-aqueous reactions were carried out under argon atmosphere in flame-dried glassware with a rubber septum in dry solvents unless otherwise noted. Tetrahydrofuran (THF), dichloromethane (DCM), trifluoroacetic acid (TFA), acetonitrile (ACN) were dried by passing through activated alumina. Unless otherwise stated, all chemicals were used as received. All reactions were monitored by thin-layer chromatography using Merck silica gel 60 F_{254} precoated plates and were visualized by UV (254 nm), CAN and/or $KMnO_4$ staining. Flash chromatography was performed using silica gel 60 (0.063-0.2 mm) purchased from Macherey-Nagel (MN). ^{1}H, ^{13}C and 2D-NMR spectra were recorded on a 500 MHz Avance III (UltraShield Plus, Bruker) spectrometer, equipped with a 5mm RT BBO probe or on a 700 MHz Avance III HD (Ascend, Bruker) spectrometer, equipped with a 5mm TCI cryoprobe and referenced to $CDCl_3$ signals at 7.26 and 77.0 ppm, unless otherwise noted. The data is reported as (s= singlet, d= doublet, t= triples, q= quartet, m=multiplet, dd= doublet of doublet).

High resolution mass spectrometry (HRMS) data were recorded using a Dionex Ultimate 3000 HPLC system equipped with a DAD detector and a maXis HD QTOF mass detector (Bruker Daltonics, Bremen, Germany) with electrospray ionization (ESI).

Abbreviations: THF= tetrahydrofuran, DCM= dichloromethane, ACN= acetonitrile, TFA= trifluroacetic acid, DDQ= 2,3-Dichloro-5,6-dicyano-1,4-benzoquinone, n-BuLi= n-butyllithium

Natural armeniaspirols A (1) and B (2)

Natural armeniaspirol A (**1**) and B (**2**) were obtained by fermentation and isolation according to described procedures.[1, 2]

Unsuccessful attempts to synthesize armeniaspirol A

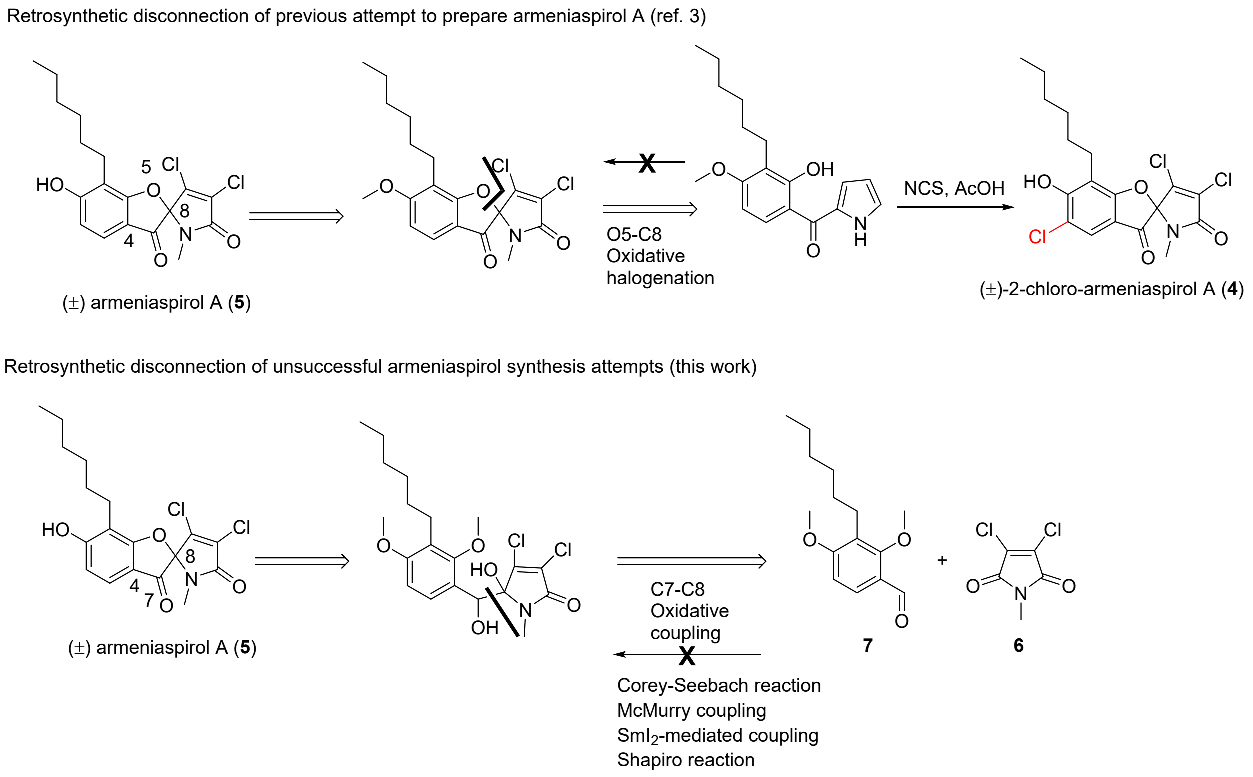

Supplementary Scheme S1. Retrosynthetic disconnections followed in previous attempts (top; ref 3) and this work (bottom) to synthesize armeniaspirol A

Umpolung strategy

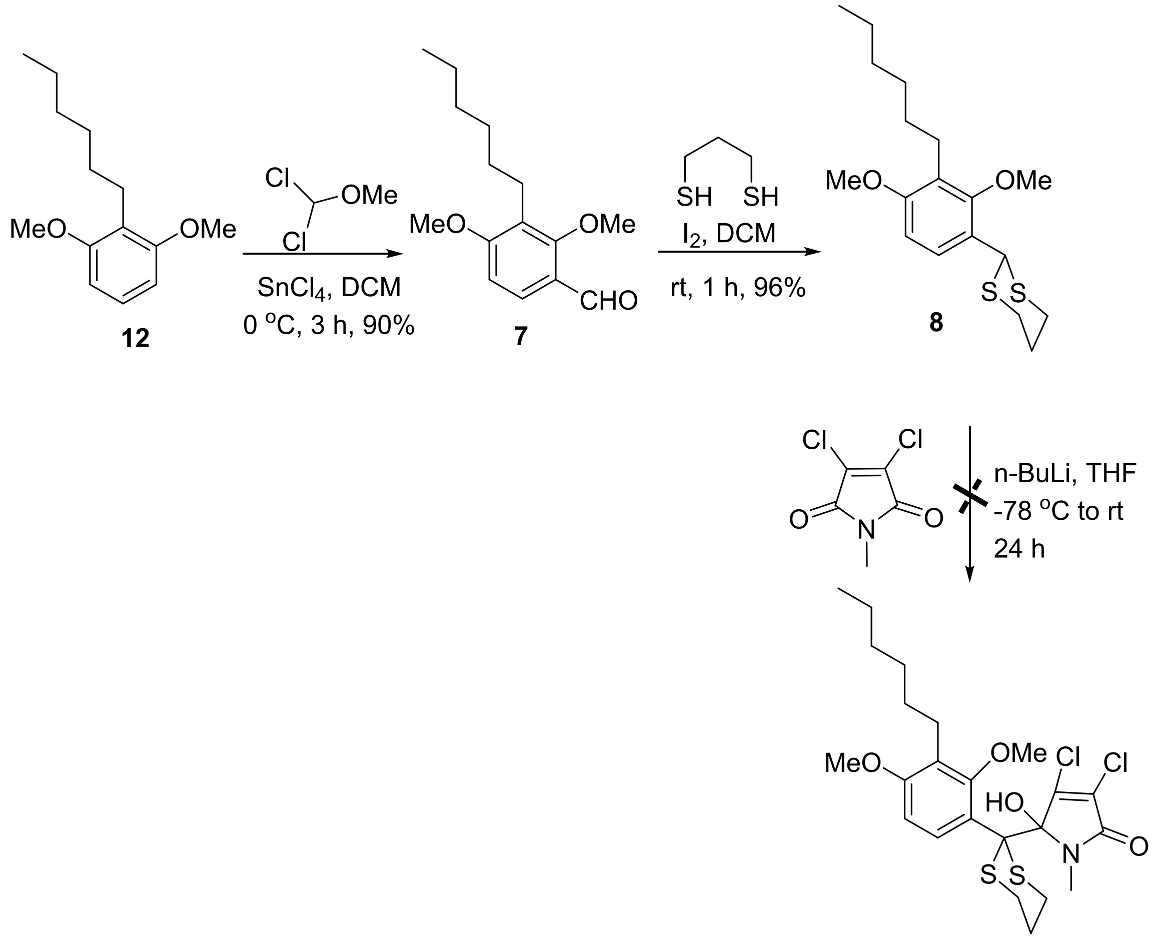

To a solution of **7** (0.222 g, 1 mmol, 1 equiv.) in DCM (4 mL) was added $SnCl_4$ (0.15 mL, 1.3 mmol, 1.3 equiv.) at 0 oC and stirred for 1h at the same temperature. Then dichloromethoxymethane (0.11 mL, 1.2 mmol, 1.2 equiv.) was added and stirred for at the same temperature for another 1h. The reaction mixture was poured into cold water (5 mL) and stirred for 1h at RT. Then the mixture was extracted with DCM (10 mL). The resulting organic layers were washed with brine and dried. Concentrated under *vacuo* and crude was purified by flash column chromatography (PE: EA= 5:1) resulted the desired aldehyde **11** (0.225 g, 0.9 mmol, 90%) pale yellow oil.

^{1}H NMR: (500 MHz, $CDCl_3$) δ 10.21 (s, 1H), 7.74 (t, *J* = 6.8 Hz, 1H), 6.75 (d, *J* = 8.7 Hz, 1H), 3.89 (d, *J* = 5.8 Hz, 6H), 2.63 (dd, *J* = 8.9, 6.9 Hz, 2H), 1.59 – 1.50 (m, 2H), 1.42 – 1.31 (m, 6H), 0.90 (dd, *J* = 9.1, 4.9 Hz, 3H).

^{13}C NMR: (126 MHz, $CDCl_3$) δ 189.15, 163.91, 162.43, 128.52, 125.25, 122.84, 106.80, 64.25, 55.81, 31.62, 29.53, 23.35, 22.58, 14.06.

To a solution of **11** (0.175 g, 0.7 mmol, 1.0 equiv) in DCM (4 mL) was added I_2 (0.018 g, 0.07 mmol, 0.1 equiv.) and 1,3-propanedithiol (0.078 mL, 0.77 mmol, 1.1 equiv) and stirred for 1h at RT. The reaction mixture was quenched with 0.1 M $Na_2S_2O_3$ solution and extracted with DCM (5 mL). The resulting organic layers were washed with brine, dried and concentrated under *vacuo*. The crude was used for the next step without any purifications.

^{1}H NMR: (500 MHz, $CDCl_3$) δ 7.43 – 7.40 (m, 1H), 6.67 (d, *J* = 8.6 Hz, 1H), 3.85 (s, 3H), 3.81 (s, 3H), 3.12 (ddd, *J* = 14.3, 10.2, 8.5 Hz, 2H), 2.90 (dt, *J* = 7.2, 4.0 Hz, 2H), 2.61 (dd, *J* = 9.0, 6.8 Hz, 2H), 2.18 (dtd, *J* = 14.1, 4.3, 2.3 Hz, 1H), 2.01 – 1.90 (m, 1H), 1.52 (dtd, *J* = 9.7, 7.0, 4.3 Hz, 2H), 1.39 – 1.30 (m, 6H), 0.91 – 0.88 (m, 3H).

^{13}C NMR: (126 MHz, $CDCl_3$) δ 158.63, 155.56, 127.04, 124.92, 124.63, 106.95, 62.68, 55.54, 44.53, 32.64, 31.67, 29.75, 29.67, 25.24, 24.24, 22.62, 14.08.

<u>Strategy based on the Shapiro reaction</u>

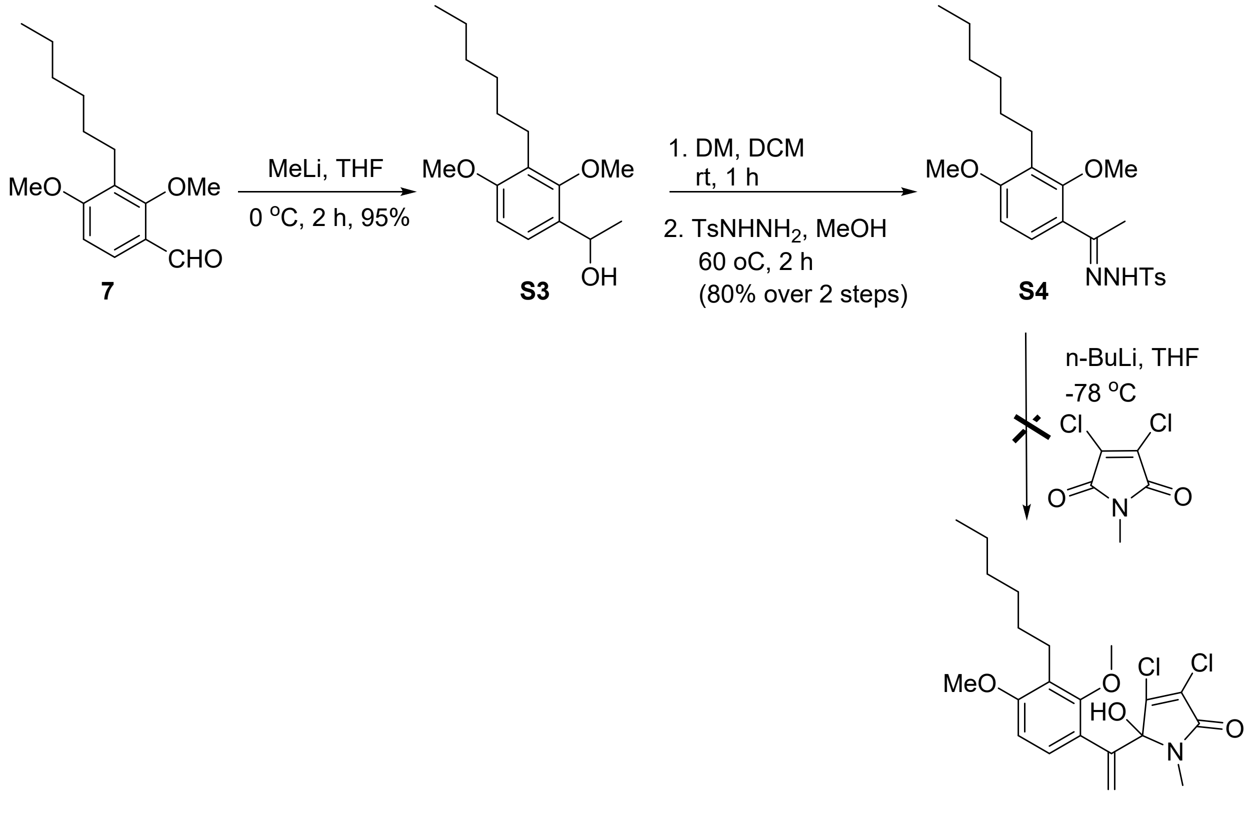

To a solution of **7** (0.25 g, 1 mmol, 1 equiv) in THF (2.5 mL) was added MeLi (1.6 M, 0.9 mL, 1.5 mmol, 1.5 equiv) at 0 °C and stirred for 2h at the same temperature. The mixture was quenched with aq. NH_4Cl (2 mL) and extracted with Et_2O (5 mL). The resulting organic layers were washed with brine, dried and concentrated under *vacuo* gives crude (**S3**), which was used directly for the next step.

To the above crude in DCM (4 mL) was added Dess-Martin reagent (0.63 g, 1.55 mmol, 1.5 equiv) and stirred for 1h at RT. The reaction was quenched with aq. $Na_2S_2O_3$ solution and extracted with DCM. The resulting organic layers were washed with brine, dried and concentrated under *vacuo* gives crude (**S4**), which was used for the next step without any further purifications.

To the above crude in MeOH (1.5 mL) was added $TosNHNH_2$ (0.186 g, 1 mmol, 1 equiv) at RT and stirred at 60 °C for 2h. The excess solvent was removed under vacuo, the resulting crude was purified by flash column chromatography (PE: EA= 5:1) gives the desired S4 (0.33 g, 0.78 mmol, 77%) as a solid.

^{1}H NMR: (500 MHz, $CDCl_3$) δ 7.90 – 7.84 (m, 1H), 7.33 (dt, *J* = 4.4, 2.2 Hz, 1H), 6.91 – 6.82 (m, 1H), 6.69 (dd, *J* = 9.3, 7.4 Hz, 1H), 3.86 – 3.81 (m, 1H), 3.23 (d, *J* = 3.0 Hz, 1H), 2.62 – 2.57 (m, 1H), 2.47 – 2.44 (m, 1H), 2.23 (s, 1H), 1.50 – 1.45 (m, 1H), 1.41 – 1.33 (m, 2H), 0.93 – 0.89 (m, 1H).

^{13}C NMR: (126 MHz, $CDCl_3$) δ 159.86, 155.19, 153.21, 143.69, 135.85, 129.52, 128.03, 125.90, 119.25, 107.07, 61.70, 55.76, 31.63, 29.64, 24.81, 23.68, 22.61, 21.57, 14.14.

Synthesis of (±) armeniaspirol A (5) and derivatives 13 and 15

Synthesis of 2-hexylresorcinol (**12**)

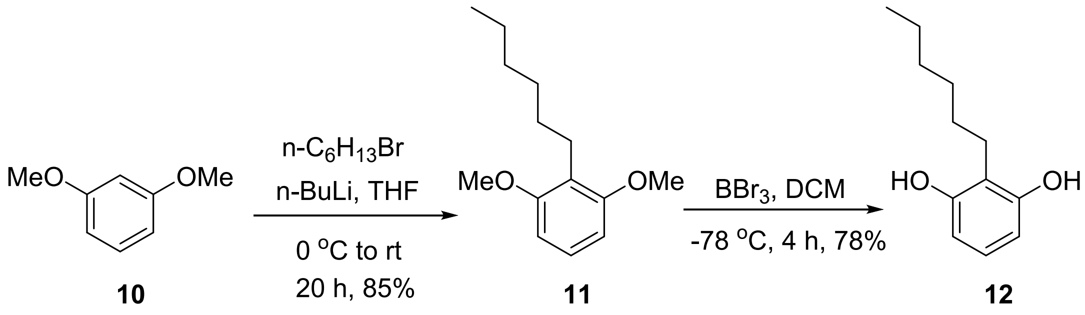

To a solution of **10** (1.3 mL, 10 mmol) in dry THF (50 mL) at 0 °C was added n-BuLi (2.5 M in hexane, 4.8 mL, 12 mmol, 1.2 equiv). The resulting solution was stirred at the same temperature for 1h and at r.t. for 2h. Again, the mixture was cooled to 0 °C then added 1-bromohexane (1. 6mL, 11 mmol, 1.1 equiv). The mixture was allowed to warm to r.t. and then stirred at the same temperature for 17h. The reaction was quenched with aq. NH_4Cl (50 mL). The aqueous phase was extracted with Et_2O (100 mL). The combined organic extracts were dried over Na_2SO_4, filtered and concentrated in *vacuo*. The crude was used for the next step without any further purification.

To a solution of **11** (4.44 g, 20 mmol) in DCM (100 mL) at -78 °C was added BBr_3 (1.0 M in DCM, 60 mL, 60 mmol, 60 equiv.) The mixture was stirred at the same temperature for 4h. Then quenched with aq. NH_4Cl (200 mL). The aqueous layer was extracted with DCM (2 x 100 mL). The resulting organic layers were washed with brine (100 mL), dried and concentrated in *vacuo*. The crude was purified by flash column chromatography (PE: EA= 5:1 to 2:1) which gives the product **12** in (3.0 g, 15.5 mmol) 78% yield as a brown solid.

^{1}H NMR: (500 MHz, $CDCl_3$) δ 6.93 (t, *J* = 8.1 Hz, 1H), 6.40 (d, *J* = 8.1 Hz, 2H), 4.74 (s, 2H), 2.68 – 2.61 (m, 2H), 1.61 – 1.54 (m, 2H), 1.41 (tt, *J* = 12.5, 6.3 Hz, 2H), 1.37 – 1.30 (m, 4H), 0.93 – 0.87 (m, 3H).

^{13}C NMR: (126 MHz, $CDCl_3$) δ 154.59, 126.73, 115.47, 107.94, 31.76, 29.43, 29.05, 23.14, 22.62, 14.09.

Synthesis of 3,4-dichloro-1-methyl-5-methylene-1,5-dihydro-2H-pyrrol-2-one (**9**)

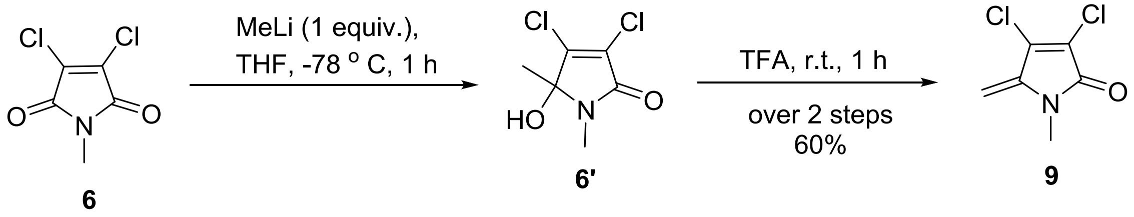

To a solution of **6** (2.63 g, 14.7 mmol) in dry THF (70 mL) was added MeLi (1.6 M in Et_2O, 9.2 mL, 14.7 mmol, 1.0 equiv) at -78 °C and stirred at the same temperature for 1h. The resulting mixture was quenched

with aqueous NH_4Cl (50 mL) and extracted with Et_2O (100 mL). The combined organic layers were washed with brine, dried and concentrated in *vacuo*. The flash column chromatography (PE: Et_2O= 1:1) furnished the desired product **6'** as a white solid, which was used for the next step without any further purifications.

TFA (61 mL) was added to **6'** (1.2 g, 6.15 mmol) and the solution was stirred for 1h at RT. The excess TFA was removed under *vacuo* and crude was purified by flash column chromatography (PE: Et_2O= 1:1) gives the desired product **9** (1.08 g, 6.15 mmol, >99%) as a pale yellow powder.

^{1}H NMR: (500 MHz, $CDCl_3$) δ 5.21 (d, *J* = 2.7 Hz, 1H), 4.99 (d, *J* = 2.7 Hz, 1H), 3.19 (s, 3H).

^{13}C NMR: (126 MHz, $CDCl_3$) δ 162.36, 142.13, 134.37, 125.37, 95.32, 26.56.

HRMS (ESI): *m/z* calculated for $C_6H_6Cl_2NO$ $[M+H]^+$: 177.9821 Da; found: 177.9820 Da.

Synthesis of 7-deoxy-armeniaspirol A (**13**)

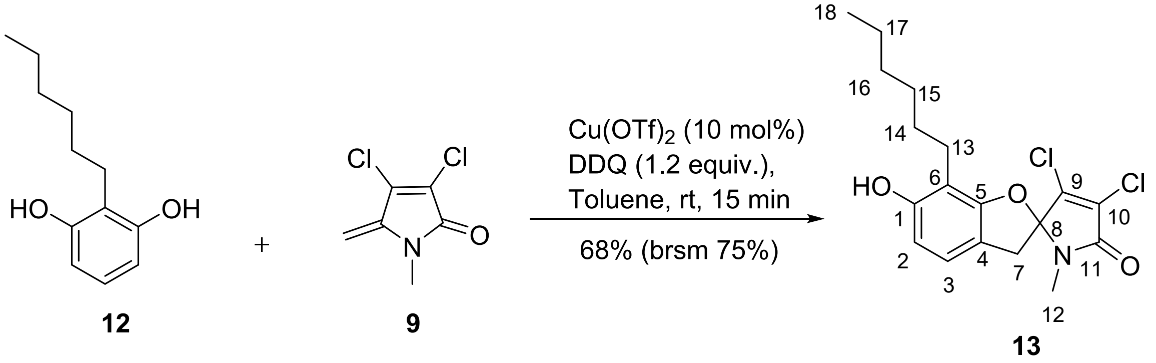

DDQ (0.032 g, 1.2 equiv) and $Cu(OTf)_2$ (0.005 g, 10 mol%) were added to an oven dried flask equipped with stir-bar. The flask the evacuated three times and purged with Argon. **12** (0.025 g, 0.13 mmol) **9** (0.023 g, 1.0 equiv) and toluene (0.5 mL) were added simultaneously. The mixture was stirred at RT for 15 min and directly purified by flash column chromatography (PE: EA= 5:1 to 2:1) give the desired product **13** (0.032 g, 0.086 mmol, 68%) as a brown solid.

^{1}H NMR: (500 MHz, $CDCl_3$) δ 6.91 (d, 1H), 6.43 (d, *J* = 8.0 Hz, 1H), 3.57 (dt, *J* = 7.6, 3.8 Hz, 1H), 3.27 (dd, 1H), 2.87 (s, 3H), 2.62 – 2.55 (m, 2H), 1.55 (dd, *J* = 14.9, 7.5 Hz, 2H), 1.33 (tt, 6H), 1.28 (m, 4H), 0.87 (ddd, *J* = 7.2, 2.5, 1.2 Hz, 3H).

^{13}C NMR: (126 MHz, $CDCl_3$) δ 162.06, 157.49, 154.45, 143.58, 126.54, 121.63, 114.97, 112.73, 108.83, 99.78, 34.75, 31.73, 29.13, 28.92, 24.82, 23.54, 22.62, 14.11.

HRMS (ESI): *m/z* calculated for $C_{18}H_{22}Cl_2NO_3$ $[M+H]^+$: 370.0971 Da; found: 370.0971 Da.

Synthesis of (±)-armeniaspirol A (**5**)

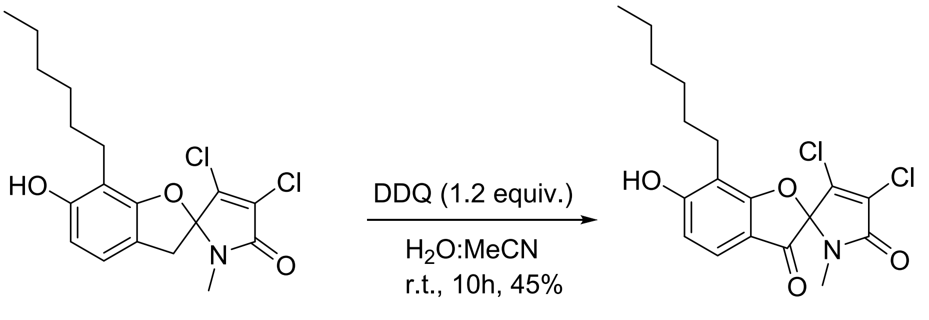

13 **5**

To a solution of **13** (0.05 g, 0.135 mmol) in 80% aq. MeCN (0.121 mL) was added DDQ (0.037 g, 0.162 mmol, 1.2 equiv). The mixture was stirred at RT for 10h. The solvent was removed under *vacuo* and the crude was directly purified by flash column chromatography (PE: EA= 10:1 to 2:1) gives the target compound **5** (0.022 g, 0.057 mmol, 45%) as pale yellow solid.

^{1}H NMR: (700 MHz, $CDCl_3$) δ 7.51 (d, *J* = 8.4 Hz, 1H), 6.76 (s, 1H), 6.73 – 6.71 (m, 1H), 2.82 (s, 3H), 2.71 (dd, *J* = 12.3, 4.8 Hz, 2H), 1.65 – 1.59 (m, 2H), 1.38 (dq, *J* = 8.0, 6.2 Hz, 2H), 1.31 (ddd, *J* = 14.4, 7.5, 2.9 Hz, 4H), 0.89 (t, *J* = 7.0 Hz, 3H).

^{13}C NMR: (126 MHz, $CDCl_3$) δ 189.18, 172.10, 164.36, 163.48, 138.84, 128.79, 124.30, 113.87, 112.92, 112.52, 97.13, 31.67, 29.12, 28.59, 25.82, 22.61, 22.49, 14.06.

HRMS (ESI): *m/z* calculated for $C_{18}H_{20}Cl_2NO_4$ $[M+H]^+$: 384.0764 Da; found: 384.0763 Da.

Synthesis of 1-methoxy-(±)-armeniaspirol A (**15**)

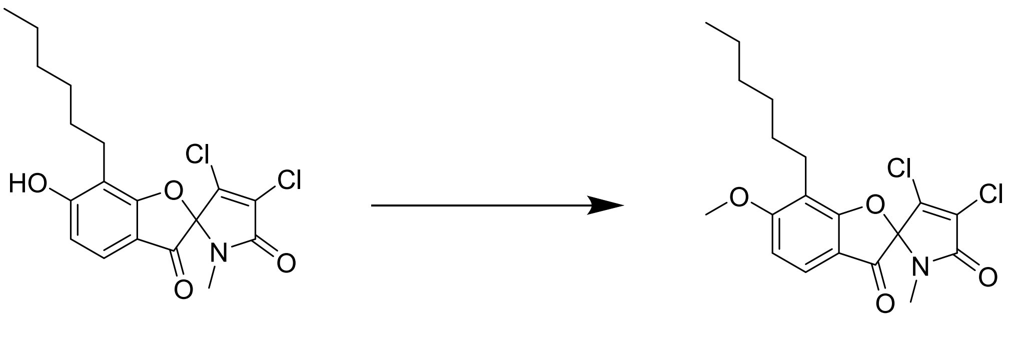

5 **15**

To a solution of 5 (0.02 g, 0.052 mmol) in dry DMF (0.5 mL) were added MeI (0.037 g, 5 equiv) and K_2CO_3 (0.014 g, 2 equiv). The mixture was stirred at rt for 24h. The reaction mixture was quenched with water (1 mL) and extracted with EtOAc (1 mL x2). The resulting organic layers were washed with brine and dried. The crude was purified by flash chromatography (PE: EA= 5:1) to give the product in 50 % yield (0.01 g) as brown oil.

^{1}H NMR: (500 MHz, $CDCl_3$) δ 7.61 (d, *J* = 8.6 Hz, 1H), 6.77 (d, *J* = 8.7 Hz, 1H), 3.98 (s, 3H), 2.78 (s, 3H), 2.68 (t, *J* = 7.5 Hz, 2H), 1.57 – 1.52 (m, 2H), 1.35 – 1.27 (m, 6H), 0.87 (t, *J* = 6.7 Hz, 3H).

^{13}C NMR: (126 MHz, $CDCl_3$) δ 189.61, 170.94, 167.20, 163.34, 138.90, 128.95, 124.59, 115.99, 112.80, 107.42, 97.07, 56.70, 31.83, 29.30, 28.76, 25.86, 22.77, 22.56, 14.22.

HRMS (ESI): *m/z* calculated for $C_{19}H_{22}Cl_2NO_4$ (M+H) = 398.0920 Da; found 398.0920 Da.

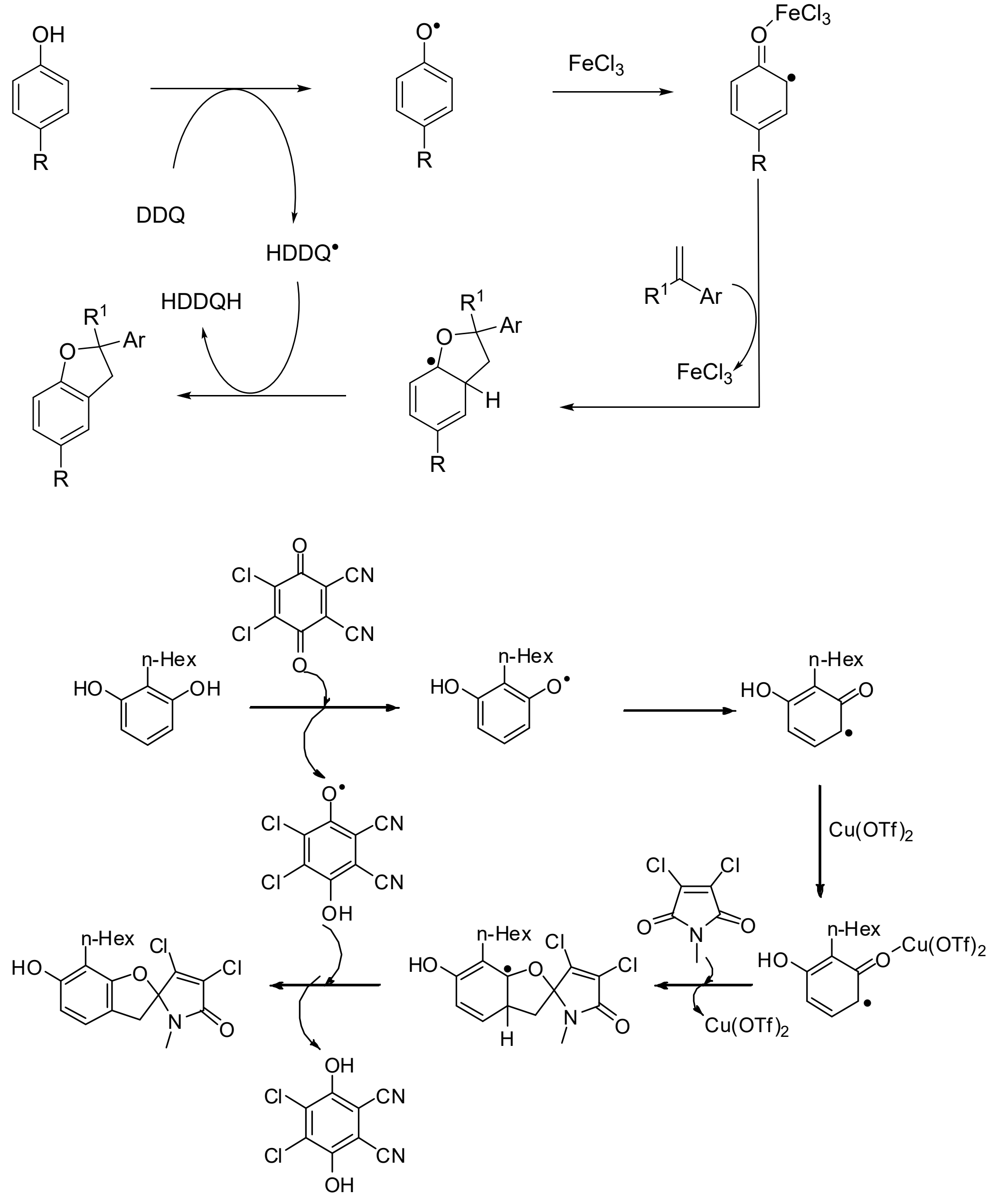

Supplementary Scheme S2. Mechanism of radical-induced addition of phenols to alkenes.
Top: General mechanism as shown in Ref [4]. Bottom: Adaptation to armeniaspirol synthesis.

NMR spectra

^{1}H-NMR of 2-hexylbenzene-1,3-diol (**12**)

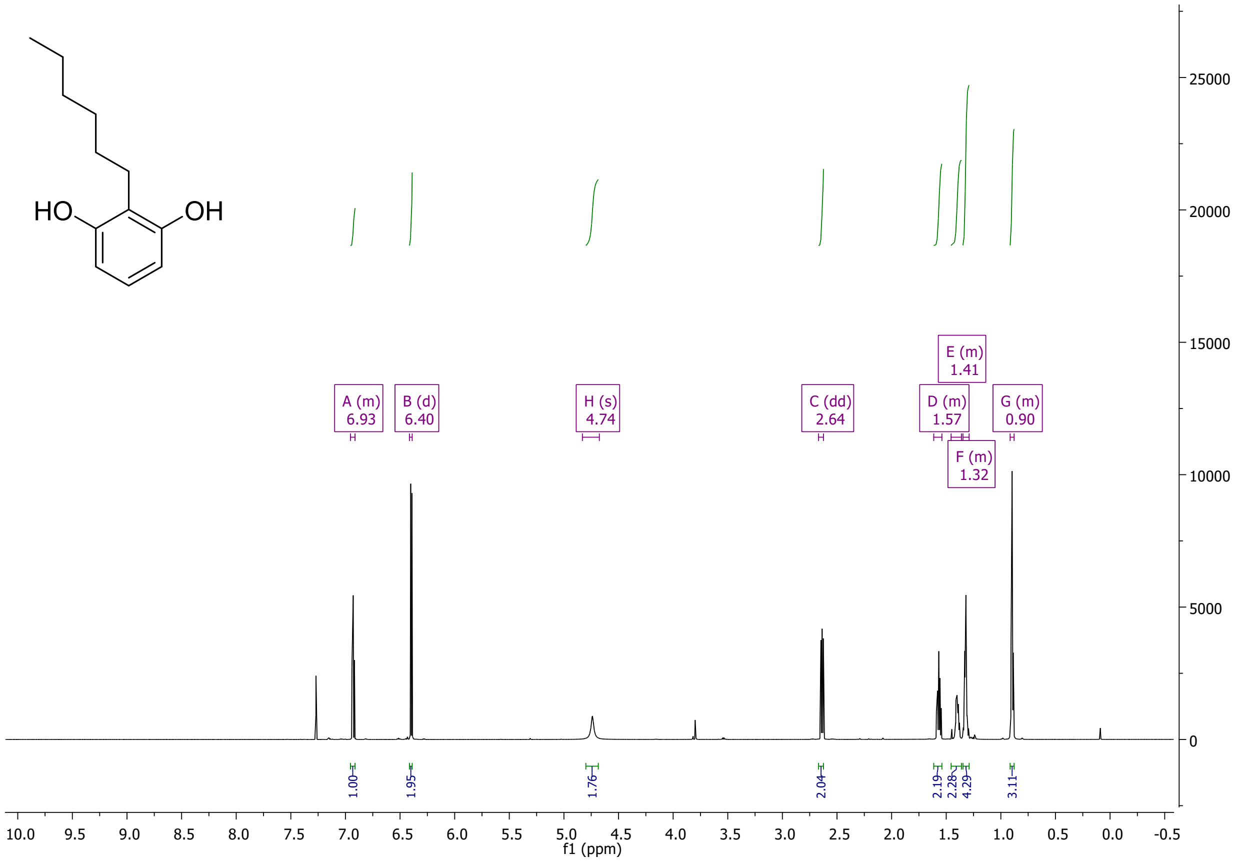

^{13}C-NMR of 2-hexylbenzene-1,3-diol (**12**)

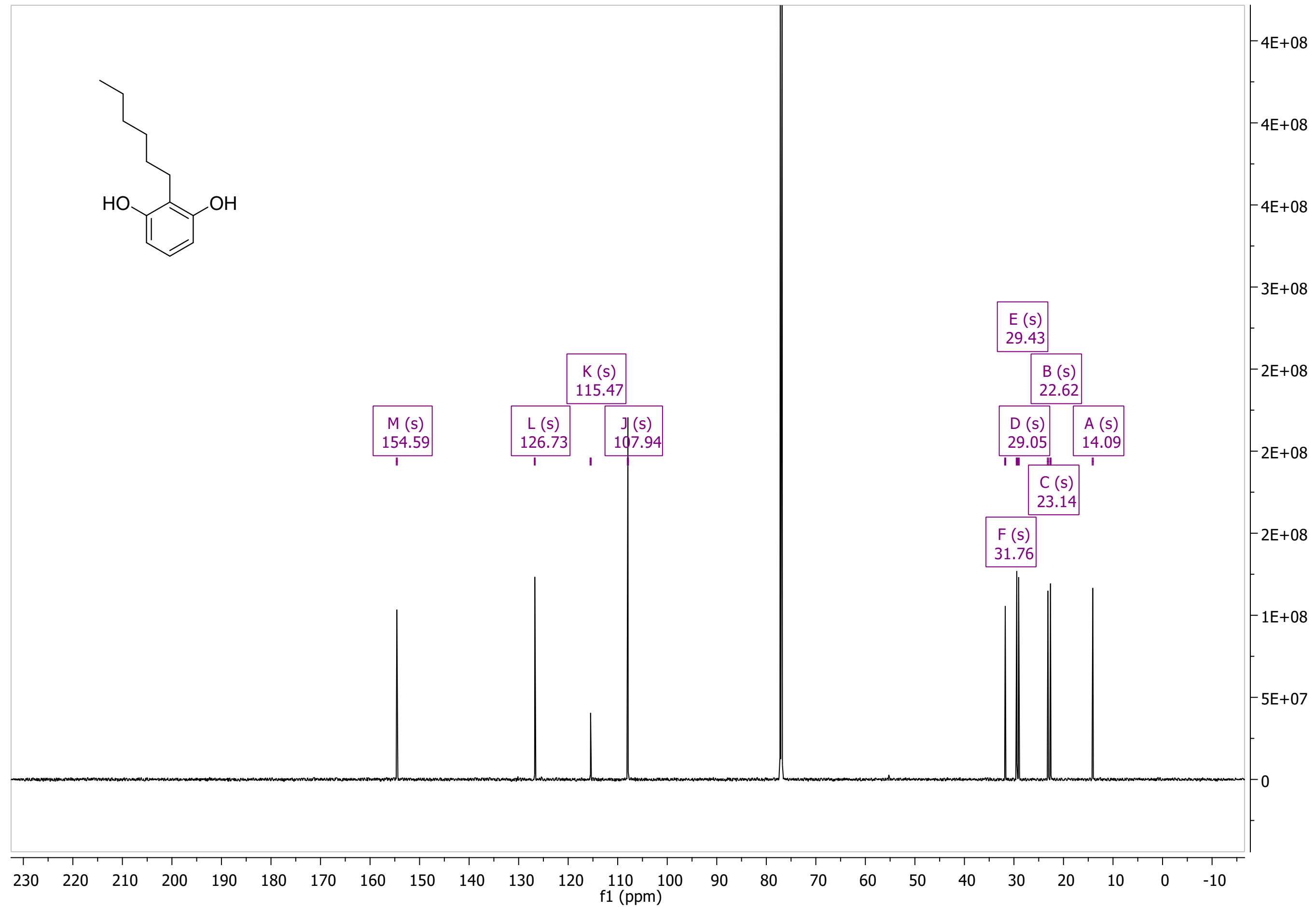

^{1}H-NMR of 3,4-dichloro-1-methyl-5-methylene-1,5-dihydro-2H-pyrrol-2-one (**9**)

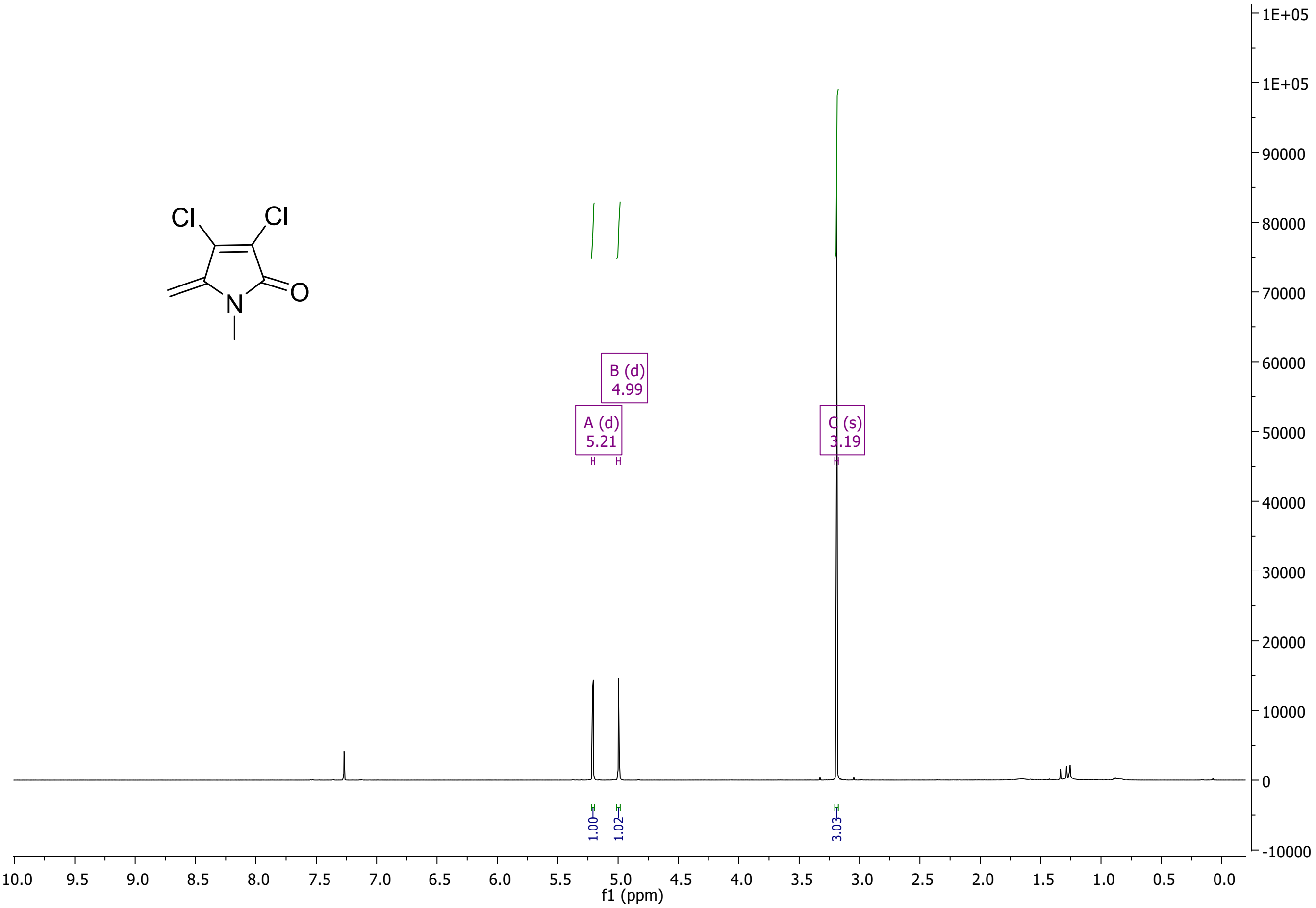

^{13}C-NMR of 3,4-dichloro-1-methyl-5-methylene-1,5-dihydro-2H-pyrrol-2-one (**9**)

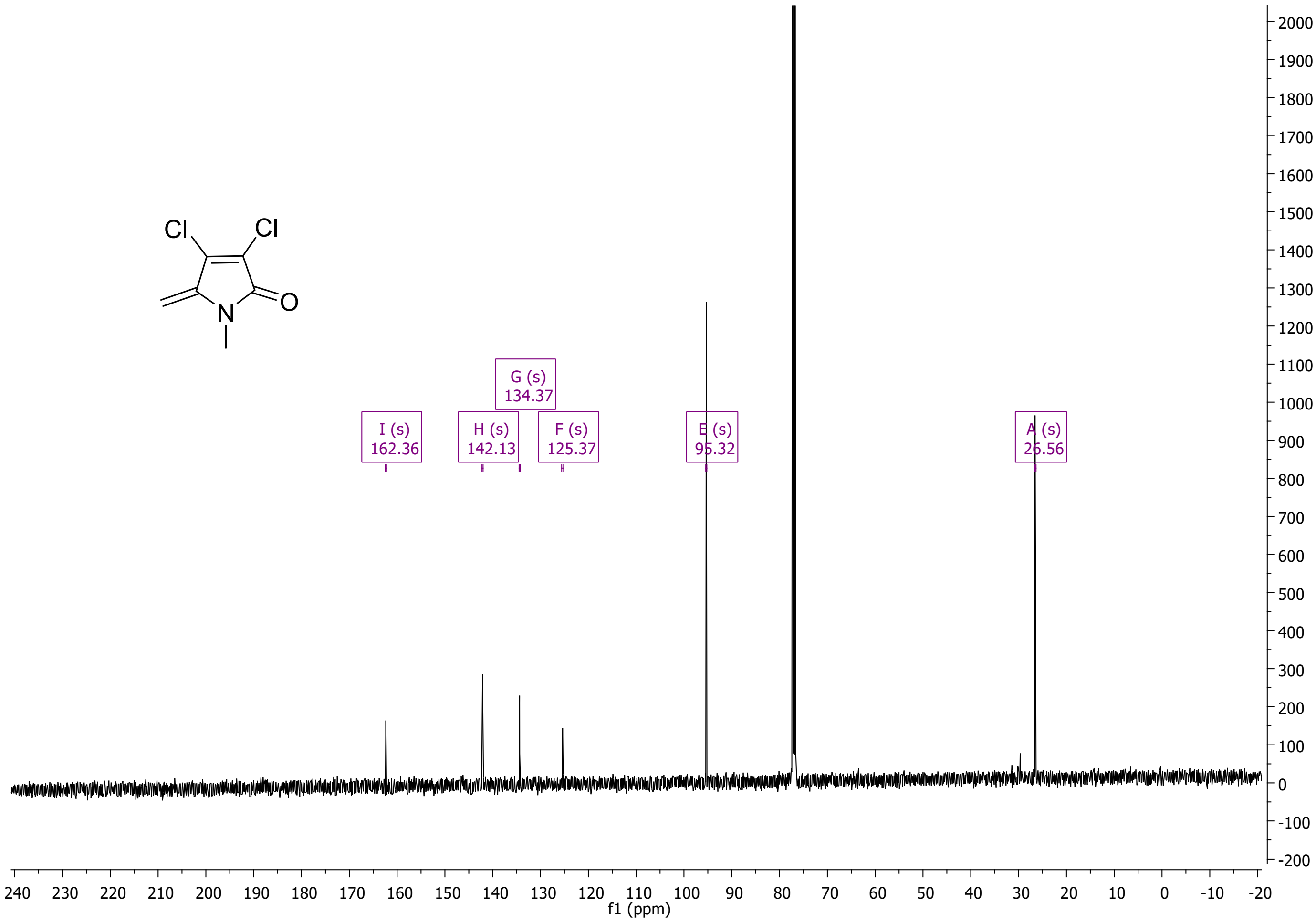

^{1}H-NMR of 7-deoxy-armeniaspirol A (**13**)

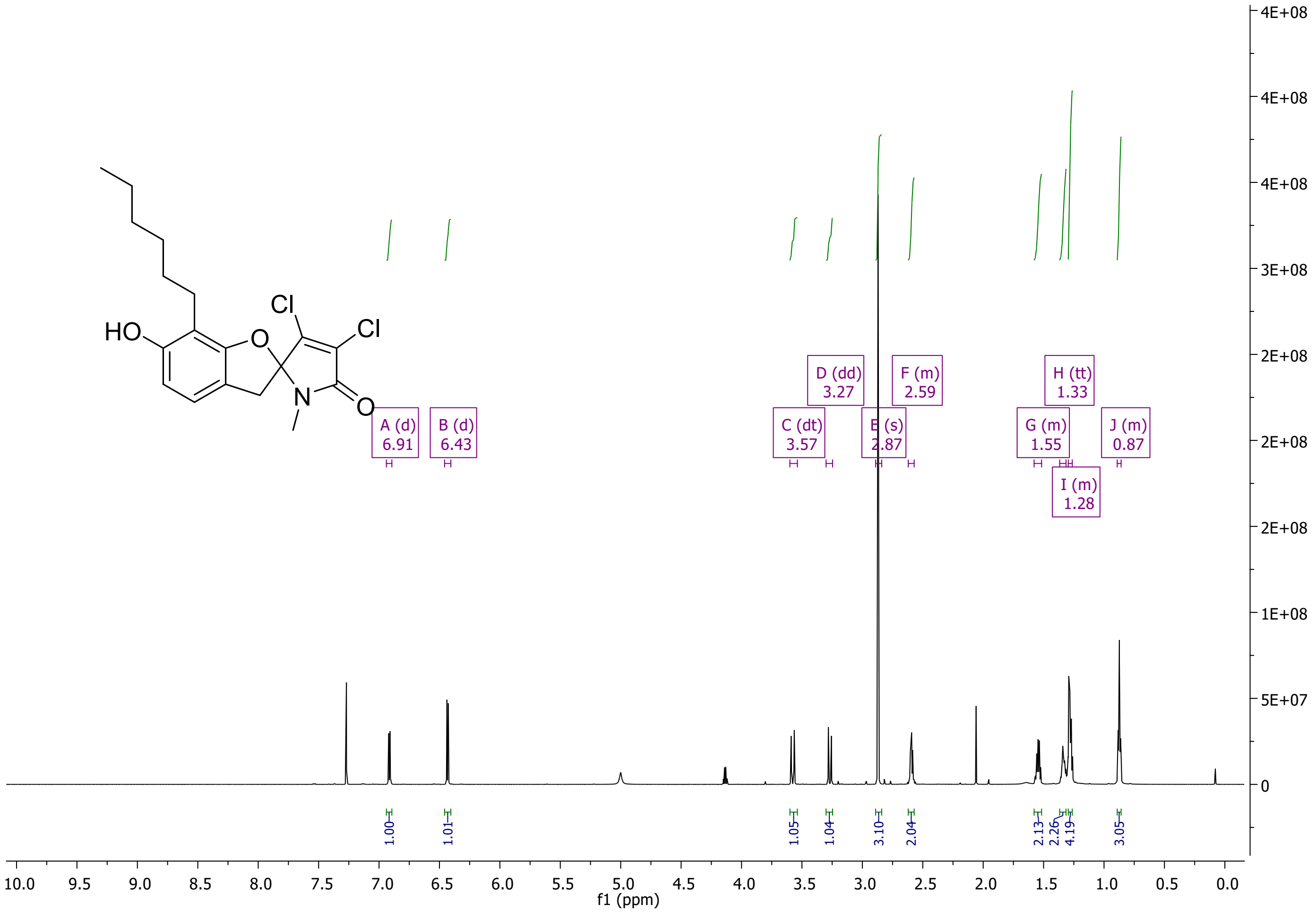

^{13}C-NMR of 7-deoxy-armeniaspirol A (**13**)

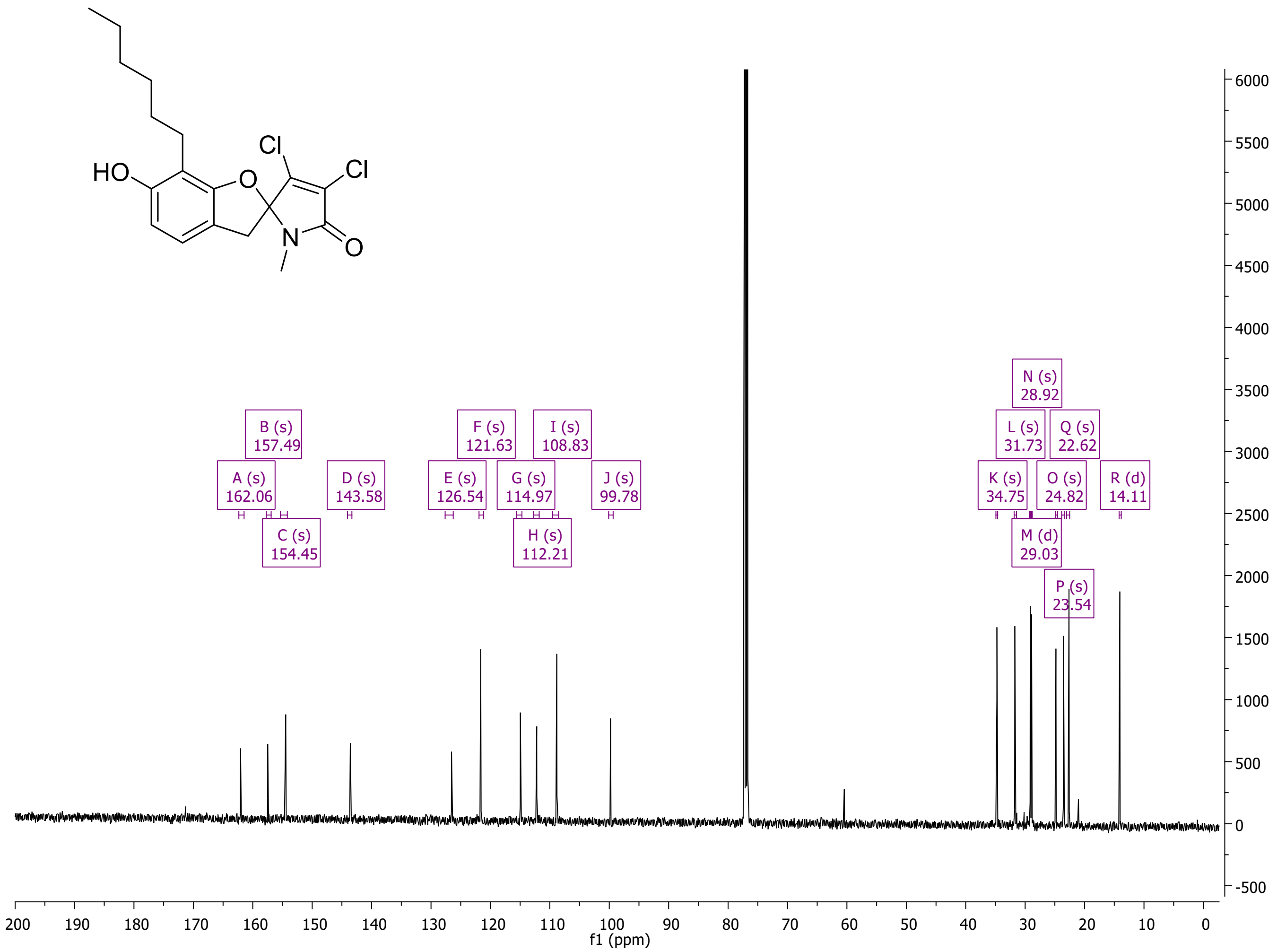

^{1}H-^{1}H COSY-NMR of 7-deoxy-armeniaspirol A (**13**)

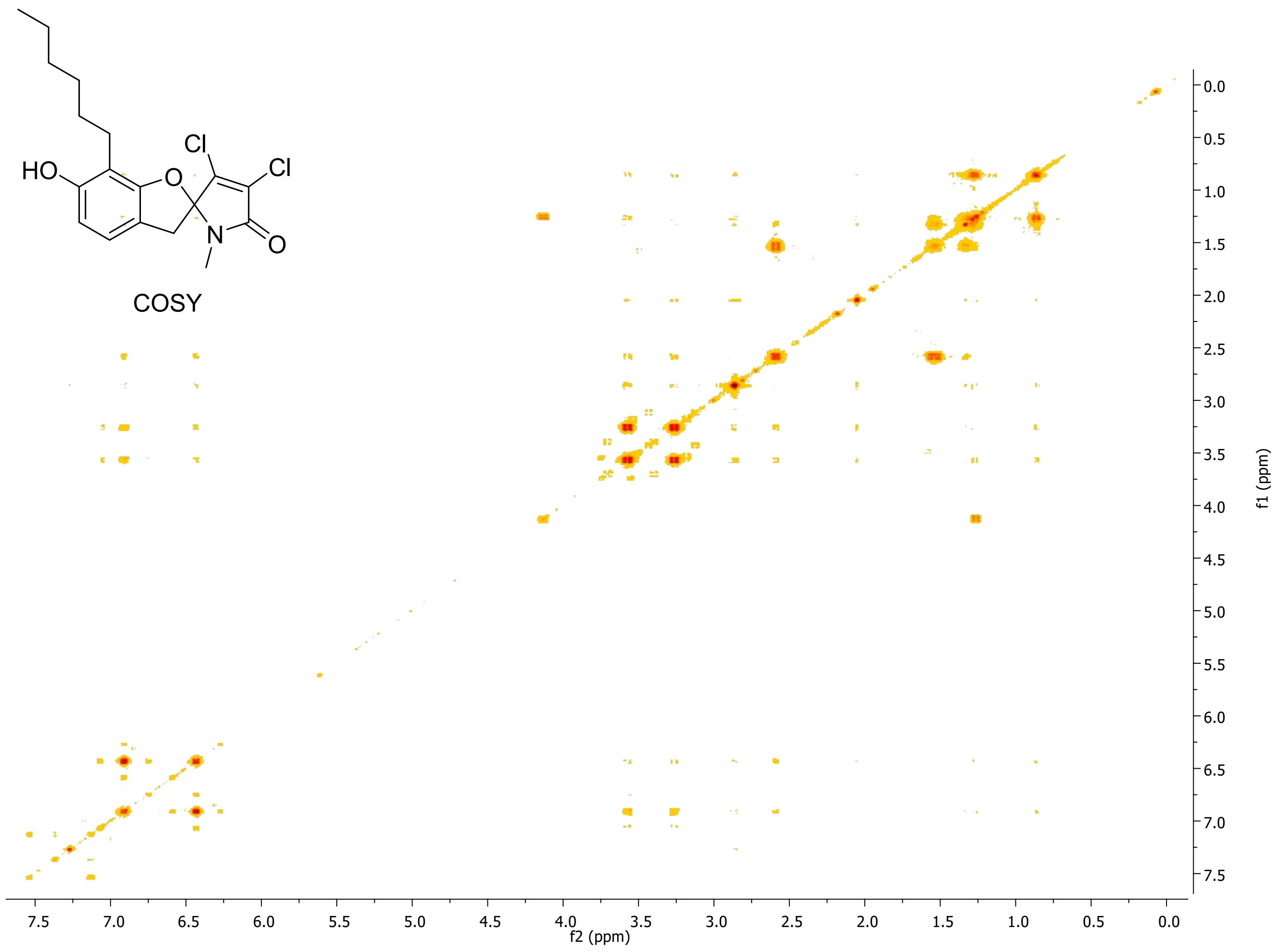

^{1}H-^{13}C HSQC-NMR of 7-deoxy-armeniaspirol A (**13**)

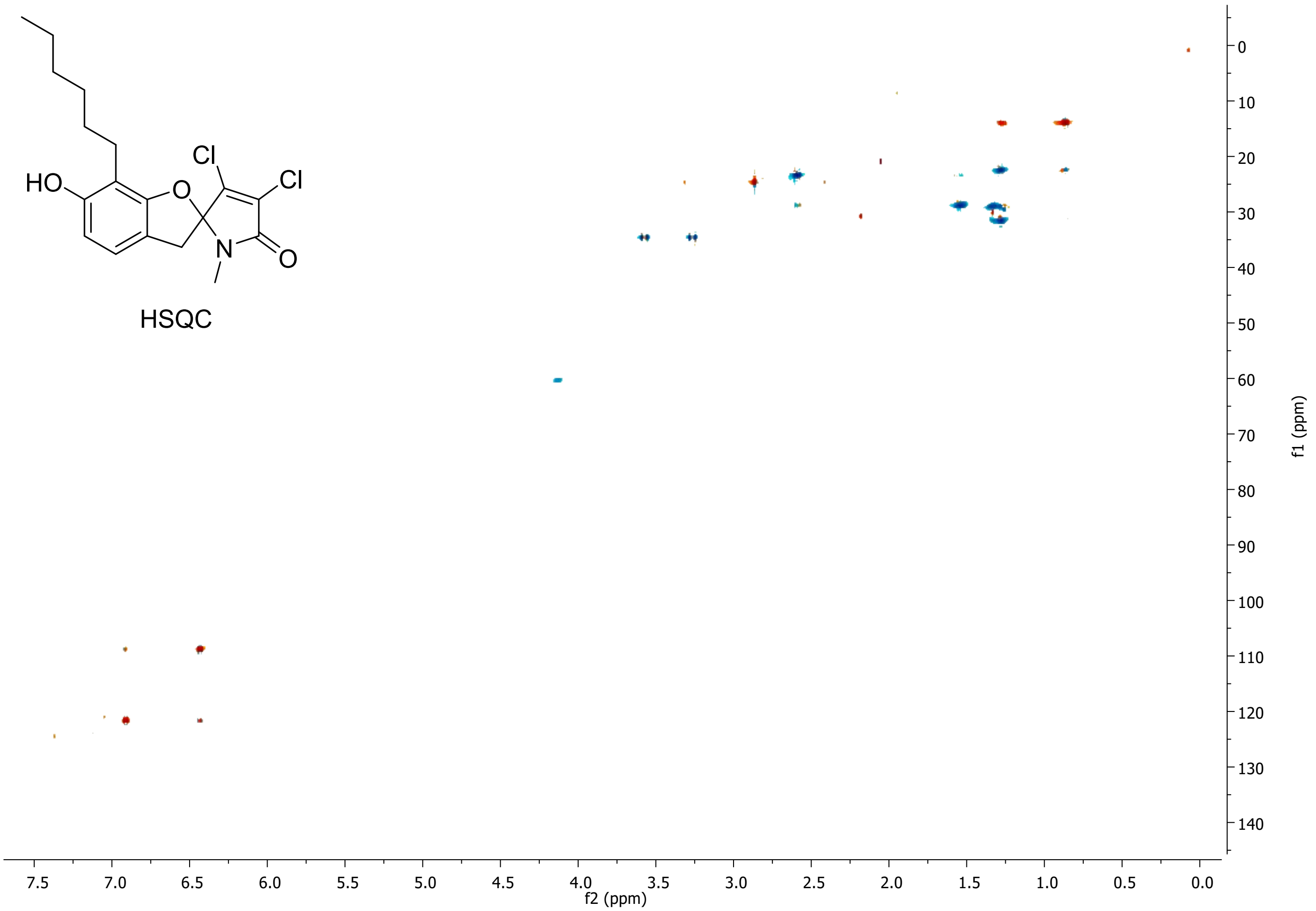

^{1}H-^{13}C HMBC-NMR of 7-deoxy-armeniaspirol A (**13**)

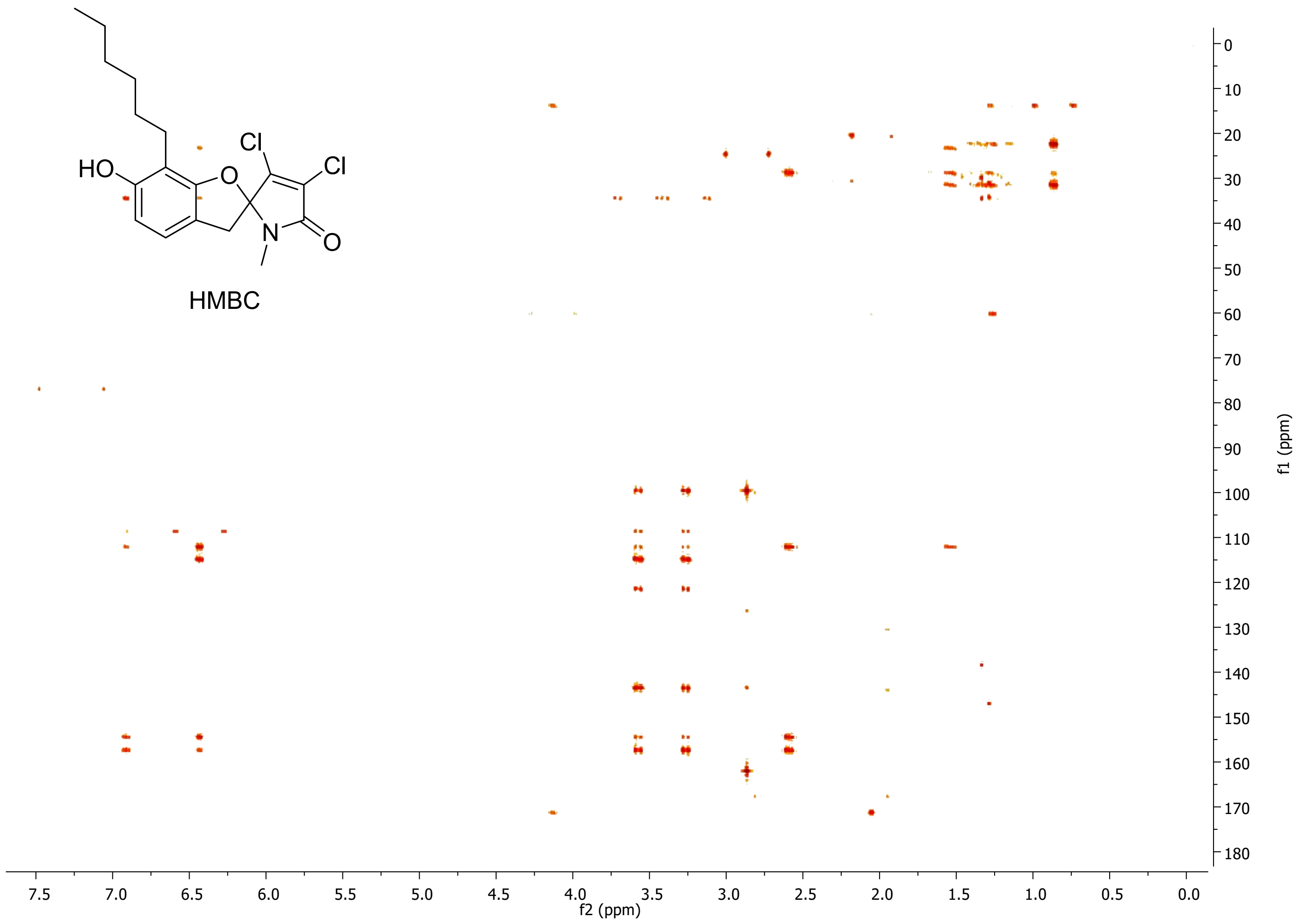

^{1}H-NMR of (±)-armeniaspirol A (**5**)

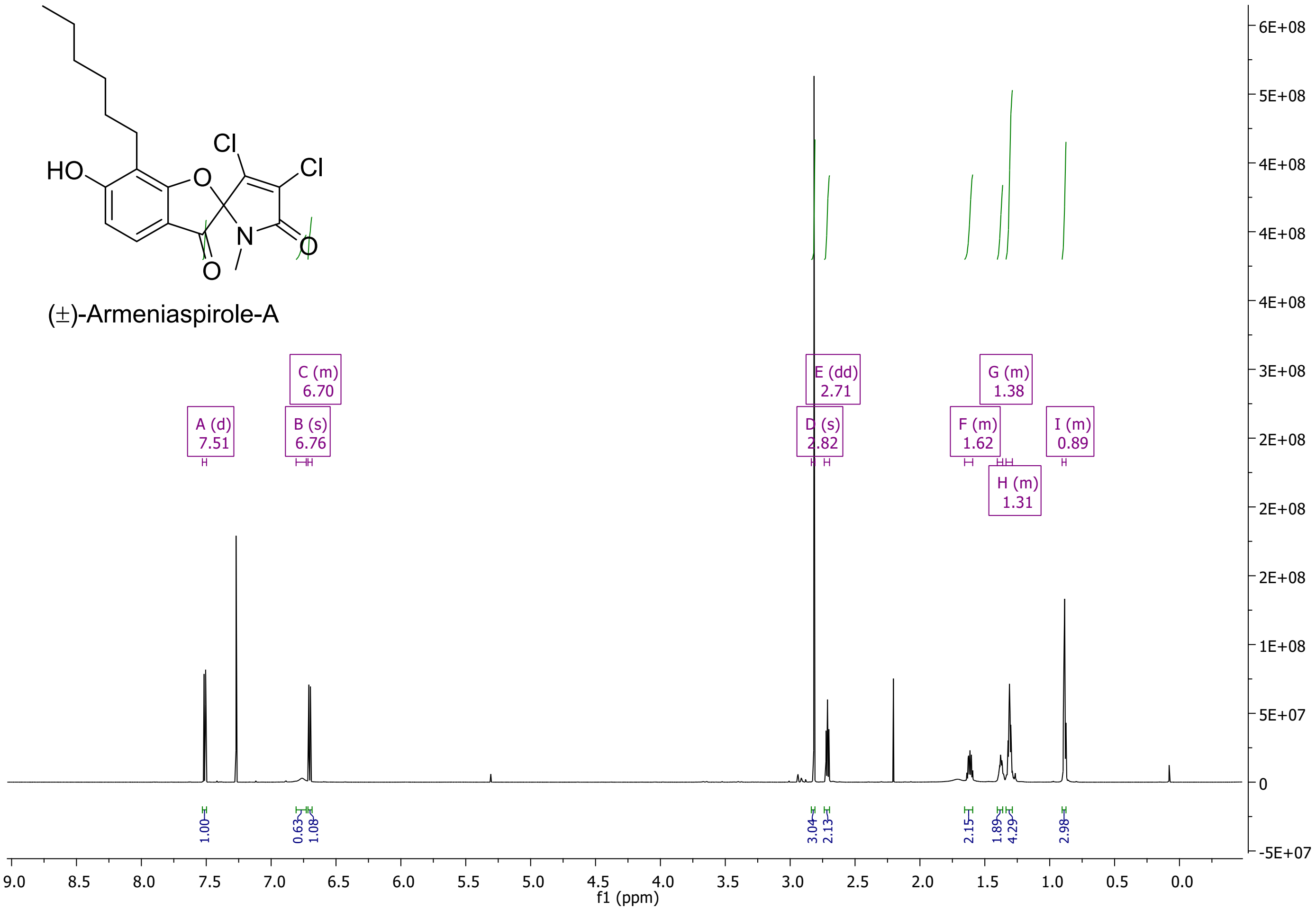

^{13}C-NMR of (±)-armeniaspirol A (**5**)

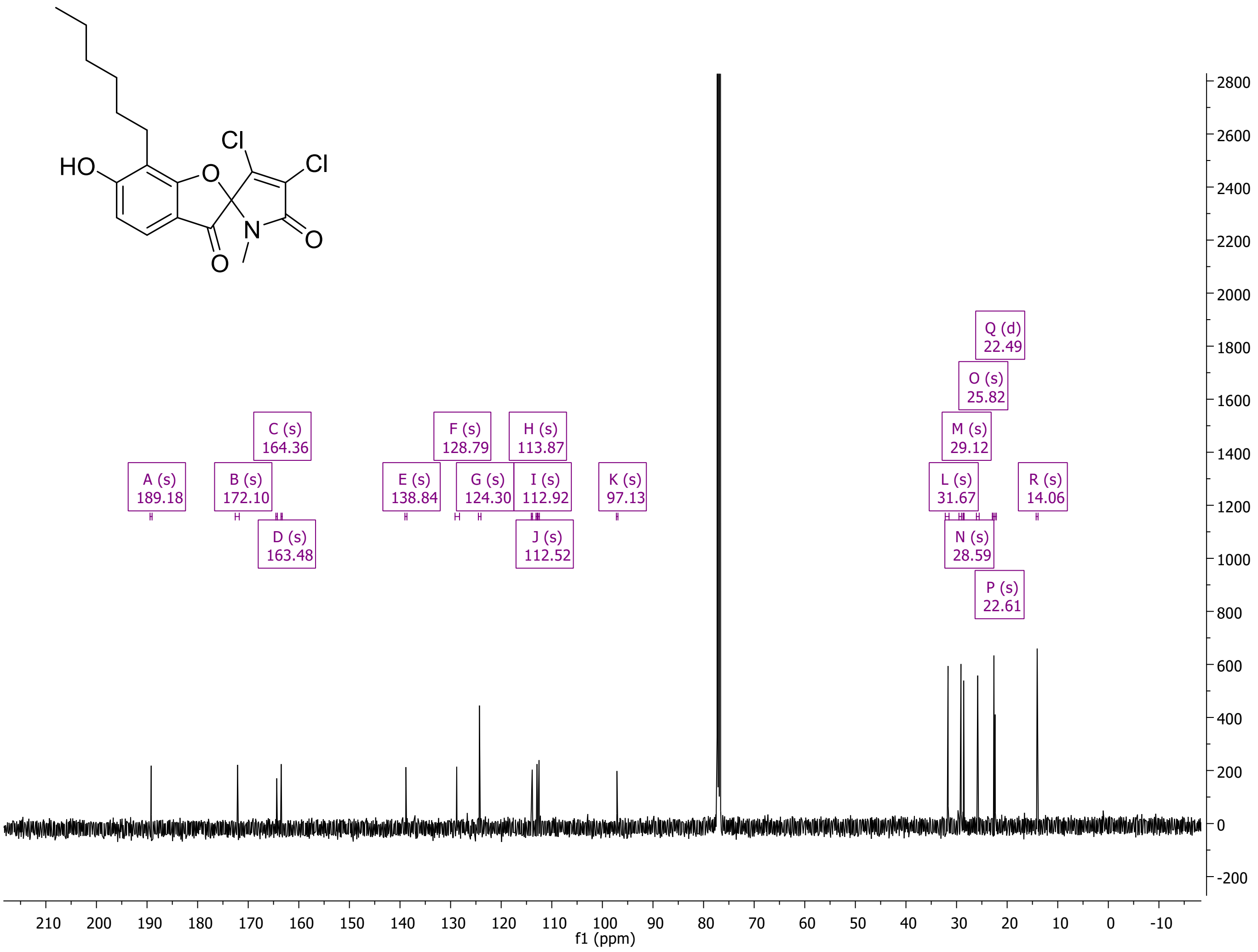

^{1}H-^{13}C HSQC-NMR of (±)-armeniaspirol A (**5**)

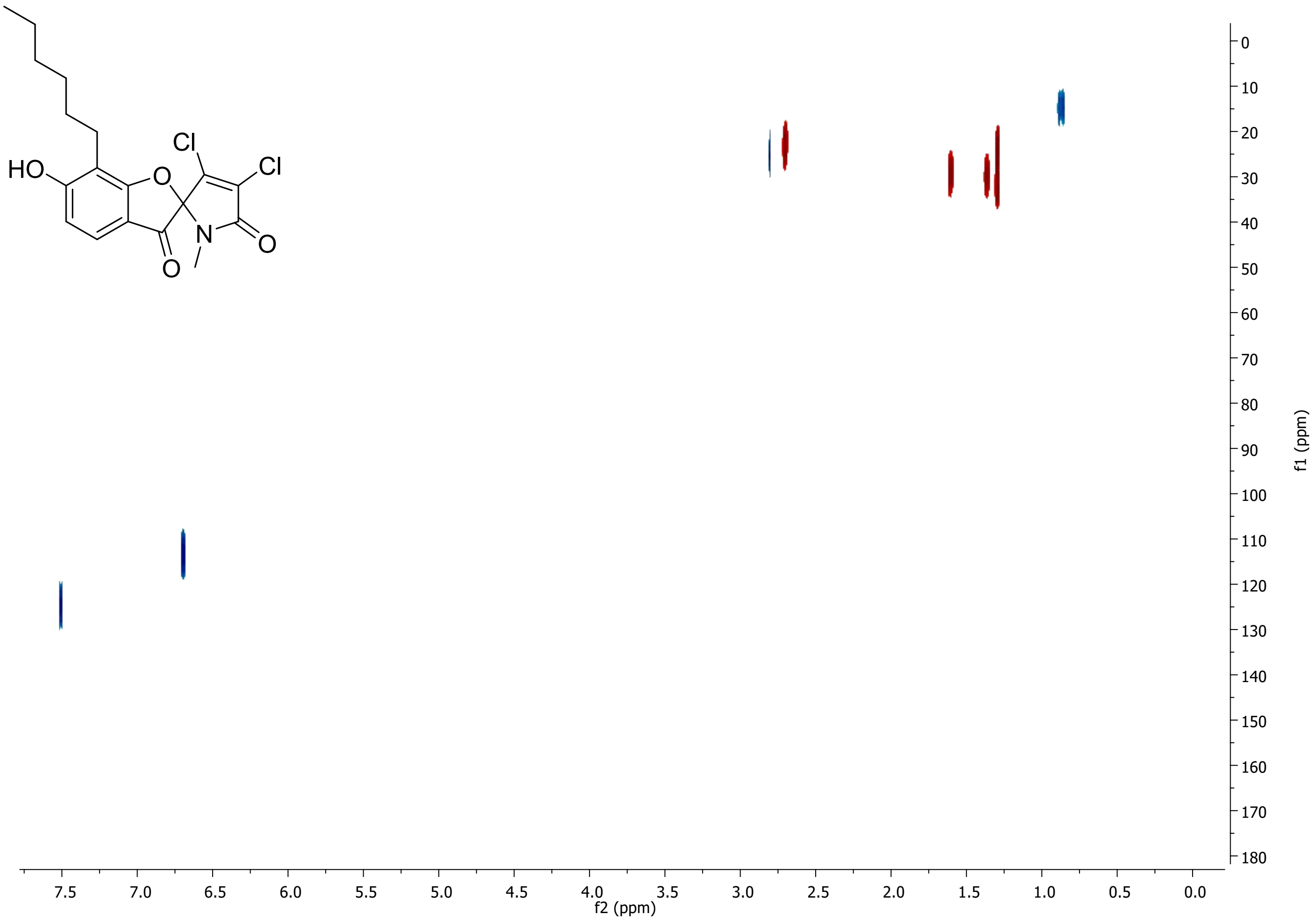

^{1}H-^{13}C HMBC-NMR of (±)-armeniaspirol A (**5**)

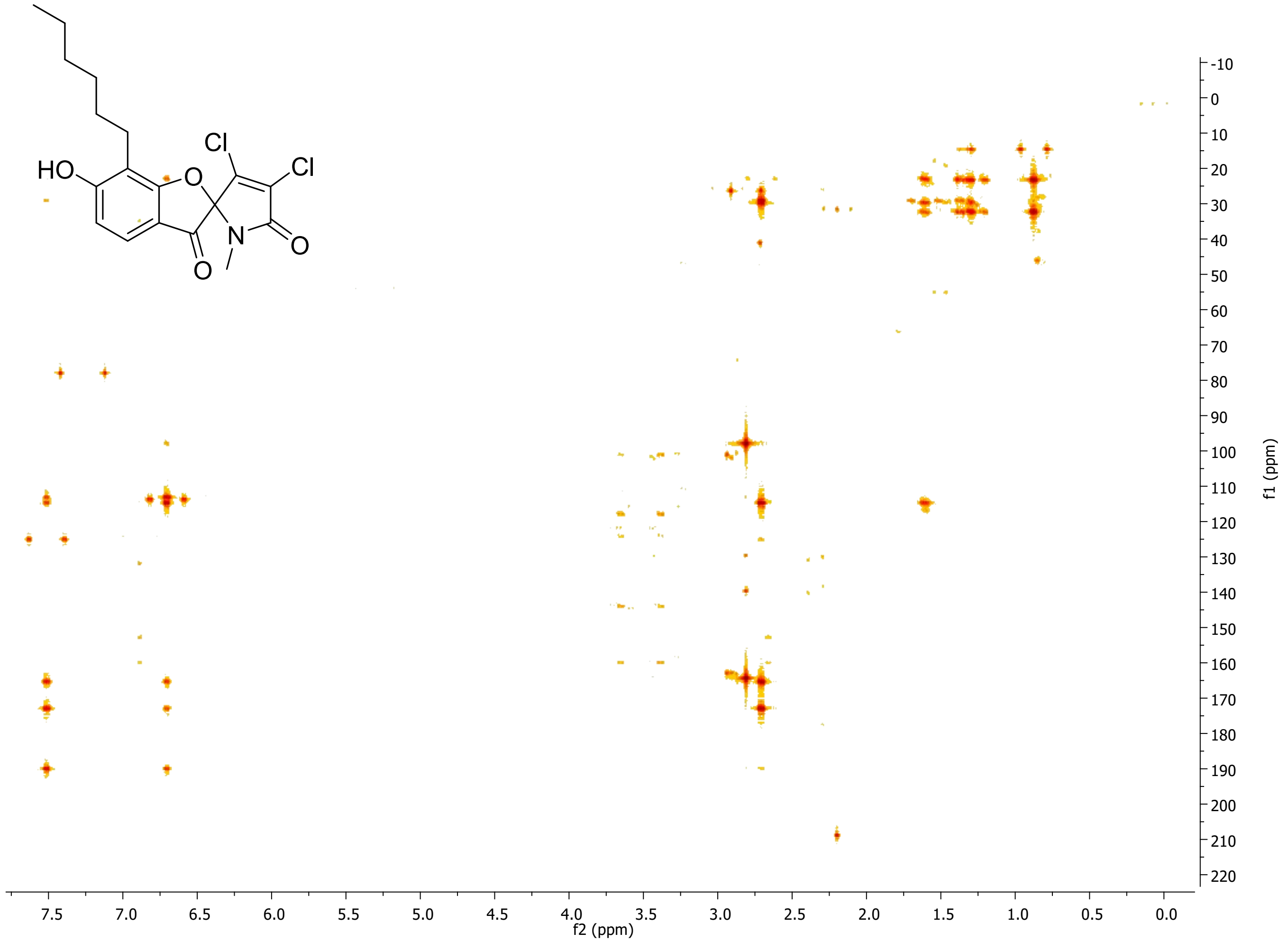

^{1}H-^{1}H COSY-NMR of (±)-armeniaspirol A (**5**)

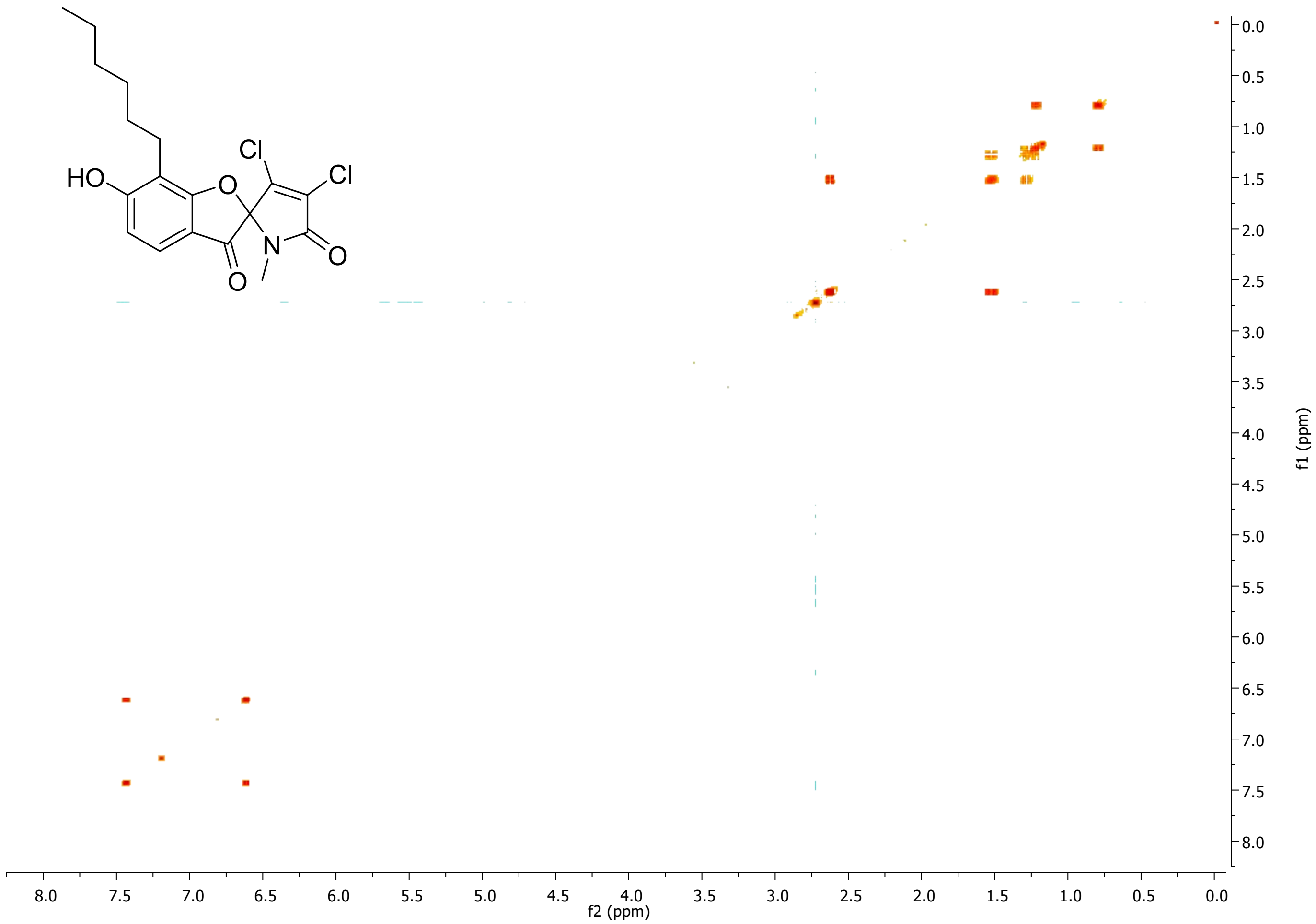

^{1}H-NMR of 1-methoxy-(±)-armeniaspirol A (**15**)

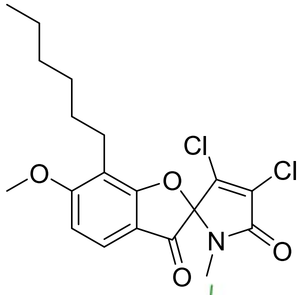

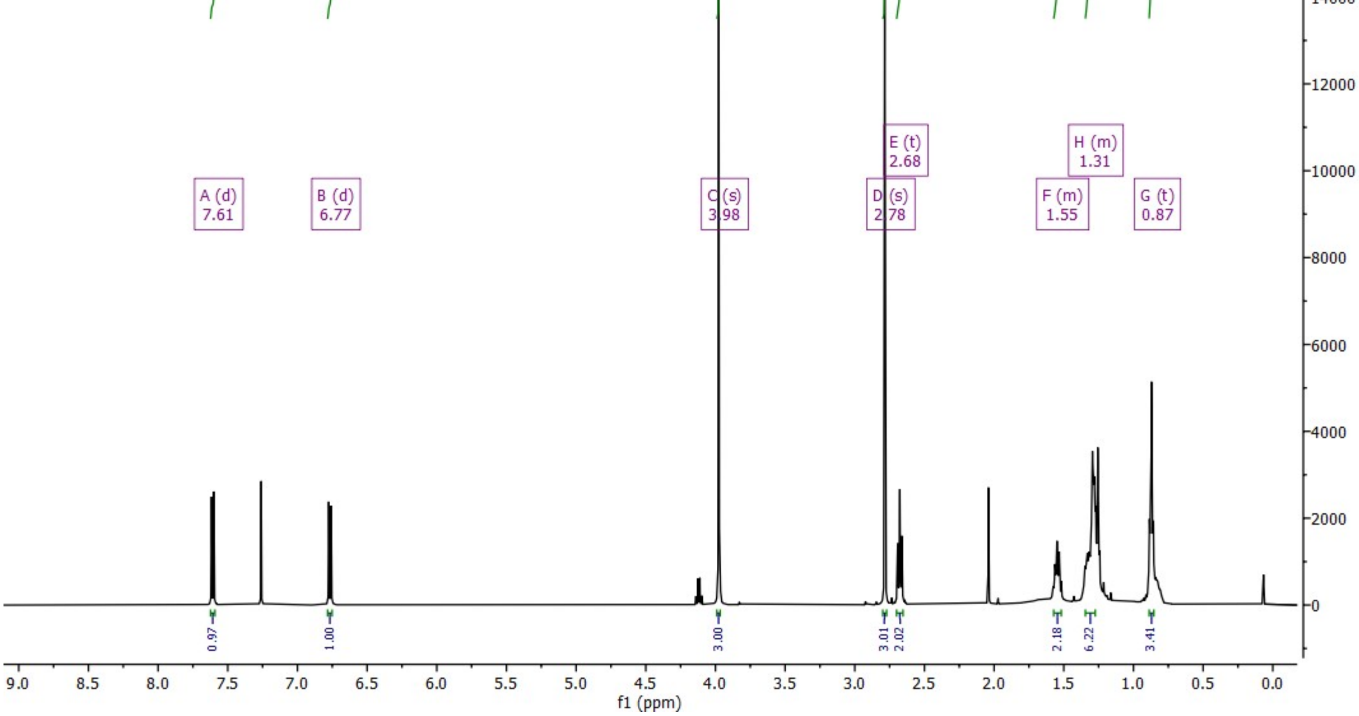

^{13}C-NMR of 1-methoxy-(±)-armeniaspirol A (**15**)

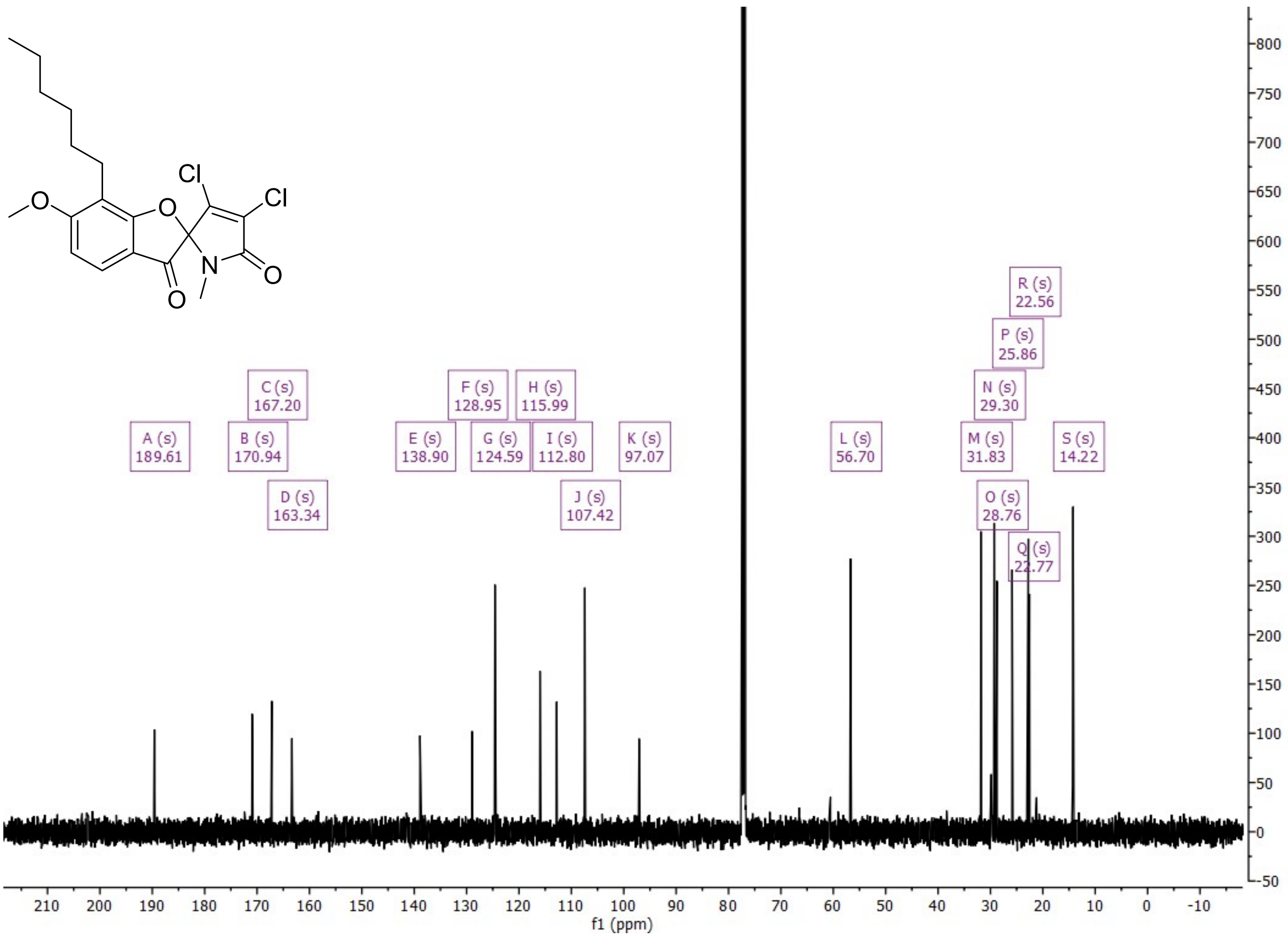

Mixing experiment of natural and synthetic armeniaspirol A

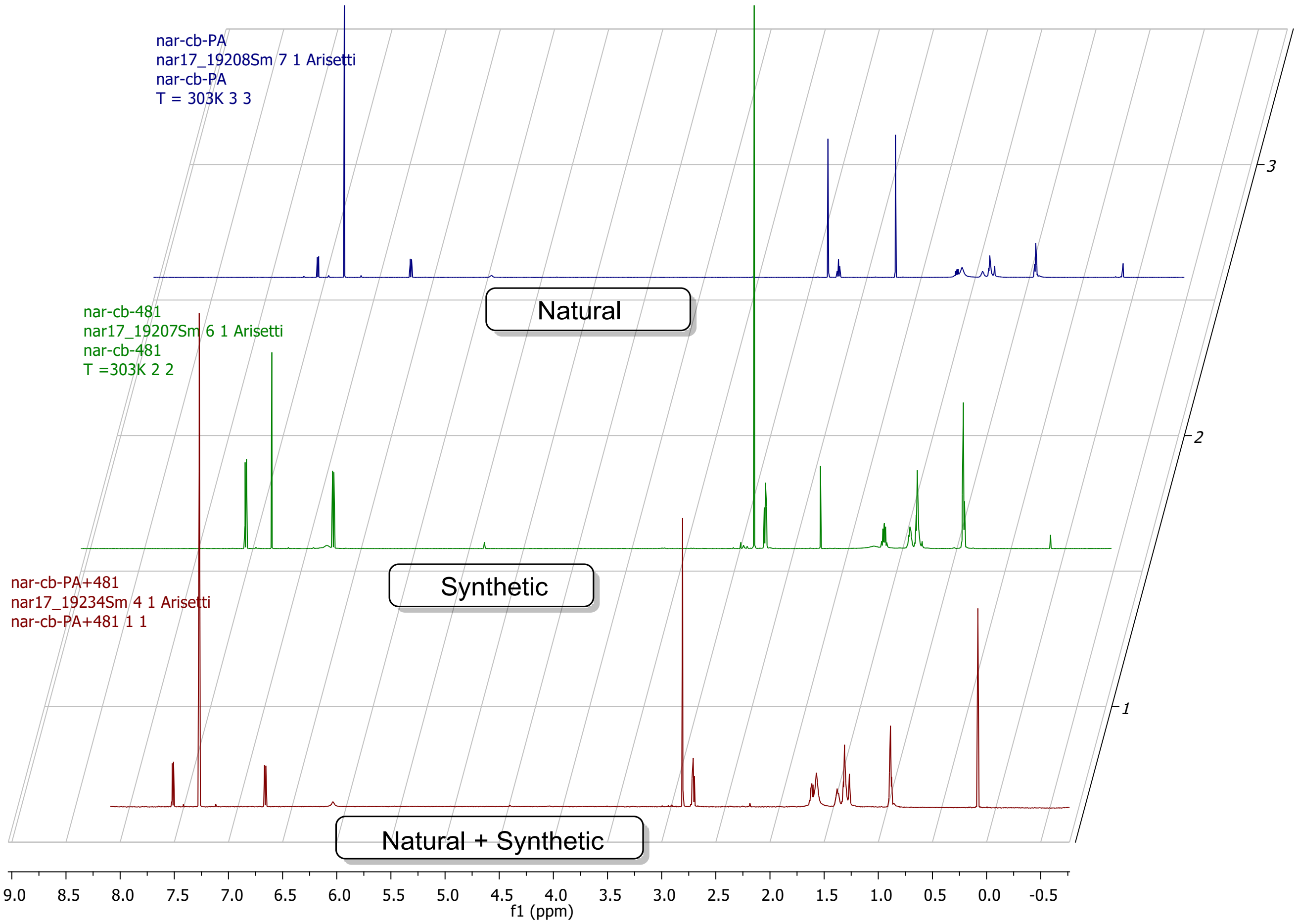

^{1}H-NMR of 3-hexyl-2,4-dimethoxybenzaldehyde (**7**)

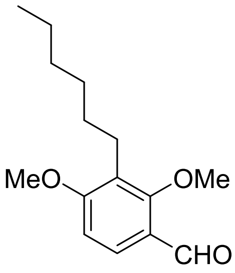

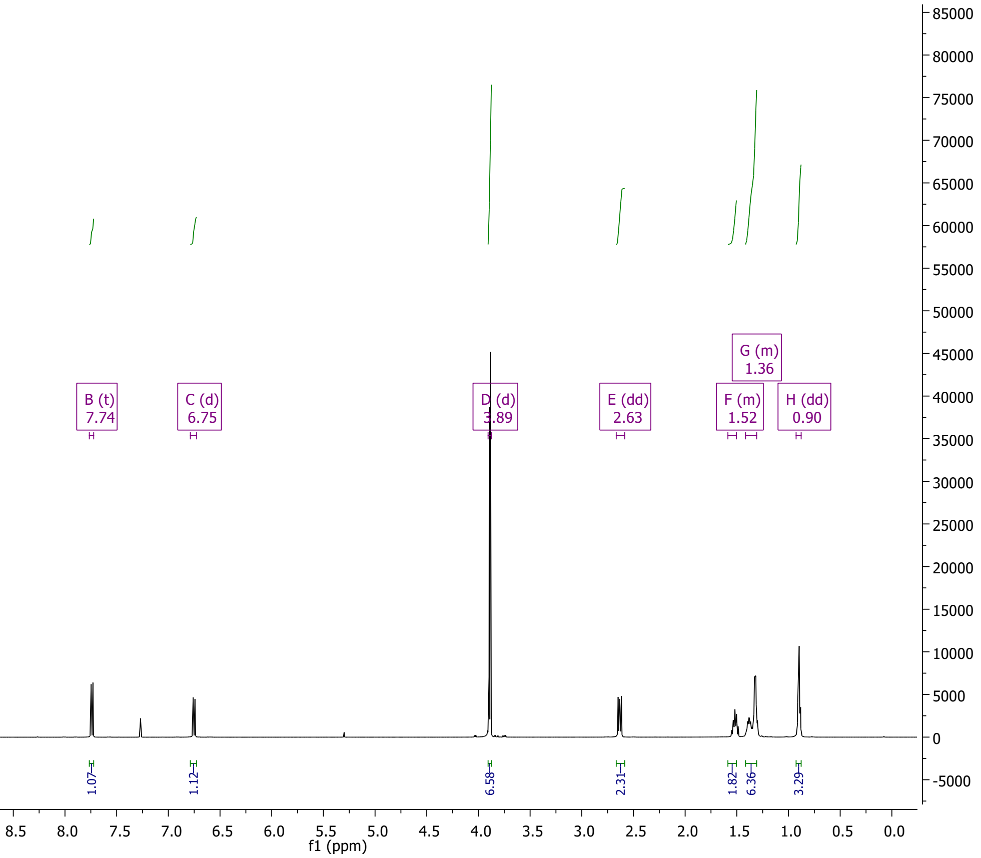

^{13}C-NMR of 3-hexyl-2,4-dimethoxybenzaldehyde (**7**)

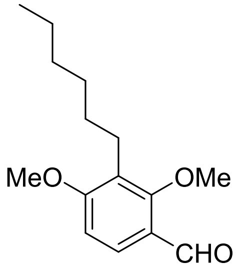

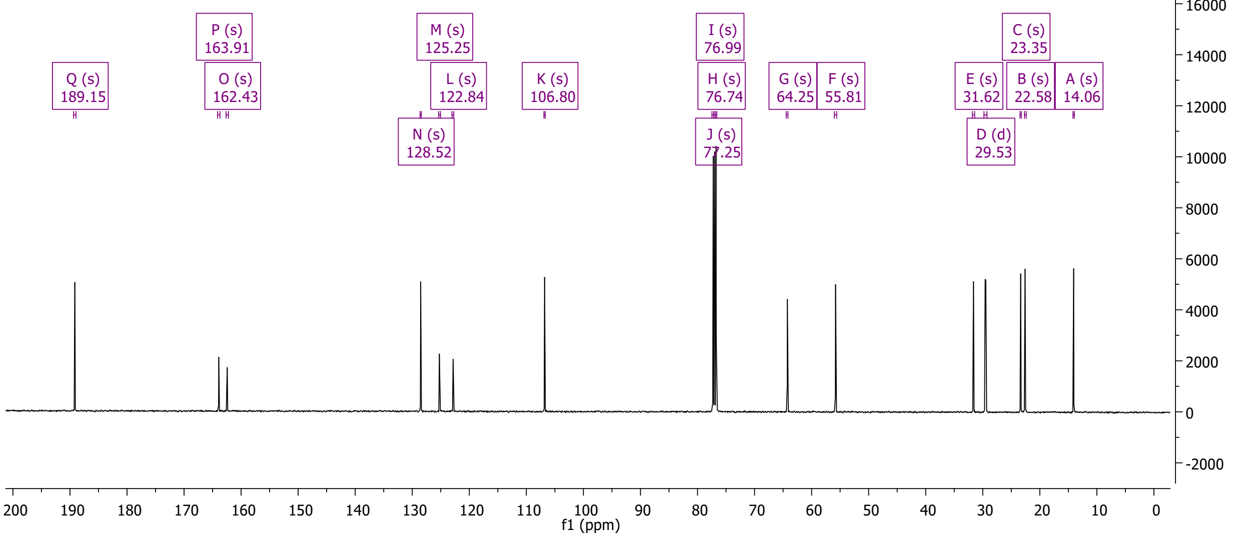

^{1}H-NMR of 2-(3-hexyl-2,4-dimethoxyphenyl)-1,3-dithiane (**S2**)

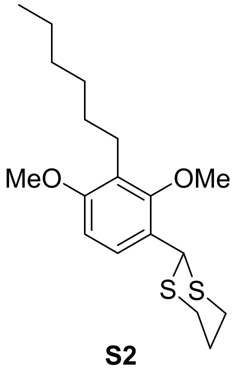

S2

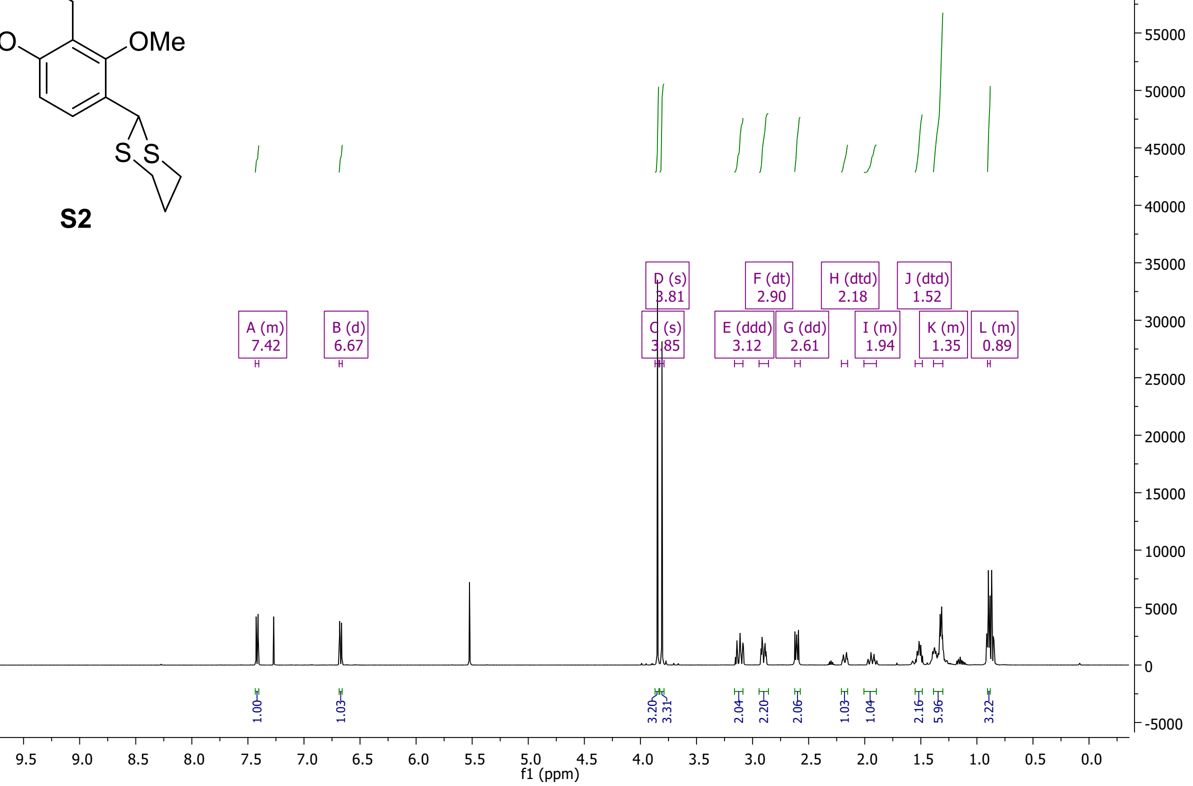

^{13}C-NMR of 2-(3-hexyl-2,4-dimethoxyphenyl)-1,3-dithiane (**S2**)

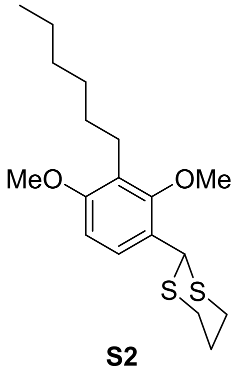

S2

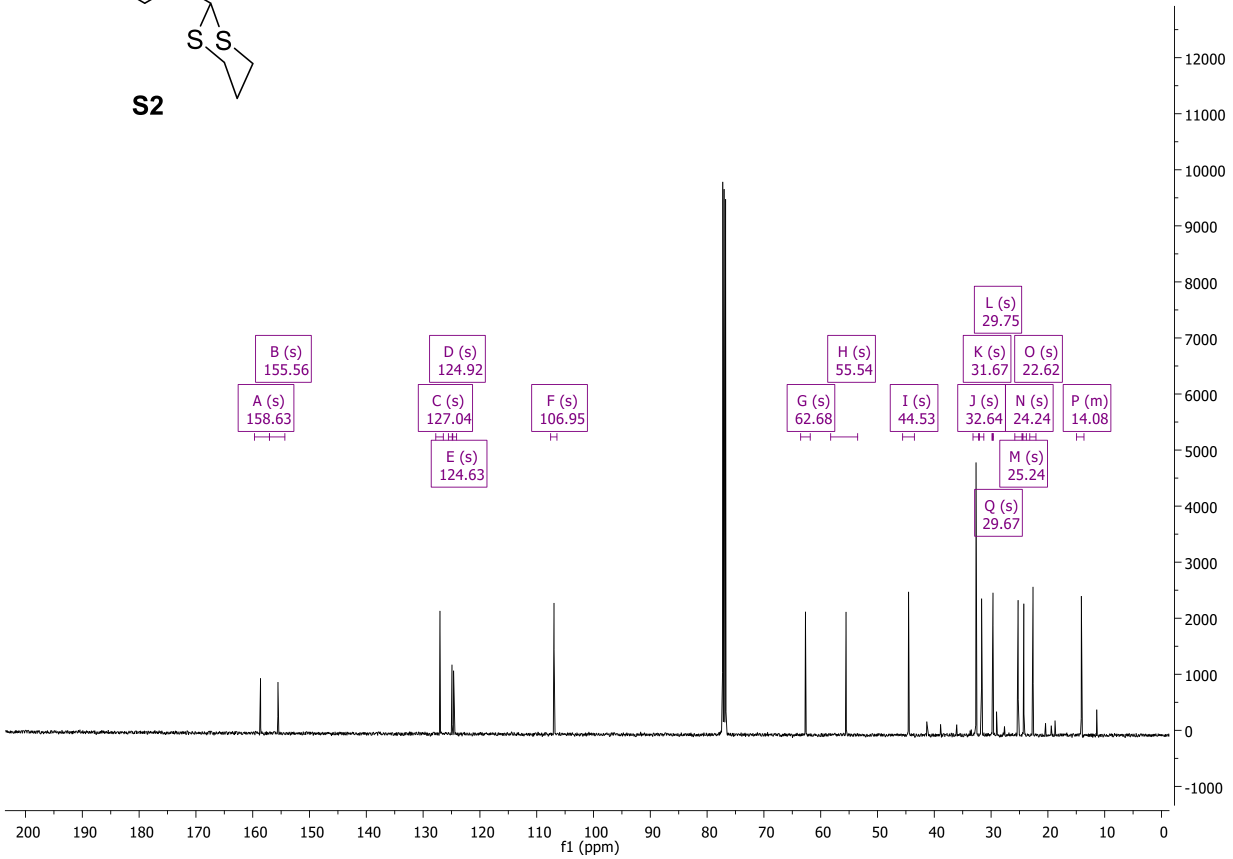

^{1}H-NMR of (E)-N'-(1-(3-hexyl-2,4-dimethoxyphenyl)ethylidene)-4-methylbenzenesulfonohydrazide(**S4**)

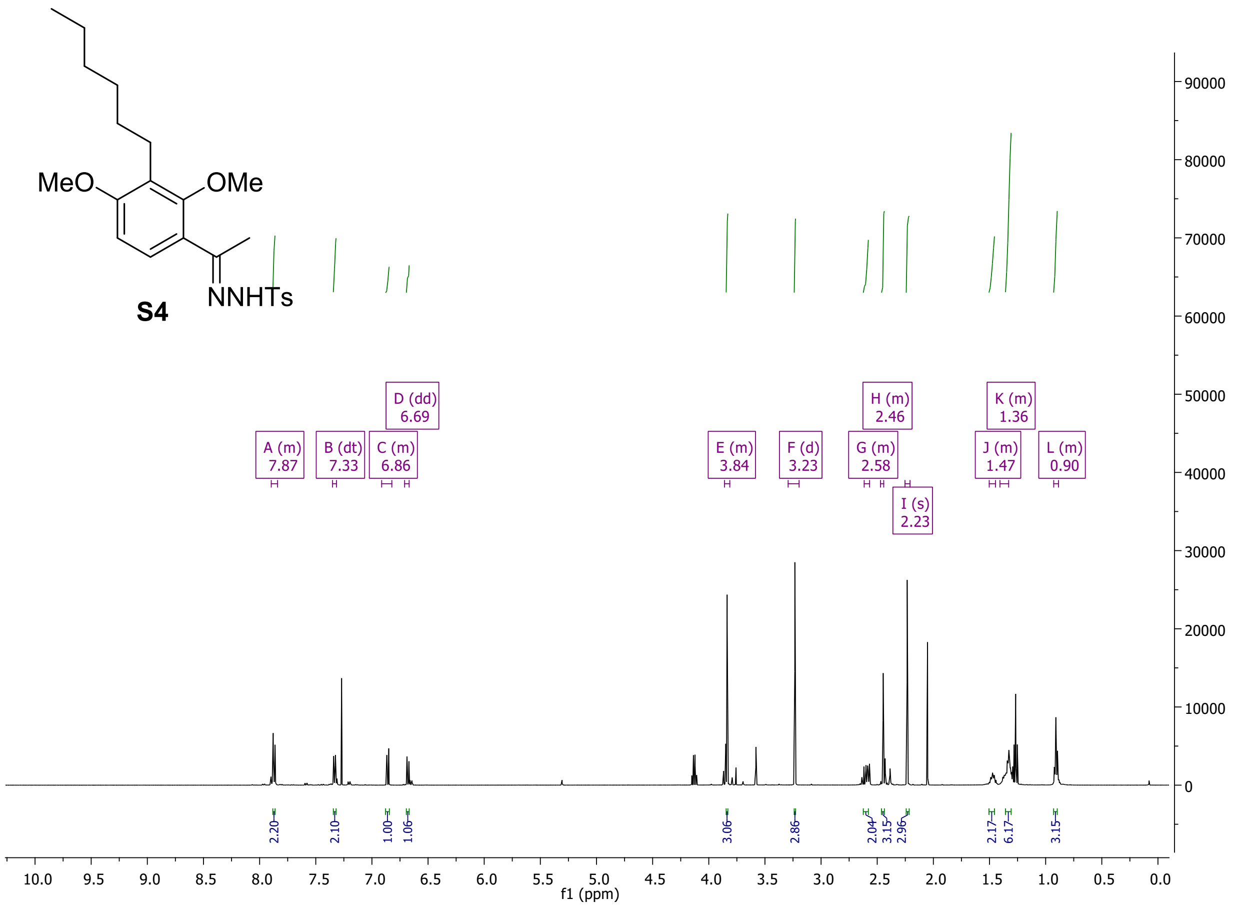

^{13}C-NMR of (E)-N'-(1-(3-hexyl-2,4-dimethoxyphenyl)ethylidene)-4-methylbenzenesulfonohydrazide (**S4**)

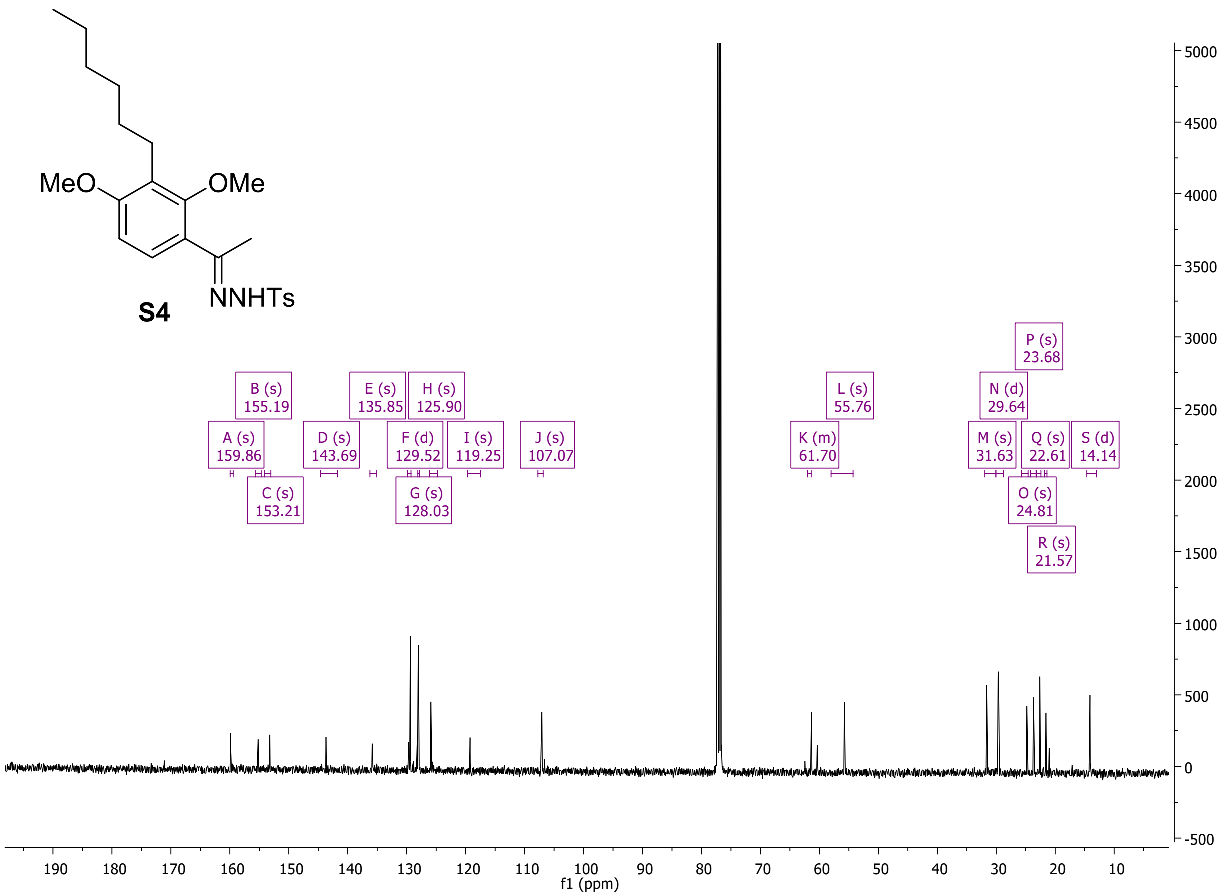

Experimental Procedures: Antibacterial efficacy, cytotoxicity, mode of action and mode of resistance

Antibacterial susceptibility testing in 384 well MTP format

An overnight culture of *Micrococcus luteus* DSM 1790 was inoculated at OD_{600} = 0.1 from a single-use frozen culture and grown aerobically at 30 °C in Müller Hinton broth pH 7.4 ± 0.2. The culture was adjusted to an OD_{600} of 0.02, which resulted in a final start OD_{600} of 0.01 in the test. 25 µL test culture was added to 25 µL of a serial dilution of the test compounds in 384 well assay plates (Corning, #3701) according to standardized procedures (DIN 58940-7 Medical microbiology – susceptibility testing of microbial pathogens to antimicrobial agents – determination of the minimum bactericidal concentration (MBC) with the method of micro boullion dilution) in 384 well plates. Armeniaspirol (**1**) was used at final concentrations of 20, 10, 5, 2.5, 1.25, 0.625, 0.313, 0.156, 0.078, 0.039 µg/ml. The test compounds pyoluteorin, streptopyrrole-1Cl, streptopyrrole-2Cl, marinopyrrole A and **16**-**20** were obtained from the Sanofi compound collection (Sanofi R&D, Frankfurt, Germany) and prepared as 6.4 mg/ml stocks in DMSO and used at final concentrations of 64, 32, 16, 8, 4, 2, 1, 0.5, 0.25, 0.125 µg/ml in the assay. For marinopyrrole A, a 0.64 mg/ml stock solution was prepared and the final concentrations in the assay were 6.4, 3.2, 1.6, 0.8, 0.4, 0.2, 0.1, 0.05, 0.025, 0.0125 µg/ml, accordingly. The serial dilution was performed using a pipetting robot (Eppendorf epMotion 5070, Germany). Ciprofloxacin (1 mg/ml stock) was applied as a positive control. The highest DMSO concentration in the assay was 1%, which had no apparent effect on the growth of the bacteria. After an incubation time of 20 h at 30 °C with 70% humidity and 700 rpm, the optical density at 600 nm was measured with a Plate Reader (Tecan SPARK). The MIC, defined as the lowest concentration that completely suppressed bacterial growth, was calculated by fitting the growth curve to a modified Gompertz function as described by Lambert & Pearson[5] using GraphPad Prism 9.02, and then rounding the determined value to the nearest concentration applied in the assay. All MIC values were determined in technical duplicates with at least two independent biological replicates.

For *S. aureus* DMS346, the same assay procedure was used as described above, albeit with slight modifications: The bacteria were cultivated in TSB medium and grown at 37°C. Armeniaspirols **1** and **5** and the derivatives **13** and **15** were used at final concentrations of 20, 10, 5, 2.5, 1.25, 0.625, 0.313, 0.156, 0.078, 0.039 µg/ml in two biological replicates. For **13** and **15** a third replicate had concentrations of 2, 1, 0.5, 0.25, 0.125, 0.0625, 0.0313, 0.0156, 0.0078, and 0.0039 µg/ml.

Supplementary Table S1: Growth inhibitory activities of armeniaspirols on *M. luteus* DSM1790 and *S. aureus* DSM346.

Compound	***M. luteus* DSM1790 MIC [µg/ml]**	***S. aureus* DSM346 MIC [µg/ml]**
**13**	2.5	5
**15**	>20*	>20
Armeniaspirol A natural **1**	1.25	1.25
Armeniaspirol A synthetic **5**	0.625	1.25
Ciprofloxacin	0.625	0.3
CCCP	1.25	1.25
Linezolide	-	2.5

*The growth of *M. luteus* DMS1790 was reduced by **15**, with an IC_{50} of ca. 1.5 µg/ml but not inhibited, leading to an MIC of >20 µg/ml.

Antibacterial susceptibility testing of *E. coli* strains in 96 well MTP format

All *E. coli* strains that were used during this study were either obtained from our in-house collection, the Keio collection, the German Collection of Microorganisms and Cell Cultures (DSMZ), or they were kindly provided by Dr. Ruben Hartkoorn from the Center for Infection and Immunity of Lille at the Institut Pasteur de Lille. Strains were handled according to standard procedures. The addition of Kanamycin was required for some strains and this was obtained from Sigma-Aldrich (Merck KGaA, Darmstadt, Germany). Bacteria were inoculated from cryocultures and plated for 24 hours on fresh CASO agar and incubated at 37°C. The following day, 1-2 colonies were picked from the overnight plate and suspended into 0.9% NaCl (Merck KGaA, Darmstadt, Germany) to achieve a McFarland value of 0.2 - 0.5. The McFarland suspension was re-suspended into fresh medium corresponding to approximately 5 x 10^6 colony-forming units (CFU)/mL. A total of 75 µL test culture was added to 75 µL of a serial dilution of the test compounds in 96 well assay plates (Corning, #3788). All compound and antibiotics were used from a 5 mg/ml stock solution and were tested at final concentrations of 64, 32, 16, 8, 4, 2, 1, 0.5, 0.25, 0.125 µg/ml in the assay. Kanamycin was applied as a positive control. The highest DMSO concentration in the assay was 1%, which had no apparent

effect on the growth of the bacteria. After an incubation time of 24 hours at 37 °C, the MIC, defined as the lowest concentration that completely suppressed bacterial growth, was determined by visual inspection. All MIC values were determined in technical duplicates with three independent biological replicates.

Effect of pH on antibacterial activity of *Micrococcus luteus* in 384 well MTP format

To determine the effect of the pH of the medium on the activity of **1**, cultures of *Micrococcus luteus* DSM1790 were grown in 384 well MTP format as described above. The pH of the MH medium (7.4, Roth) was adjusted to 6.5, 7, 8, 9 and 10 using HCl and NaOH solutions, and ciprofloxacin was included as a positive control. The MICs were determined as described above for two biological replicates with two technical replicates. DMSO controls were included for each pH, DMSO up to 1% did not impede bacterial growth. The final OD_{600} of the cultures was strongly dependent on the pH, so that the bacterial growth was normalized to the final OD_{600} of respective DMSO control.

A

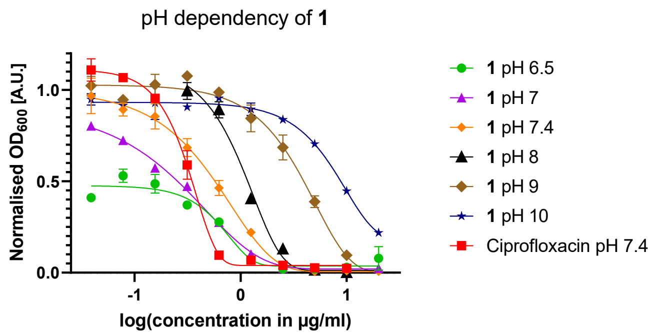

B

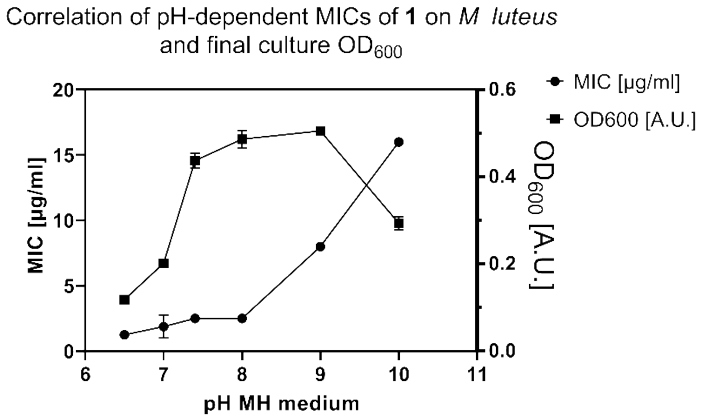

C

Medium pH	Av. MIC [µg/ml]	Av. IC_{50} [µg/ml]	Av. OD_{600} [A.U.]
6.5	1.3	0.5	0.118
7	1.9	0.6	0.202
7.4	2.5	1.1	0.437
8	2.5	4.0	0.487
9	8	14.2	0.505
10	16	0.3	0.293

Supplementary Figure S1: pH dependency of antibacterial activity of 1 on *M. luteus* DSM1790. **A)** MICs were determined in MH medium at different pHs using the microbroth dilution method. The final OD_{600} values were normalised to the final OD_{600} of the untreated vehicle control and the curves were fitted to a modified Gompertz function using Graphpad prism to determined MIC values. **B)** The average final OD_{600} of DMSO treated controls is correlated with the calculated MICs. This shows that the bacteria grow to different final ODs at the different pHs with the growth optimum between pH 8 and 9. **C**) Table of the determined average MICs and average final OD_{600} of *M. luteus* grown at medium pH 6.5 -10.

Membrane depolarization assays

*Bacterial membrane potential assay (Bac*light™ *assay) in 384 well format*

A *Bac*light™ assay was performed using the *Bac*light™ Bacterial Membrane Potential Kit from Invitrogen™ (catalogue number B34950), with modifications to make it suitable for 384 well MTP format. The assay is based on accumulation of 3,3′-diethyloxacarbocyanine iodide ($DiOC_2(3)$) dye in all bacterial cells, which then exhibit a green fluorescence. Cells which maintain their membrane potential accumulate the dye internally, which then leads to aggregation of the dye, shifting its fluorescence from green to red. By calculating the ratio of the red to green fluorescence of the cells, the portion of living cells compared to mock treated cells (DMSO) can be quantified. To this end, an overnight culture of *Micrococcus luteus* DSM 1790 was inoculated at OD_{600} = 0.1 from a single-use frozen culture and grown aerobically at 30 °C in Müller Hinton broth pH 7.4 ± 0.2. The next day, a new 50 ml culture was inoculated from the overnight culture at OD_{600} = 0.1 and grown aerobically at 30°C shaking with 150 rpm until OD_{600} = 0.5, then pelleted by centrifugation at 4500 *g*. The pellets were resuspended in either PBS (unstained control) or PBS containing 20 µM $DiOC_2(3)$ dye and then incubated for 10 min at room temperature in the dark. In parallel, serial 1:3 dilutions of the compounds (**1**, **5,** pyoluteorin, streptopyrrole-1Cl, streptopyrrole-2Cl, marinopyrrole A and **16**-**20**) were prepared using a pipetting robot (Eppendorf epMotion 5070, Germany) to final concentrations of 10, 3.3, 1.1, 0.370, 0.123, 0.041, 0.014, 0.005, 0.002, 0.0005, 0.0002 µg/ml in MH medium. CCCP was used as a positive control and both ciprofloxacin and DMSO as negative controls. 25 µl of the dilutions were pipetted into a black-walled, clear-bottom 384 well plate, and 25 µl of stained cells were added to the compounds, mixed for 15 s at 600 rpm and then incubated at room temperature for 15 min in the dark. For the unstained controls, 25 µl of cells were mixed with 25 µl medium. The red and green fluorescence of the $DiOC_2(3)$ dye was measured at λ_{ex} = 485 nm, λ_{em} = 520 nm and λ_{ex} = 485 nm, λ_{em} = 600 nm, respectively, using a fluorescence reader (TECAN Spark). The membrane potential was determined by subtracting the unstained cell controls and then calculating the ratio of red-to-green fluorescence as % of the vehicle control (DMSO). The IC_{50} values were determined using GraphPad Prism 9.02 by fitting the inhibitor to a normalized response using a variable slope.

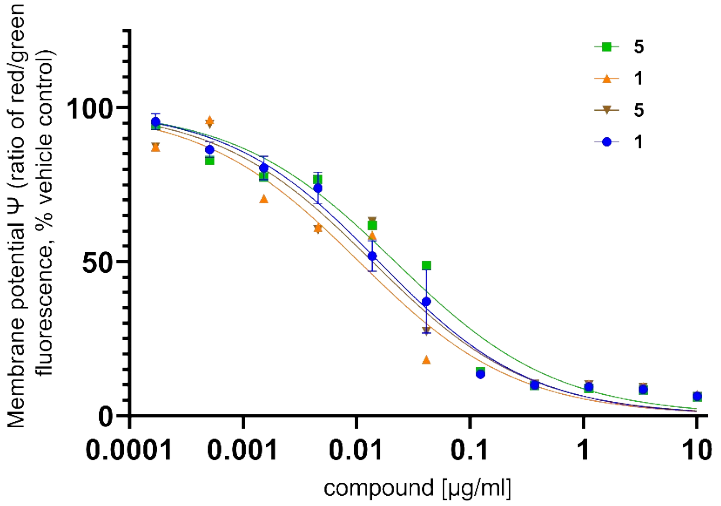

Supplementary Figure S2: Comparison of membrane depolarising effect of **1** and **5**. The effects of synthetic, racemic armeniaspirol A **5** and natural, enantiopure armeniaspirol A **1** on *M. luteus* DSM1790 were determined using the *Bac*light™ assay in 384 well format. Two biological replicates were performed with two technical replicates each. Average IC_{50} values of 0.02 µg/ml and MIC values of 1.25 µg/ml for both **1** and **5** were calculated by first determining the ratio of red to green fluorescence normalised to the vehicle (DMSO) controls, and then using a non-linear fit of an inhibitor to a normalised response with Graphpad Prism.

*Bacterial membrane potential assay (Bac*light™ *assay) for E. coli ΔtolC, S. aureus and M. luteus in 96 well format*

Overnight log-phase cultures of *E. coli ΔtolC JW5503*, methicillin-sensitive *S. aureus* NCTC 8325-4 (MSSA) or *M. luteus* DSM1790 growing in tryptic soy broth, were harvested by centrifugation (4500 *g*, 5 min). The bacterial pellets were resuspended in phosphate buffered saline (PBS, pH 7.4) and the OD_{600} was adjusted to 0.5 units. The bacterial suspension was labelled by addition of the potential-sensitive dye 3,3'-diethyloxacarbocyanine iodide ($DiOC_2(3)$) to a final concentration of 20 µM. The labelling was performed for 10 min at room temperature under protection from direct light. The test compounds and controls were diluted in PBS in the range of 0.5 ng/ml to 10 µg/ml in 96-well black-walled, clear-bottom microtiter plates (100 µl per well). One hundred microliters of the labeled cell suspension were dispensed into each of the wells (final volume = 200 µl). The plates were mixed by orbital shaking (600 rpm, 15 seconds) and incubated at room temperature for 15 min protected from light. Fluorescence was recorded with a Synergy 2 Multi-Mode Reader (BioTek, USA) at λ_{ex} = 485 nm, λ_{em} = 520 nm

and λ_{ex} = 485 nm, λ_{em} = 600 nm, respectively. All measurements were done in triplicate. The membrane potential, expressed as the red/green fluorescence ratio, was calculated with respect to the DMSO-treated control.

The kinetic membrane potential assay using methicillin-resistant *S. aureus* N315 (MRSA) and *E. coli* Δ*tolC* followed a similar protocol to the one for MSSA, with the exception that the overnight culture was re-inoculated into fresh media to obtain an OD_{600} of 0.05 (approximately 2.5×10^7 CFU/ml), and bacteria were cultivated until they reached exponential phase. **1** (5 mg/ml stock in DMSO) was applied at 0.5 x MIC and 2 x MIC, and depolarization was assessed by the *Bac*light™ assay in 96 well format by monitoring the fluorescence shift of $DiOC_2(3)$. Further, a final concentration of 30 µM was used for the $DiOC_2(3)$ dye and measurements were taken at selected time-points over a period of 30 minutes. All measurements were done in triplicate and the membrane potential was quantified with respect to the DMSO-treated control.

Mitochondrial membrane depolarization assay

Mammalian cells (HeLa) were seeded in complete medium (DMEM + 10% FBS) on black-walled, clear-bottom, 96-well tissue culture plates at a density of 3×10^4 cells per well (100 µl/well) and allowed to attach to the surface overnight in a humidified incubator at 37 °C and 10% CO_2. The following day, the medium was removed and the cells were stained with 100 µl of MITO-ID MP dye loading solution (Enzo Life Sciences, Farmingdale, New York), prepared according to the manufacturer's instructions. The cells were loaded with the dye solution for 30 min at 37 ºC protected from light. The test compounds (**1**) and reference compounds (CCCP, antimycin A, oligomycin A) were added to a final concentration equal to 1 µM. The fluorescence was immediately recorded in kinetic mode at λ_{ex} = 490 nm, λ_{em} = 590 nm for 10 min with intervals of 5 seconds with a microplate reader (Infinite M200, Tecan, Switzerland). All measurements were performed in triplicate. The fluorescence values were normalized to the initial values (before treatment) and the mitochondrial membrane potential ($\Delta\Psi m$) was plotted over time.

Effects on electron transport chain

To determine whether **1** acts as an uncoupler of oxidative phosphorylation (an effect commonly observed in protonophores) or as an inhibitor of the electron transport chain, the effect on oxygen consumption in

mammalian cells was studied. Freshly trypsinized HeLa in complete medium (DMEM + 10% FBS) were placed in round-bottom OxoPlates (PreSens, Regensburg, Germany) at a density of 1.5×10^5 cells per well and treated with either 10 µM **1**, 10 µM CCCP, 1 µM rotenone, 1 µM antimycin A or 1 µM oligomycin A. Vehicle (DMSO) and no-cell controls were also included. Fluorescence intensity was continuously recorded for λ_{ex} = 540 nm, λ_{em} = 650 nm ($I_{indicator}$) and λ_{ex} = 540 nm, λ_{em} = 590 nm ($I_{reference}$) in a microplate reader (Infinite M200, Tecan, Switzerland) every 60 s for a period of 3 h using an integration start time of 0 µs and an integration time of 500 µs. The OxoPlates were calibrated according to the manufacturer's instructions, using oxygen-depleted water (treated with sodium sulfite) and oxygen-saturated water as reference values to correlate fluorescence intensity and oxygen concentration in the medium (expressed as % of oxygen saturation).

Planar lipid bilayer assays

Planar lipid bilayers (BLM, black lipid membrane) conferring to Montal and Mueller were formed as published.[6] Briefly, an aperture in a Teflon septum with a diameter of 100 µm was pre-painted with hexadecane dissolved in n-hexane at 1-5% (v/v) and the cuvette compartments were dried for 30-35 min, in-order to eliminate the solvent. The bilayers were made with 1,2- diphytanoyl-*sn*-glycero-phosphatidyl-choline at a concentration of 5 mg/ml in n-pentane.

We first measured the conductance of the bilayer membrane alone, which was negligible. After insuring a tight membrane, we added the indicated concentration of **1** or CCCP both dissolved in DMSO. Standard Ag/AgCl electrodes were used to detect the ionic current. Moreover, the cis side electrode of the cell was grounded, whereas the trans side electrode was linked to the headstage of an Axopatch 200B amplifier, used for the conductance measurements in voltage clamp mode. The signals were filtered by an on-board low pass Bessel filter at 10 kHz and with a sampling frequency of 50 kHz. Examination of the current recordings was completed using Clampfit (Axon Instruments). The current-voltage relation of the individual experiments was determined from single averaged currents at given voltages.

A

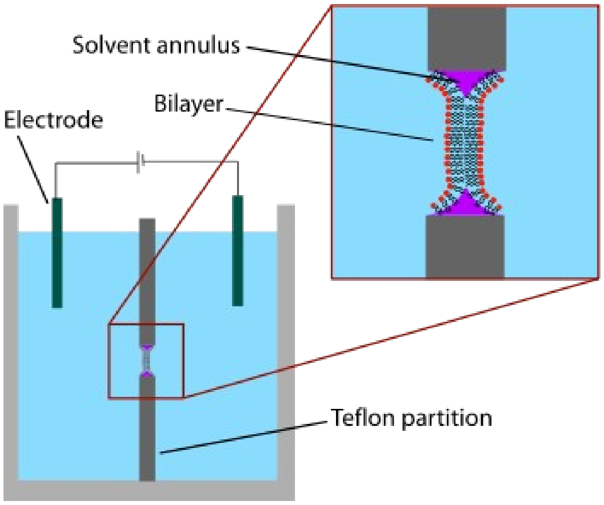

B

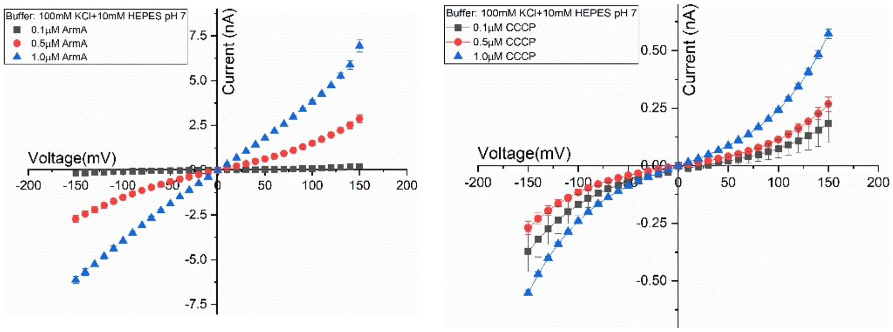

C

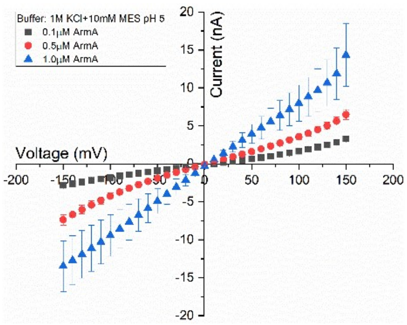

Supplementary Figure S3: Planar lipid bilayer assays. A) Schematic view of a typical lipid bilayer experiment. A membrane is folded across a small Teflon orifice of about 100 mm. A voltage is applied and the ion current is recorded (I/V curve). Note that the membrane represents the main resistance. B) Ion conductance across a planar lipid bilayer. Reducing the KCl concentration in the buffer from 1M (see Figure 3) to 0.1 M KCl as shown here did not change the conductance. The buffer also contained 10 mM HEPES pH 7. Left panel: **1** and right panel: CCCP. C) Variation of the pH from pH 7 (see Figure 3) to pH5 as shown here changed the conductivity, indicating the strong role of protons. The buffer contained 1 M KCl 10 mM HEPES.

Vesicular lipid bilayers

Preparation of large unilamellar vesicles

To analyze the proton translocation capability of Armeniaspirol A, large unilamellar vesicles (LUVs) either filled with the fluorescent dye pyranine (8-hydroxypyrene-1,3,6-trisulfonic acid) or 5(6)-carboxyfluorescein were used. A lipid film was obtained by drying a POPC (1-palmitoyl-2-oleoyl-*sn*-glycero-3-phosphocholine, Avanti Polar Lipids) solution (2 mg total lipid mass in chloroform) under a gentle nitrogen stream at 30 °C. Residual solvent was removed *in vacuo* for 3 h and lipid films were stored at 4 °C until use. The film was rehydrated in 1 mL buffer (100 mM carboxyfluorescein, 20 mM HEPES, pH 7.4 or 100 mM KCl, 10 mM HEPES, 0.5 mM pyranine, pH 7.4) for 30 min and then thoroughly vortexed for 3 x 30 s at 5 min intervals to obtain multilamellar vesicles. The suspension was extruded 31 times through a polycarbonate membrane with a nominal pore diameter of 200 nm using a mini-extruder (LiposoFast-Basic, Avestin). Extravesicular dye was removed via size exclusion chromatography (Illustra NAP-25 G25 column, GE Healthcare) in the respective buffer (275 mM KCl, 20 mM HEPES, pH 7.4 for carboxyfluorescein or 100 mM KCl, 10 mM HEPES, pH 7.4 for pyranine). Subsequently, the final phospholipid concentration of the suspension was determined by quantifying inorganic phosphate content. For this, 20 µL of the vesicle suspension were mixed with 200 µL perchloric acid (70% *w*/*v*) and heated to 240 °C for 30 min together with calibration samples of known phosphate concentration. The residual material was dissolved in 700 µL of an aqueous solution of perchloric acid (12.6%, *w*/*v*) with ammonium orthomolybdate (0.45% *w*/*v*) and 700 µL of ascorbic acid (1.7% *w*/*v*). After incubation of the solutions for 10 min at 80 °C, the absorption of the samples at λ = 820 nm was measured photometrically (Spectrophotometer V-650, Jasco). Phospholipid concentration was finally calculated from the calibration curve.

Carboxyfluorescein self-quenching assay

All fluorescence spectroscopic experiments were conducted with a FP-6500 spectrofluorometer (Jasco) at 22 °C using a 10 mm x 4 mm quartz cuvette (Hellma) under constant stirring. LUVs constituted of POPC and filled with carboxyfluorescein were diluted in buffer (275 mM KCl, 20 mM HEPES, pH 7.4) to a final volume of 792 µL and a phospholipid concentration of 50 µM. Changes in fluorescence intensity were recorded in a time-dependent manner with an excitation wavelength λ_{ex} = 480 nm, an emission wavelength λ_{em} = 520 nm and band widths of 3 nm. After monitoring a baseline for 100 s, 8 µL of 500 µM

armeniaspirol A in DMSO were added (compound to lipid ratio (C:L) 1:10). After 500 s, vesicles were lysed completely by adding 13 µL 3% (*w*/*v*) LDAO (lauryldimethylamine oxide). All data points were normalized to the fluorescence intensity directly before addition of the compound and after vesicle lysis.

Proton translocation assay

To analyze proton transport across lipid membranes, LUVs composed of POPC and filled with the fluorescent dye pyranine at pH 7.4 were suspended in the corresponding buffer (100 mM KCl, 10 mM HEPES) at pH 6.4 or 8.4, respectively, to a final phospholipid concentration of 50 µM and a total volume of 792 µL. Pyranine fluorescence intensity was recorded in a time-dependent manner with λ_{ex} = 480 nm, λ_{em} = 520 nm and band widths of 3 nm. After the acquisition of a baseline for 100 s, 8 µL of 50 to 500 µM solutions of **1** in DMSO were added and changes in fluorescence intensity were monitored (C:L 1:100, 1:50 and 1:10). Acidification of the vesicular lumen resulted in a fluorescent quenching, whereas proton efflux was indicated by an increase in pyranine fluorescence. After 500 s, 13 µL 3% LDAO (*w*/*v*) were added leading to complete vesicle lysis and disruption of the pH gradient. All data points were normalized to the fluorescence intensity directly before addition of the compound and after vesicle lysis.

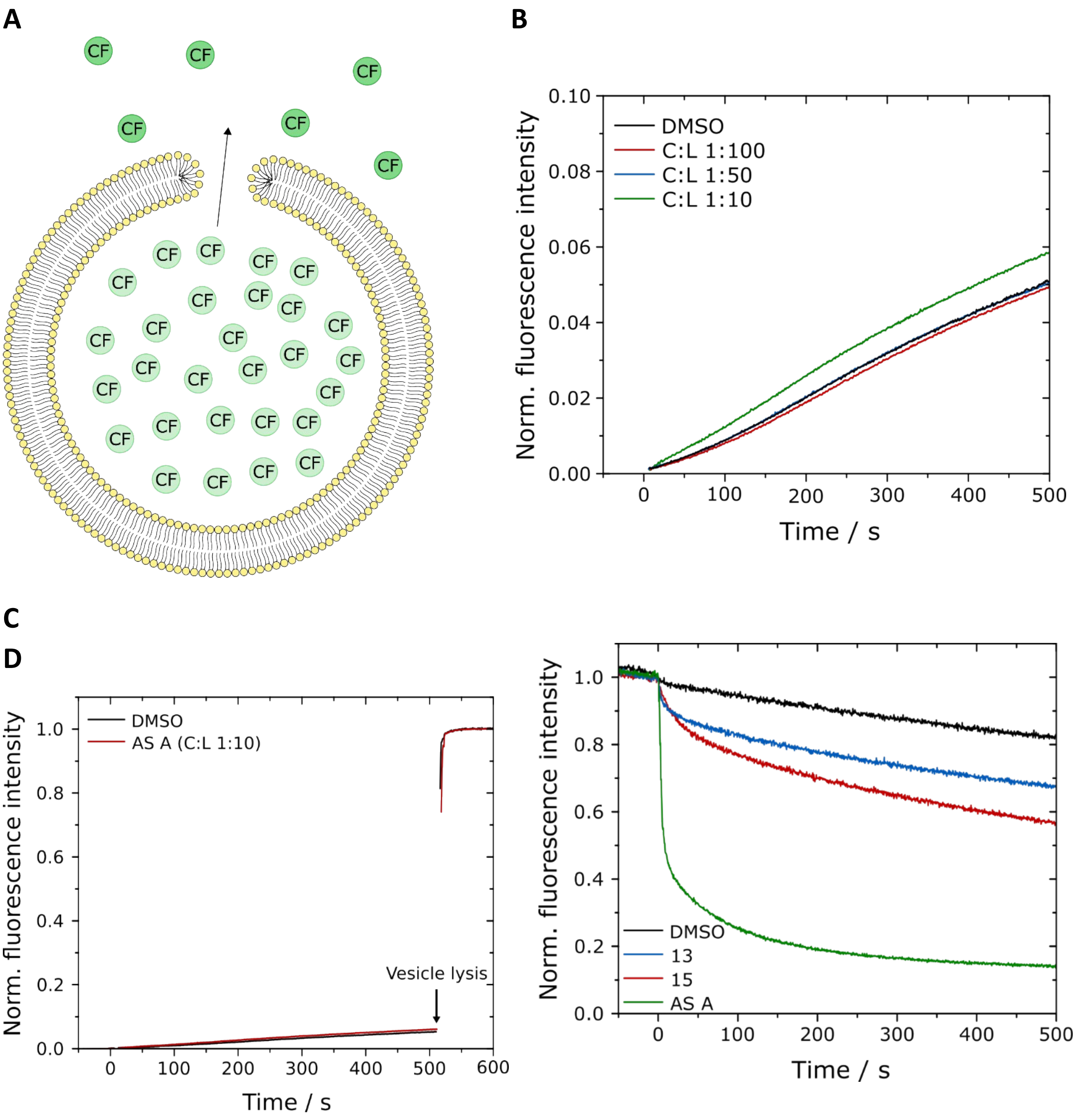

Supplementary Figure S4: Experiments with vesicular lipid bilayers.

A) Principle of carboxyfluorescein leakage assay (CF = carboxyfluorescein). B) Absence of CF leakage at different compound-to-lipid ratios C:L in presence of **1**. C) Normalized carboxyfluorescein fluorescence intensity upon addition of 5 µM **1** (= AS A) (C:L = 1:10). Vesicle lysis was induced by LDAO. Vesicles were composed of POPC and filled with 100 mM CF, 20 mM HEPES, pH 7.4. D) Normalized pyranine fluorescence intensity upon addition of 5 µM **1** (= AS A), **13** and **15** (C:L 1:10) with proton influx from pH 6.4 to pH 7.4. Vesicles were composed of POPC

and filled with 100 mM KCl, 10 mM HEPES, 0.5 mM pyranine, pH 7.4 and diluted in buffer with pH 6.4.

In vitro **resistance development, frequency of resistance, and resistance level of mutants**

A stored glycerol stock was streaked out on a CASO agar and incubated overnight in a static incubator at 37 °C. The following day a fresh culture was prepared by inoculating a single colony in fresh Mueller-Hinton broth (MHB) and incubated at 37 °C at 180 rpm for approximately 16-18 hours. The OD_{600} of the overnight culture was determined and a total of 5 x 10^9 CFU/plate was confluently spread over the surface of the agar plate containing 4x MIC (8 µg/mL) of **1** and **2**, respectively, until it was completely soaked in. The plate was incubated in a static incubator for 24 hours at 37 °C. The obtained resistant clones were counted to determine the frequency of resistance (number of resistant colonies divided by the number of viable colonies of the initial inoculum), and the armeniaspirol-resistant mutants were further assessed by determining their MIC shift and mutant genotypes.

Supplementary Table S2: MICs **of armeniaspirol A (1) and armeniaspirol B (2) obtained from wild-type and resistant mutants of the** *E. coli* K12 Δ*tolC* strain.

**Strain**	**MIC (µg/mL)**	
	Armeniaspirol A (**1**)	Armeniaspirol B (**2**)
E. coli* Δ*tolC	2	2
***E. coli* Δ*tolC* 1^R mutant #1**	>64	>64
***E. coli* Δ*tolC* 1^R mutant #2**	>64	>64
***E. coli* Δ*tolC* 1^R mutant #3**	>64	>64
***E. coli* Δ*tolC* 1^R mutant #4**	>64	>64
***E. coli* Δ*tolC* 1^R mutant #5**	>64	>64
***E. coli* Δ*tolC* 1^R mutant #6**	>64	>64
***E. coli* Δ*tolC* 1^R mutant #7**	>64	>64
***E. coli* Δ*tolC* 1^R mutant #8**	>64	>64
***E. coli* Δ*tolC* 1^R mutant #9**	>64	>64
***E. coli* Δ*tolC* 1^R mutant #10**	>64	>64
***E. coli* Δ*tolC* 1^R mutant #11**	>64	>64
***E. coli* Δ*tolC* 1^R mutant #12**	>64	>64
***E. coli* Δ*tolC* 1^R mutant #13**	>64	>64
***E. coli* Δ*tolC* 2^R mutant #14**	>64	>64
***E. coli* Δ*tolC* 2^R mutant #15**	>64	>64

Whole-Genome Sequencing

Total DNA of selected resistant clones and wild type control samples were subjected to whole-genome sequencing on Illumina MiSeq platform at the Helmholtz Centre for Infection Research (Braunschweig, Germany). Libraries were constructed according to paired-end protocol and subsequently sequenced to a total read length of 2 x 300bp. The raw data was then mapped to a reference sequence of *E. coli* K12 Δ*tolC*, GenBank accession number CP018801. Geneious Prime version 2021.1.1 with default settings was used for reference-guided sequence assembly, subsequent variant calling and data analysis.

Supplementary Table S3: List of mutations obtained from spontaneous resistant development of *E. coli* K12 Δ*tolC* strain. Mapped to CP018801.[7]

**Mutant number (see table S2)**	**Mutations determined in comparison to the parent genome**					
	mdtO (multidrug resistance protein)	Intergenic region downstream of *csrA* and upstream of tRNA-serine	*prfB* (Peptide chain release factor)	Intergenic region upstream *cvpA* (inner membrane protein)	*fdoG* (Formate dehydrogenase-O major subunit)	*plsB* (membrane-bound glycerol-3-phosphate acyltransferase)
**1**	S2K; A3T, L4I; N5G; S6T; L7E; L9C		T173S			
**2**	S2K; A3K, L4I; N5G; P8K; L9C		T173S			
**3**	S2R; P8K; L9C		T173S			
**4**	S2R; N5S; L7V; P8K; L9C		T173S			

**5**	S2K; A3K L4I; N5G; S6K; L7E; P8K; L9C		T173S			
**6**	S2K; A3T L4I; N5G; S6K; L7E; P8K; L9C		T173S			
**7**		G>A (Position: 1067334)				
**8**		G>A (Position: 1067334)				
**9**		G>A (Position: 1067334)				
**10**		G>A (Position: 1067334)				
**11**		G>A (Position: 1067334)				
**12**		G>A (Position: 1067334)				
**13**		G>A (Position: 1067334)				
**14**				G>A (Position: 1455434)	N144K	
**15**						A204P; R207G

Relevance of observed mutations

The observed single point mutations for armeniaspirol B (**2**) in the intergenic region upstream of *cvpA* and *fdoG* as well as *plsB* can all possibly be linked to the stringent response that is a stress response that can lead to the obtained resistance. Guanosine 5′-(tri)diphosphate 3′-diphosphate [(p)ppGpp] is an alarmone that is produced during the stringent response, and bacterial resistance mechanisms have been reported in several species and against a range of antimicrobials.[8]

The *cvpA* gene is responsible for Colicin V secretion as well as the activation of the stress response pathway, which promotes membrane potential homeostasis.[9] Cho and co-workers (2021) found that *cvpA* is upregulated during the stringent response in the absence of MDR efflux systems, which is mostly regulated by the RpoS sigma factor. The RpoS sigma factor plays a major role in controlling gene

expression during the stationary growth phase. The *fdoG* gene protects *E. coli* cells against antimicrobial peptides in the stationary-phase.[10] The protection is RpoS independent, however, mediated by BipA GTPase (BPI-inducible protein A) that is dependent on oxidation by a formate dehydrogenase. The BipA GTPase has been described in several cellular processes including antimicrobial resistance against a variety of antimicrobials.[11]

The *plsB* gene is responsible to catalyze the first step in the phospholipid biosynthesis and can be linked to the stringent response as it is a proposed target of (p)ppGpp.[12] Inhibition occurs during the production of (p)ppGpp which interferes with membrane-associated steps in peptidoglycan biosynthesis, which can allow for resistance.[13] The interaction between these genes and the stringent response should still be further investigated, however, the obtained resistance linked with the mutations of these genes could possibly describe an efflux-independent mode of resistance against armeniaspirols.

Cytotoxicity testing

Resazurin assay

The cytotoxicity testing was performed by monitoring resazurin reduction. Stock solutions of the test compounds and controls were prepared in DMSO. Exponentially growing mouse fibroblasts (L929 cells) and human lung carcinoma cells (A549) were diluted with Dulbecco's Modified Eagle's Medium (DMEM) supplemented with 10% fetal bovine serum (FBS) to a final density of 5×10^4 cells/ml. One hundred microliters of the diluted cell suspension were seeded into sterile, polystyrene, 96-well tissue culture plates (5000 cells/well). The cells were allowed to adhere to the surface of the plate overnight in an incubator at 37 ºC, 10% CO_2 and 95% relative humidity. Serial dilutions of the test compounds were prepared in DMEM supplemented with 10% FBS using a dilution factor of 2 in the concentration range of 0.04 μg/ml to 20 μg/ml. One hundred microliters of the diluted compounds were added to the wells of the plates containing the cells. The plates were incubated at 37ºC in a humidified atmosphere (10% CO_2, 95% relative humidity) for 72 h. After the incubation, 20 µl of the resazurin solution (500 μM) were dispensed into every well. The plates were further incubated for 4 h at 37 ºC to allow reduction of resazurin into fluorescent resofurin by viable cells. The fluorescence was recorded using λ_{ex} = 560 nm and λ_{em} = 590 nm with a Synergy 4 plate reader (BioTek, Bad Friedrichshall, Germany). The cell viability was calculated by normalizing the fluorescence of untreated cells to 100% viability and then calculating the percentage of viable cells from the measured fluorescence. Viability was plotted as a function of test

compound concentration. The resulting data was fitted to a three-parameter equation using GraphPad Prism 5 and the IC_{50} values were calculated. All measurements were performed using four replicates.

MTT (3-(4,5-dimethylthiazol-2-yl)-2,5-diphenyltetrazolium bromide) assay

L-929 mouse fibroblasts, A549 human lung carcinoma cells (ACC 107) and KB3.1 human cervix carcinoma cells (ACC 158) obtained from the DSMZ were cultivated at 37 °C and 10 % CO_2 in DME medium (high glucose) supplemented with 10 % fetal calf serum. Human umbilical vein endothelial cells (HUVECs, Promocell c-12203) were cultivated in EBM-2 medium (Lonza), accordingly. Cell culture reagents came from Life Technologies Inc. (GIBCO BRL). 60 µL of serial dilutions of the test compound were given to 120 µL of suspended cells (50,000/mL) in wells of 96-well plates. After 5 days of incubation growth inhibition was determined using an MTT assay.

Supplementary Table S4: Cytotoxic activity of 1 and CCCP on selected murine and human cell lines.

IC_{50} [µM]							
**Cell line**		L929	KB3.1	HUVEC	HeLa	L929	A549
**Compound**	**1**	6	3,1	2,1	0,8	9,2	11,5
	CCCP	4	3,4	3,9	1,2	6,2	7,3
**Assay type**		MTT				Resazurin	

ATP-based acute toxicity assay

To assess acute toxicity effects, the ATP content was quantified as followed: 2.4 x 10^4 murine L929 cells were seeded in white-walled clear bottom 96 well plates (Greiner #655098) in DMEM (Gibco #61965-026) high-glucose, 10% FBS (Gibco #10500-064) and incubated to adhere at 10% CO_2, 37°C, 95% relative humidity overnight. After 24h, the cell medium was removed and replaced with compounds diluted in medium. Stock solutions of 1% Triton X-100 in water and **5** (5.2 mM) or **15** (5 mM) in DMSO were serially diluted in 1:3 steps. Solvent was also diluted for a negative control. The assay was performed in technical duplicate, with biological duplicates. After 1 or 3 hours incubation at 37°C, an equal volume of CellTiter-Glo® solution (Promega®) was added to the cells. After 10 min incubation at room temperature on an orbital shaker (900 rpm) in the dark, the luminescence was measured on an Infinite M200 PRO reader

(TECAN). The relative luminescence was calculated by normalizing to the solvent control. An acute toxicity of **5** was observed already after 1 h and slightly increased after 3h (Supplementary Figure S5). The effect was about 40x lower than the effect observed after 5d in the MTT test, as both the direct toxicity and loss of cell proliferation account for the long-term decrease of MTT. The highest compound concentration without a solvent effect was 580 µM, which was not high enough to record a complete dose-response curve. The IC_{50} of **5** could therefore not be accurately calculated, but was estimated to be at 200-300 µM. The graphs represent an exemplary result from technical duplicates of 2 independent biological replicates.

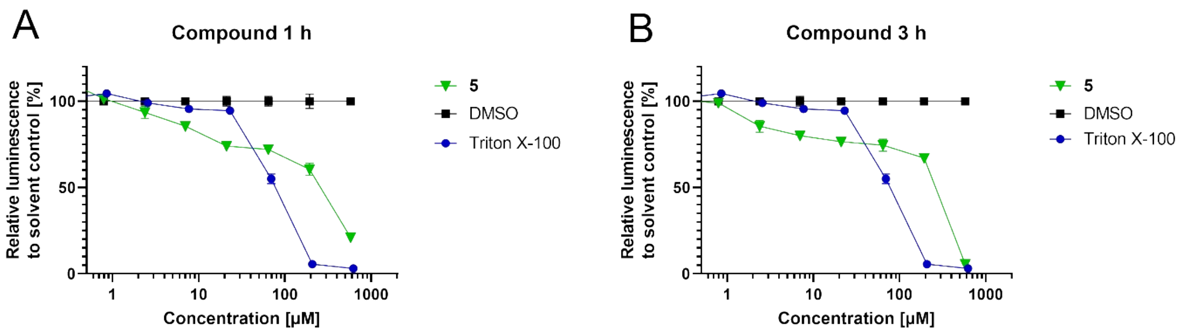

Supplementary Figure S5: Acute cytotoxicity of **5**, as assessed by cellular ATP content. L929 cells (2 x 10^5 cells/ml) were incubated with the indicated concentrations of **5** (green inverted triangles), DMSO (black squares) or Triton X-100 (blue circles) for 1h (A) and 3h (B). The ATP content was quantified by a CellTiter-Glo® assay. The luminescence was measured and normalized to the solvent control.

Flow cytometry assay

Jurkat cells (10^5 cells/mL) were incubated with different concentrations of **1**, the protonophores CCCP and Carbonyl cyanide-4-(trifluoromethoxy)phenylhydrazone (FCCP), and H_2O_2 for 48 h in RPMI 1640 medium supplemented with 10% FBS at 37 ºC and 10% CO_2. Cell viability was determined by flow cytometry through counting live cells labeled with the membrane-permeable SYTO 9 dye versus dead cells labeled with ethidium propionate. The assay was conducted using a commercial kit (Muse® Count and Viability Assay Kit, Luminex, USA) with a Guava® Muse® cell analyzer (Luminex Corp., USA) according to the manufacturer's protocol.

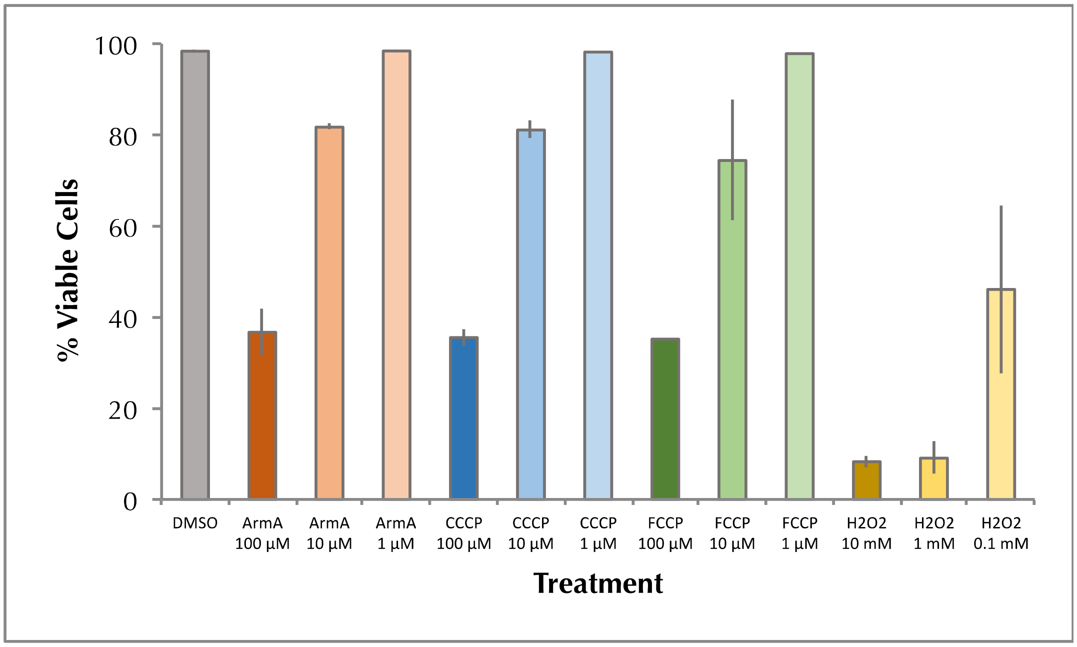

Supplementary Figure S6: Cytotoxicity of **1** in Jurkat cells. Jurkat cells (10^5 cells/mL) were incubated with the indicated concentrations of **1** (= ArmA), the protonophores CCCP and FCCP, or H_2O_2 for 48 h, and viability was determined using a live/dead stain and a flow cytometry readout.

Experiments with *E. coli* ClpP and ClpXP

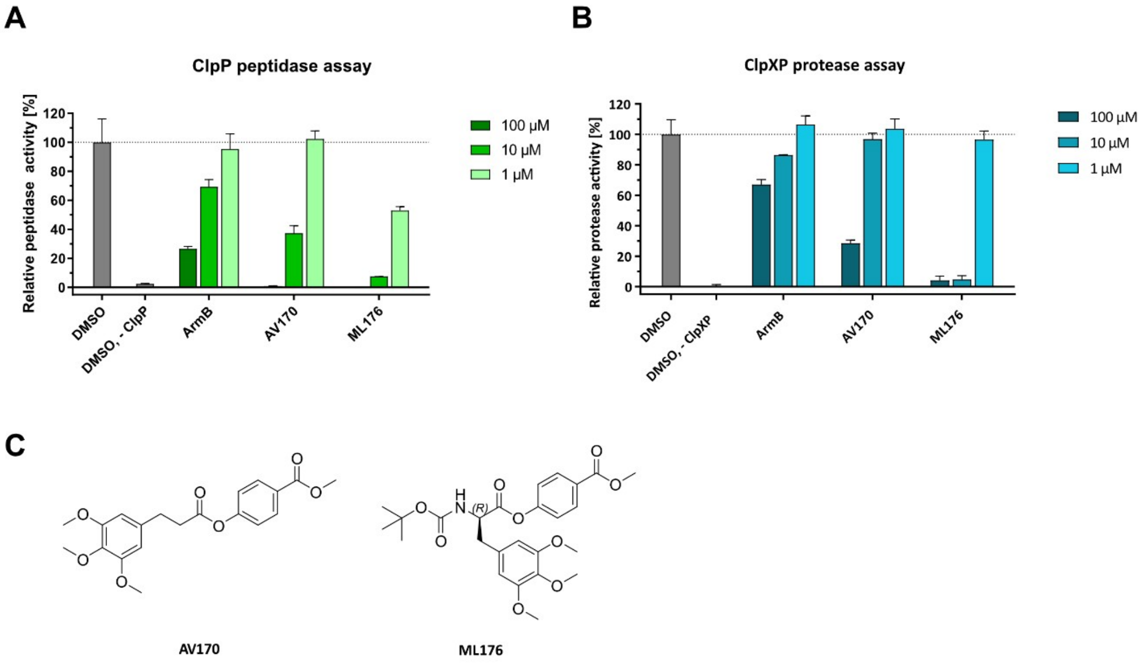

Supplementary Figure S7: *E. coli* ClpP peptidase (A) and ClpXP protease (B) assays. Inhibition by armeniaspirol B (**2**, ArmB) was measured together with previously reported *S. aureus* ClpP inhibitors AV170 and ML176[14, 15] (**C**) as controls.

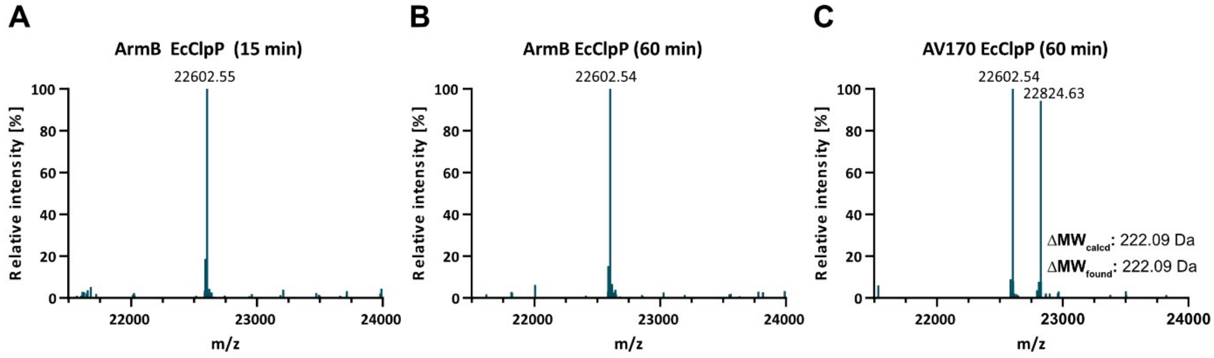

Supplementary Figure S8: Intact protein mass spectra of *E. coli* ClpP incubated with armeniaspirol B (**2**, ArmB) or AV170. No covalent modification by **2** was observed after up to 1 hour incubation (**A, B**), whereas AV170 covalently modifies ClpP (**C**).

Protein purification

E. coli ClpP (EcClpP) was kindly provided by Dr. Anja Fux and purified as described previously.[16] eGFP-ssrA was kindly provided by Dr. Markus Lakemeyer and purified as described previously.[15] *E. coli* ClpX (EcClpX) was expressed and purified as described previously.[14, 17] In short, EcClpX was overexpressed in *E. coli* (DE3) Rosetta 2 cells in LB media at 25 °C for 20 h after induction with 0.5 mM IPTG. After harvest, lysis by sonication (lysis buffer: 50 mM HEPES pH 7.6, 300 mM KCl, 15 % glycerol, 1 mM DTT, 10 mM imidazole, 5 mM $MgCl_2$) and cell debris removal, affinity chromatography on a ÄKTA Purifier 10 (GE Healthcare) and a preequilibrated 5 ml HisTrap HP column (GE Healthcare) was performed. TEV cleavage (2.5 mg/ml) was performed overnight at 10 °C until intact protein mass spectrometry (ip-MS) indicated complete conversion. The mixture was concentrated and then loaded onto a Superdex 200pg 16/60 (GE Healthcare) equilibrated in lysis buffer without imidazole. Eluted appropriate fractions were pooled, concentrated, frozen in liquid nitrogen and stored at -80 °C.

Peptidase assay

1 µl of compound (100x stocks in DMSO) or DMSO as a control were added to a black flat-bottom 96-well plate and mixed with 98 µL of enzyme mix (10 nM final *E. coli* $ClpP_{14}$ concentration) in PZ buffer (25 mM HEPES pH 7.6, 200 mM KCl, 5 mM $MgCl_2$, 1 mM DTT, 10 % glycerol). The plate was incubated at 30 °C for 15 min after which the reaction was started by adding 1 µl fluorogenic substrate Ac-Ala-hArg-(*S*)-2-aminooctanoic acid-7-amino-4-carbamoylmethylcoumarin (Ac-Ala-hArg-2-Aoc-ACC, custom-synthesis by Bachem) (20 mM stock in DMSO; 200 µM final substrate concentration). Fluorescence (λ_{ex} = 380 nm; λ_{em} = 430 nm) was measured at 30 °C with an Infinite M Nano$^+$ plate reader (Tecan). Data were recorded in triplicates and two independent experiments were performed. Peptidase activity was determined by linear regression of the initial slopes via GraphPad Prism, DMSO-treated control samples were normalized to 100% activity.

Protease assay

In vitro inhibition of *E. coli* ClpXP protease activity was measured by monitoring the degradation of eGFP-ssrA, a fluorescent substrate tagged by a short ssrA-sequence for ClpXP-mediated degradation similarly as described previously.[15, 18] 0.6 µL compound (100x stocks in DMSO) or DMSO as control were added to a black flat-bottom 96-well plate. 58.4 µL of enzyme mix with an ATP regeneration system (final concentrations: 0.10 µM $ClpP_{14}$, 0.20 µM $ClpX_6$, ATP-regeneration system: 4 mM ATP, 16 mM creatine phosphate, 20 U/mL creatine phosphokinase in PZ buffer (25 mM HEPES pH 7.6, 200 mM KCl, 5 mM $MgCl_2$, 1 mM DTT, 10 % glycerol)) was added and the mixture was incubated at 32 °C for 15 min. The reaction was started by addition of eGFP-ssrA (final concentration 0.9 µM) and fluorescence (λ_{ex} = 485 nm; λ_{em} = 535 nm) was measured at 32 °C with an Infinite M Nano$^+$ plate reader (Tecan). Protease activity was determined by linear regression of the initial slopes via GraphPad Prism, DMSO-treated control samples were normalized to 100% and DMSO-treated samples lacking ClpXP to 0 % activity. Data were recorded in triplicates and two independent experiments were performed.

Intact protein mass spectrometry

High-resolution intact protein mass spectrometry was performed in order to detect a covalent modification of EcClpP. 1 µM EcClpP was incubated with 100 µM AV170 or Armeniaspirol B (100x stocks in DMSO) in PZ buffer (25 mM HEPES pH 7.6, 200 mM KCl, 5 mM $MgCl_2$, 1 mM DTT, 10 % glycerol) for 15 min or 1 hour at 32 °C. Measurements were performed on a Dionex Ultimate 3000 HPLC system coupled to an LTQ FT Ultra (Thermo) mass spectrometer with an electrospray ionization source (spray voltage 4.0 kV, tube lens 110 V, capillary voltage 48 V, sheath gas 60 a.u., aux gas 10 a.u., sweep gas 0.2 a.u.). 5 µl of reaction mixture were on-line desalted using a Massprep desalting cartridge (Waters). The mass spectrometer was operated in positive ion mode collecting full scans at high resolution (R = 200,000) from m/z 600 to m/z 2000. The protein spectra were deconvoluted using the Thermo Xcalibur Xtract algorithm.

Calculation of pK_a values and other physicochemical properties of chloropyrrole-containing natural products

To be considered as a protonophore, a substance should have a pK_a value close to the physiological pH as well as good membrane permeability. Three programs were used to calculate the pK_a values with different methods. While Schrödinger's Epik[19, 20] uses Hammett and Taft methods in conjunction with ionization and tautomerization tools, Jaguar[21] utilizes quantum-chemical methods. ChemAxon's calculation method is based on micro and macro dissociation constants. The obtained values suggest that all compounds except (±)-deoxy-armeniaspirole-A (**13**) have a functional group with a pK_a value of 5.5 - 8.5. CCCP and 1-(4-Chlorophenyl)-4,4,4-trifluoro-3-hydroxy-2-buten-1-one served as reference compounds for the pK_a predicitons.

The polar surface area is a commonly used metric for optimizing cell permeability of drugs. It represents the surface sum of all *O*- and *N*-atoms including their attached hydrogen atoms and can be quickly estimated by the topological polar surface area (TPSA) without 3D structures. Compounds with TPSA values below 140 $Å^2$ have a good chance of being cell permeable[22], which is the case for all compounds in the scope of this study.

LogD values are another surrogate parameter for the prediction of passive membrane permeability. Especially for very polar compounds with a logD value < 0, membrane permeability may be limited, which is not the case for all compounds in the scope of this study.

The pK_a values were calculated with the following program versions and settings:

Schrödinger Release 2018-4, Epik, Schrödinger, LLC, New York, NY, 2018. Analysis Mode: Sequential pK_a values; Solvent: H2O; pH: 7.0; Generate tautomers: yes; Include original tautomer: no.

Schrödinger Release 2018-4, Jaguar, Schrödinger, LLC, New York, NY, 2018. Standard Settings were used.

MarvinSketch 20.11.0, ChemAxon Ltd. Mode: macro; Acid/base prefix: static; Min basic pK_a: -2; Max acidic pK_a: 16; Temperature (K): 298; Use correction library: no; Consider tautomerization / resonance: yes; Show distribution chart: yes.

The topological polar surface areas (TPSA) were calculated with ChemDraw 19.0.0.22, PerkinElmer Informatics, Inc.

The logD values were calculated with LogD Predictor, ChemAxon Ltd.

Supplementary Table S5: Structures, calculated pK_a values and other physicochemical properties of chloropyrrole-containing natural products. The colors indicate the deprotonation sites.

Compound	Calculated pK_a			pK_a (Lit.)	TPSA [Å^2]	calc. logD at pH 7
	Epik	Jaguar	MarvinSketch			
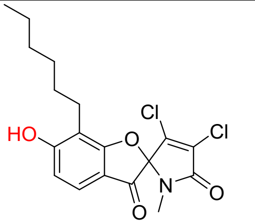 (±)-Armeniaspirole-A (**5**)	8.28 ± 1.06	6.2	7.95	/	66.8	4.58
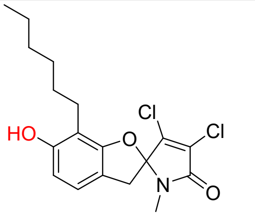 (±)-Deoxy-Armeniaspirole-A (**13**)	10.26 ± 1.06	10.0	9.77	/	49.8	4.87
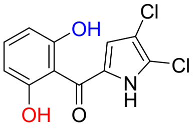 Pyoluteorin	7.21 ± 0.86	/	6.93<br>8.23	/	69.6	3.7
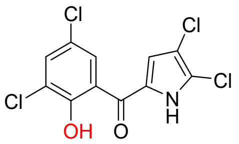 Pyrrolomycin C	5.95 ± 0.75	/	5.11	/	49.3	3.24
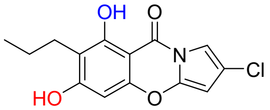 Streptopyrrole-1Cl	7.94 ± 1.06<br>8.80 ± 1.06	7.4<br>9.7	7.75<br>9.27	/	70.0	ca. 3.16
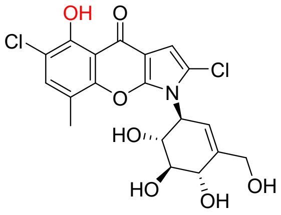 Pyralomicin C	7.99 ± 1.06	7.9	7.31	/	130.7	ca. 1.45

Compound	Calculated pK_a			pK_a (Lit.)	TPSA [Å^2]	calc. logD at pH 7
	Epik	Jaguar	MarvinSketch			
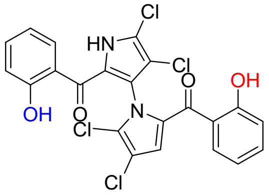 (±)-Marinopyrrole-A	7.84 ± 0.68<br>7.95 ± 0.68	10.4<br>11.2	6.78<br>7.38	/	89.9	6.79
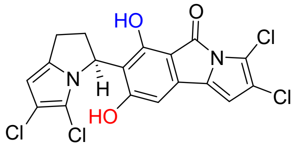 (-)-Chlorizidine-A	6.63 ± 1.06<br>9.30 ± 1.06	6.6	7.90<br>9.41	/	64.0	ca. 4.35
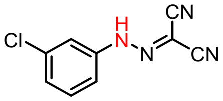 Carbonyl cyanide m-chlorophenyl hydrazine (CCCP)	6.04 ± 0.81	6.0	5.81	5.95[23]	72.0	1.82
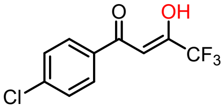 1-(4-Chlorophenyl)-4,4,4-trifluoro-3-hydroxy-2-buten-1-one	5.90 ± 2.22	5.3	4.55	6.25[24, 25]	37.3	1.02

References

1. C. Dufour, J. Wink, M. Kurz, H. Kogler, H. Olivan, S. Sable, W. Heyse, M. Gerlitz, L. Toti, A. Nusser, A. Rey, C. Couturier, A. Bauer and M. Bronstrup, *Chemistry*, 2012, **18**, 16123-16128.
2. C. Fu, F. Xie, J. Hoffmann, Q. Wang, A. Bauer, M. Brönstrup, T. Mahmud and R. Müller, *ChemBioChem*, 2019, **20**, 764-769.
3. C. Couturier, A. Bauer, A. Rey, C. Schroif-Dufour and M. Broenstrup, *Bioorganic & medicinal chemistry letters*, 2012, **22**, 6292-6296.
4. Z. Huang, L. Jin, Y. Feng, P. Peng, H. Yi and A. Lei, *Angew. Chem. Int. Ed.*, 2013, **52**, 7151-7155.
5. R. J. Lambert and J. Pearson, *J. Appl. Microbiol.*, 2000, **88**, 784-790.
6. M. Montal and P. Mueller, *Proc. Natl. Acad. Sci. U.S.A.*, 1972, **69**, 3561-3566.
7. J. Donner, M. Reck, B. Bunk, M. Jarek, C. B. App, J. P. Meier-Kolthoff, J. Overmann, R. Müller, A. Kirschning and I. Wagner-Döbler, *mSphere*, 2017, **2**.
8. J. Wu, Q. Long and J. Xie, *J. Cell Physiol.*, 2010, **224**, 300-304.
9. A. R. Warr, R. T. Giorgio and M. K. Waldor, *J. Bacteriol.*, 2020, **203**.
10. H. C. Barker, N. Kinsella, A. Jaspe, T. Friedrich and C. D. O'Connor, *Mol. Microbiol.*, 2000, **35**, 1518-1529.
11. A. Neidig, A. T. Yeung, T. Rosay, B. Tettmann, N. Strempel, M. Rueger, O. Lesouhaitier and J. Overhage, *BMC Microbiol.*, 2013, **13**, 77.
12. T. J. Larson, V. A. Lightner, P. R. Green, P. Modrich and R. M. Bell, *J. Biol. Chem.*, 1980, **255**, 9421-9426.
13. K. Poole, *J. Antimicrob. Chemother.*, 2012, **67**, 2069-2089.
14. M. W. Hackl, M. Lakemeyer, M. Dahmen, M. Glaser, A. Pahl, K. Lorenz-Baath, T. Menzel, S. Sievers, T. Böttcher, I. Antes, H. Waldmann and S. A. Sieber, *J Am Chem Soc*, 2015, **137**, 8475-8483.
15. M. Lakemeyer, E. Bertosin, F. Möller, D. Balogh, R. Strasser, H. Dietz and S. A. Sieber, *Angew Chem Int Ed Engl*, 2019, **58**, 7127-7132.
16. A. Fux, V. S. Korotkov, M. Schneider, I. Antes and S. A. Sieber, *Cell Chem Biol*, 2019, **26**, 48-59.e47.
17. T. F. Gronauer, M. M. Mandl, M. Lakemeyer, M. W. Hackl, M. Meßner, V. S. Korotkov, J. Pachmayr and S. A. Sieber, *Chem Commun (Camb)*, 2018, **54**, 9833-9836.
18. Y. I. Kim, R. E. Burton, B. M. Burton, R. T. Sauer and T. A. Baker, *Mol Cell*, 2000, **5**, 639-648.
19. J. R. Greenwood, D. Calkins, A. P. Sullivan and J. C. Shelley, *J. Comput. Aided Mol. Des.*, 2010, **24**, 591-604.
20. J. C. Shelley, A. Cholleti, L. L. Frye, J. R. Greenwood, M. R. Timlin and M. Uchimaya, *J. Comput. Aided Mol. Des.*, 2007, **21**, 681-691.
21. A. D. Bochevarov, E. Harder, T. F. Hughes, J. R. Greenwood, D. A. Braden, D. M. Philipp, D. Rinaldo, M. D. Halls, J. Zhang and R. A. Friesner, *Int. J. Quantum Chem.*, 2013, **113**, 2110-2142.
22. H. Pajouhesh and G. R. Lenz, *NeuroRx*, 2005, **2**, 541-553.
23. P. G. Heytler, *Biochemistry*, 1963, **2**, 357-361.
24. J. R. Jones and S. P. Patel, *J. Chem. Soc. Perkin Trans. 2*, 1975.
25. N. Tharmalingam, E. Jayamani, R. Rajamuthiah, D. Castillo, B. B. Fuchs, M. J. Kelso and E. Mylonakis, *Future Med. Chem.*, 2017, **9**, 1401-1411.
